# Human mesenchymal stem cell-engineered hepatic cell sheets accelerate liver regeneration in mice

**DOI:** 10.1038/srep16169

**Published:** 2015-11-10

**Authors:** Noriko Itaba, Yoshiaki Matsumi, Kaori Okinaka, An Afida Ashla, Yohei Kono, Mitsuhiko Osaki, Minoru Morimoto, Naoyuki Sugiyama, Kazuo Ohashi, Teruo Okano, Goshi Shiota

**Affiliations:** 1 Division of Molecular and Genetic Medicine, Graduate School of Medicine, Tottori University, 86 Nishi-cho, Yonago, Tottori 683-8503, Japan; 2 Division of Pathological Biochemistry, Department of Biomedical Sciences, Faculty of Medicine, Tottori University, 86 Nishi-cho, Yonago, Tottori 683-8503, Japan; 3 Research Center for Bioscience and Technology, Tottori University, 4-101, Koyama-cho Minami, Tottori 680-8550, Japan; 4 Department of Molecular and Cellular BioAnalysis, Graduate School of Pharmaceutical Sciences, Kyoto University, 6-29 Yoshida-Shimoadachi-cho, Sakyo-ku, Kyoto 606-8501, Japan; 5 Laboratory of Drug Development and Science, Graduate School of Pharmaceutical Sciences, Osaka University, 1-6 Yamada-Oka, Suita, Osaka 565-0871, Japan; 6 Institute of Advanced Biomedical Engineering and Science, Tokyo Women’s Medical University. 8-1 Kawada-cho, Shinjuku-ku, Tokyo 162-8666, Japan

## Abstract

Mesenchymal stem cells (MSCs) are an attractive cell source for cell therapy. Based on our hypothesis that suppression of Wnt/β-catenin signal enhances hepatic differentiation of human MSCs, we developed human mesenchymal stem cell-engineered hepatic cell sheets by a small molecule compound. Screening of 10 small molecule compounds was performed by WST assay, TCF reporter assay, and albumin mRNA expression. Consequently, hexachlorophene suppressed TCF reporter activity in time- and concentration-dependent manner. Hexachlorophene rapidly induced hepatic differentiation of human MSCs judging from expression of liver-specific genes and proteins, PAS staining, and urea production. The effect of orthotopic transplantation of human mesenchymal stem cell-engineered hepatic cell sheets against acute liver injury was examined in one-layered to three-layered cell sheets system. Transplantation of human mesenchymal stem cell-engineered hepatic cell sheets enhanced liver regeneration and suppressed liver injury. The survival rates of the mice were significantly improved. High expression of complement C3 and its downstream signals including C5a, NF-κB, and IL-6/STAT-3 pathway was observed in hepatic cell sheets-grafted tissues. Expression of phosphorylated EGFR and thioredoxin is enhanced, resulting in reduction of oxidative stress. These findings suggest that orthotopic transplantation of hepatic cell sheets manufactured from MSCs accelerates liver regeneration through complement C3, EGFR and thioredoxin.

The liver has a regenerative capacity in response to acute liver injury, however, severe liver damage sometimes threatens life, and in these cases liver transplantation is required. Orthotopic liver transplantation (OLT) is the appropriate therapy for liver failure, but harbors the problems of organ shortage and complications associated with rejection and immunosuppression^1^,^2^. Cell therapy has a potential of alternative therapy to OLT^3^, and various types of cells including mesenchymal stem cells (MSCs) are studied to be applied as cell therapy for liver failure^4^,^5^,^6^,^7^,^8^,^9^,^10^. Humoral factors from MSCs as well as transplantation of MSCs ameliorated acute and chronic liver failure^2^,^8^,^11^,^12^,^13^. MSCs are an optimal cell source for cell therapy in the clinical settings.

We previously reported that Wnt/β-catenin signaling was suppressed during hepatic differentiation process of human MSCs^14^,^15^. In addition, knockdown of signaling molecules or target genes of Wnt/β-catenin signals resulted in hepatic differentiation of human MSCs. Bone marrow-derived MSCs (BM-MSCs) were able to differentiate into hepatocytes in the presence of Dkk-1^16^. Taken together, suppression of Wnt/β-catenin signal plays an important role in hepatic differentiation of MSCs. In the present study, we identified a small molecule compound that efficiently induces hepatic differentiation of human MSCs, since the use of small molecule compounds is a safe way, providing an advantage over using cytokines, nucleic acids or protein drug products^17^.

We manufactured hepatic cell sheets derived from MSCs for treatment of liver failure because cell sheet engineering enabled tissues to retain hepatic functions compared to isolated cell transplantation^18^. This technology enabled us to manufacture the two- and three dimensional functional cell sheets and transplant into the desired sites of the body by minimum invasive procedure^19^. We examined the therapeutic effects of hepatic cell sheets for acute liver injury in mice.

## Results

### Identification of inhibitors of Wnt/β-catenin signaling of MSCs

**Figure 1:**
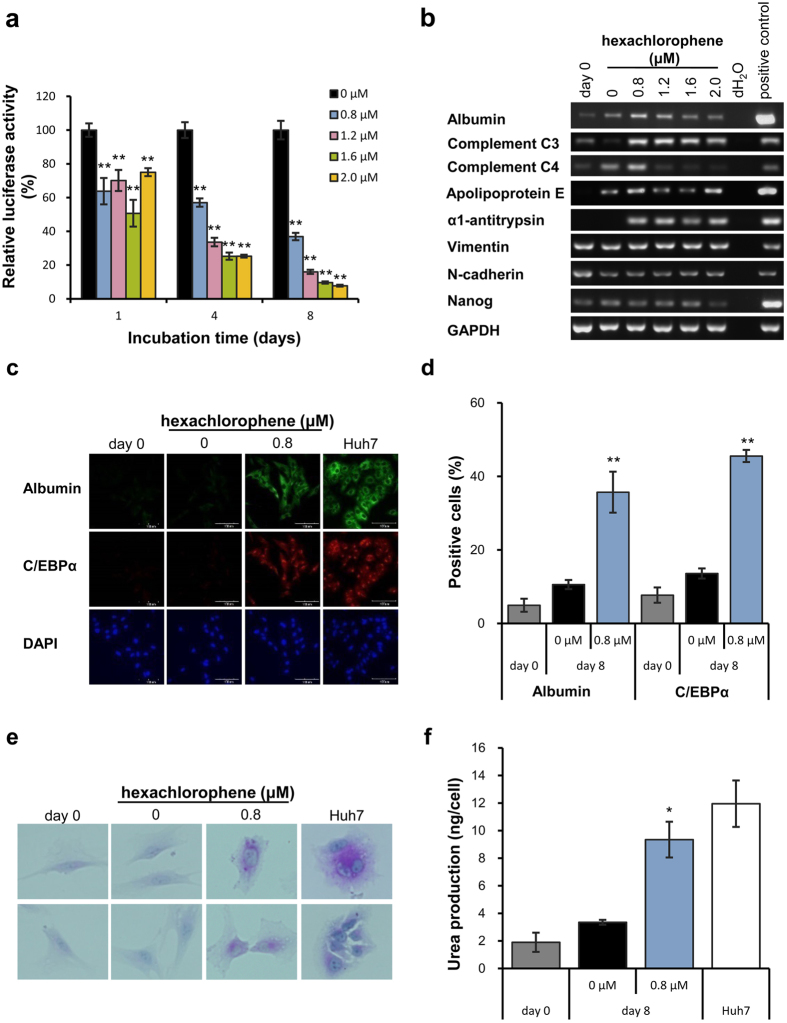
Suppression of Wnt/β-catenin signaling with hexachlorophene induced hepatic specification of MSCs. (**a**) TCF4/β-catenin reporter assay was performed on day 1, 4, and 8 after addition of hexachlorophene. Data are expressed as the mean ± SE of 8 separate wells. *p < 0.05, **p < 0.01, compared to vehicle examined by student’s t-test. (**b**) Effect of hexachlorophene on gene expression was determined on day 8 by RT-PCR analysis. (**c**) Expression of albumin and C/EBPα protein was evaluated by immunofluorescence. Huh7 cells were used as a positive control. (**d**) The percentages of albumin- and C/EBPα- positive cells were evaluated by immunofluorescence. Data are expressed as the mean ± SE of 5 randomly selected fields. *p < 0.05, **p < 0.01, compared to vehicle examined by student’s t-test. (**e**) PAS staining was performed on day 0 and day 8. (**f**) Urea production was determined by urea assay on day 8. *p < 0.05, **p < 0.01, compared to vehicle, respectively, examined by student’s t-test.

We previously reported that suppression of Wnt/β-catenin signal by siRNA enhanced hepatic differentiation of human bone marrow-derived MSCs and umbilical cord-derived MSCs^14^,^15^. In the present study, we focused on ten small molecule compounds including CGP049090, PKF115-584, PKF118-310, PNU-74654, ICG-001, NSC668036, quercetin, ionomycin, imatinib, and hexachlorophene^20^,^21^,^22^,^23^, most of which inhibited Wnt/β-catenin signal in colon cancer cells. To assess the effect of Wnt/β-catenin signal, we carried out reporter assay using the E7-TCF4 cells, which are the UE7T-13 cells stably expressed firefly luciferase gene under the control of the TCF-4 motif. Nine compounds except for NSC668036 inhibited Wnt/β-catenin transcription activities (Supplementary Fig. 1). Of these, hexachlorophene most potently suppressed TCF4/β-catenin transcriptional activity in a time- and concentration-dependent manner (Fig. 1a). Hexachlorophene at 0.8–1.6 μM had little effect on cell viabilities (Supplementary Fig. 2). Hexachlorophene also exhibited suppressive effects on TCF4/β-catenin transcriptional activity in a concentration-dependent manner of human bone marrow mononuclear cells obtained from a patient with osteoarthritis under informed consent (Supplementary Fig. 3).

### Effect of hexachlorophene on hepatic differentiation of human MSCs

To investigate the effect on hepatic differentiation, albumin mRNA was examined after treatment with 9 each compound in UE7T-13 cells. Hexachlorophene potently induced albumin mRNA, but the other compounds did not (data not shown). Hexachlorophene enhanced expression of liver-specific genes including albumin, complement C3, C4, apolipoprotein E, and α1-antitrypsin, and decreased expression of stem cell markers including nanog and N-cadherin except for vimentin (Fig. 1b). Inducible effects of albumin, C4, and apolipoprotein E were also observed by 0.1% DMSO treatment. The effect of DMSO has been reported to induce hepatic differentiation of MSCs^24^, however, treatment with 0.8 μM hexachlorophene induced hepatic gene expression more potently than DMSO treatment. Complement C3 was prominently induced by 0.8 μM hexachlorophene treatment. Hexachlorophene-treated cells expressed hepatocyte-specific proteins including albumin and C/EBPα on day 8 (Fig. 1c). Around 40% of the cells treated with 0.8 μM hexachlorophene expressed albumin and C/EBPα on day 8, respectively (Fig. 1d). Correspondingly, characteristic glycogen granules were observed in cytoplasm of the cells on day 8 (Fig. 1e). Urea production was significantly increased in hexachlorophene-treated cells on day 8, which is comparable to that of Huh7 cells (Fig. 1f). Although liver functions of Huh7 cells are not so high as primary hepatocytes, it was clearly shown that hexachlorophene treatment certainly committed MSCs toward hepatic lineage. Besides UE7T-13 cells, the CD90+CD271+ primary human bone marrow mononuclear cells obtained from a osteoarthritis patient exhibited liver function such as glycogen synthesis and urea production after treatment with hexachlorophene for 8 days (Supplementary Fig. 4). These findings suggest that hexachlorophene rapidly induces hepatic differentiation of of human BM-MSCs.

Since hexachlorophene promoted β-catenin degradation via proteasome^25^, the effect of hexachlorophene on UE7T-13 cells was examined. Whereas treatment with hexachlorophene led to reduction in β-catenin and cyclin D1, the effect of hexachlorophene was abolished by MG-132, a proteasome inhibitor (Supplementary Fig. 5). These data indicate that hexachlorophene inhibits Wnt/β-catenin signaling via degradation of intracellular β-catenin in a proteasome-dependent fashion.

### Hexachlorophene-induced hepatic cell sheets ameliorated acute liver injury in mice

**Figure 2:**
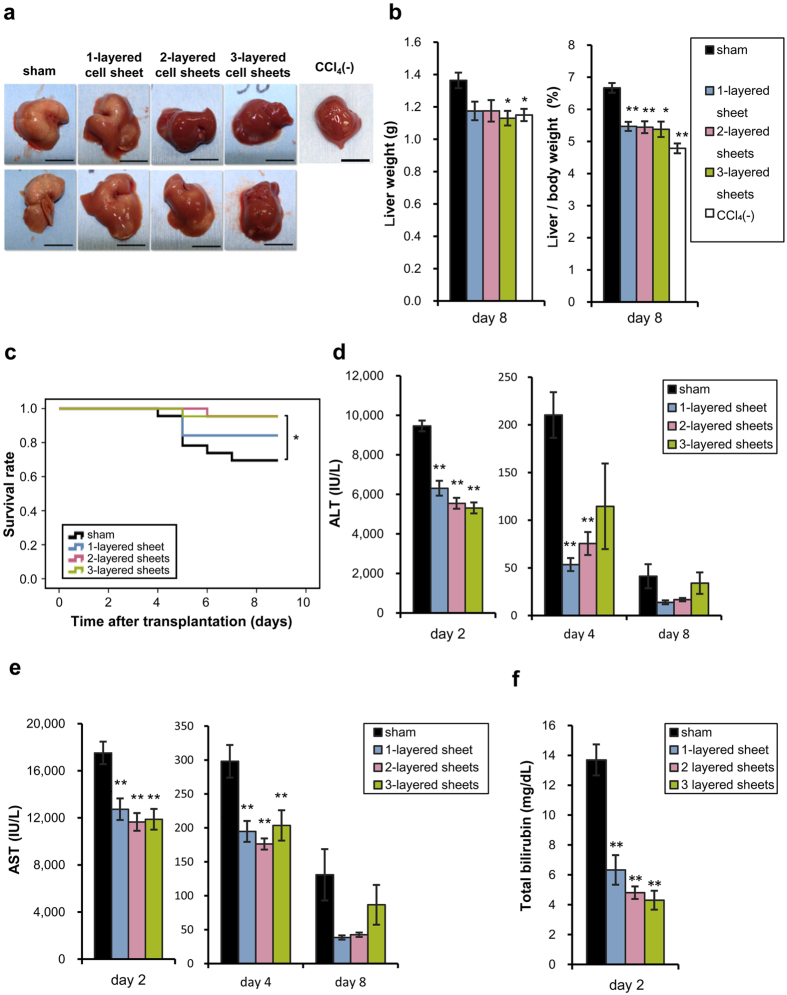
Transplantation of hepatic cell sheets ameliorated acute liver injury. (**a**) Gross appearances of livers from sham-operated and cell sheets-transplanted mice on day 8. CCl_4_(−) represents normal mouse liver without carbon tetrachloride intoxication. Scale bar = 10 mm. (**b**) Liver weight (left panel) and liver/body weight (right panel) on day 8 after transplantation. Data are expressed as mean ± SE (n = 8–10 each except n = 3 for CCl_4_(−)). *p < 0.05, **p < 0.01, compared to sham-operated mice examined by Games-Howell test. (**c**) Survival rate after transplantation (n = 18–20 in each group). *p < 0.05, compared to sham-operated mice and examined by Log-rank test. (**d**) Serum ALT on day 2, 4, and 8 after transplantation. (**e**) Serum AST on day 2, 4, and 8 after transplantation. (**f**) Serum bilirubin on day 2. Data are expressed as mean ± SE (n = 8–10 on each day). *p < 0.05, **p < 0.01, compared to sham-operated mice examined by Games-Howell test.

To clarify the therapeutic effect of hepatic cell sheets, transplantable cell sheets were manufactured by treating UE7T-13 BM-MSCs with hexachlorophene on thermo-responsive polymer coated culture dishes. Although the utility of hepatic tissue sheets has been shown by heterotopic transplantation^18^, orthotopic transplantation was performed to obtain direct effects of hepatic cell sheets. The cell sheets were transplanted onto two sites of the left lateral liver lobe, in 1-layered, 2-layered, and 3-layered dimensional fashion. At 24 hours after transplantation, mice were received carbon tetrachloride by oral gavage. Although normal murine liver without carbon tetrachloride intoxication showed a smooth and soft surface, sham-operated mice livers with carbon tetrachloride intoxication exhibited white multifocal granular accumulations on day 8 (Fig. 2a). However, white multifocal granular accumulations improved in cell sheets number-dependent fashion. All the transplanted groups showed acceleration of body weight recovery from the reduction caused by CCl_4_ (Supplementary Fig. 6). Indeed, liver/body weight on day 8 was lower in transplanted mice than in sham-operated group (Fig. 2b). The survival rates of the mice, which received 2- and 3-layered cell sheets, were significantly improved compared to sham-operated mice (Fig. 2c). On day 2 after transplantation, serum alanine aminotransferase (ALT) was improved in transplanted groups compared to sham-operated group in cell sheets number-dependent manner. Serum aspartate aminotransferase (AST) was also decreased in the transplanted groups (Fig. 2d,e). All the transplanted groups showed marked reduction in serum bilirubin on day 2 in cell sheets number-dependent fashion (Fig. 2f). These data suggest that transplantation of hepatic cell sheets has preventive effects on acute liver injury induced by CCl_4_ administration.

**Figure 3:**
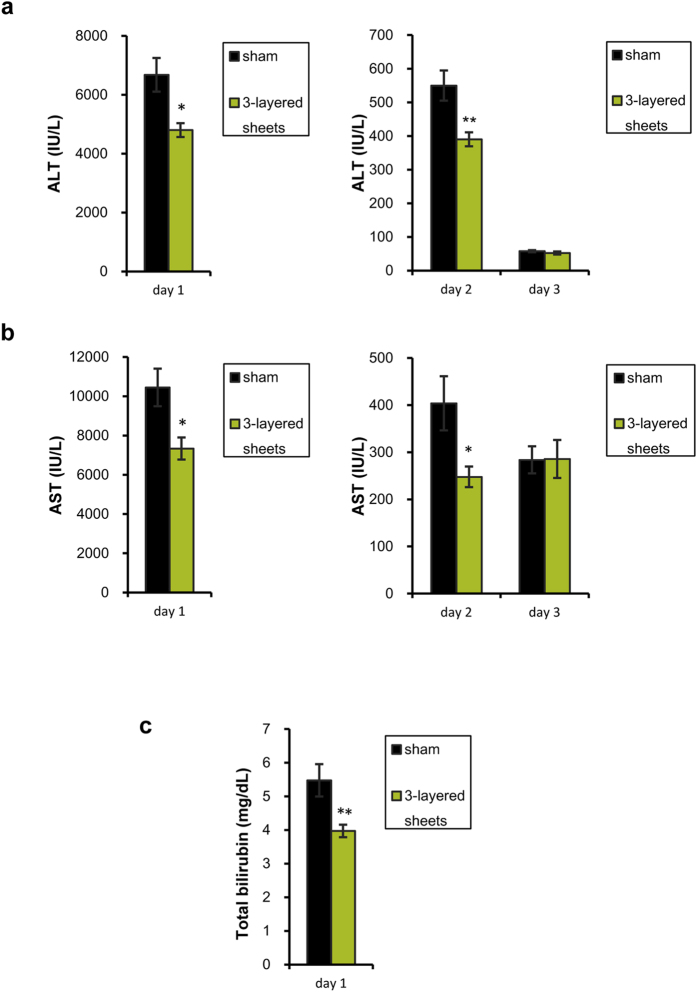
Biochemical tests of initial CCl_4_ liver injury predated hepatic cell sheets transplantation. At six hours after CCl_4_ administration, mice were received hepatic cell sheets onto the livers. (**a**) Serum ALT on day 1, 2, and 3 after CCl_4_ administration. (**b**) Serum AST on day 1, 2, and 3 after CCl_4_ administration. (**c**) Total bilirubin on day 1 after CCl_4_ administration. Data are expressed as mean ± SE (n = 9–10 on each day). *p < 0.05, **p < 0.01, compared with sham-operated mice (Mann-Whitney U-test).

In addition, the therapeutic effects of hepatic cell sheets on acute liver injury were examined in the experiments where hepatic cells sheets were transplanted onto mouse liver at 6 h after CCl_4_ administration (Fig. 3). Serum ALT and AST were significantly lower in the 3-layered cell sheets-transplanted mice than sham-operated mice on 1 and 2 days after CCl_4_ treatment (Fig. 3a,b). Total bilirubin was also decreased in the 3-layered cell sheets-transplanted mice in comparison with the sham-operated mice on day 1 (Fig. 3c). These data suggest that human mesenchymal stem cell-engineered hepatic cell sheets have therapeutic effects on acute liver injury as well as preventive effects.

**Figure 4:**
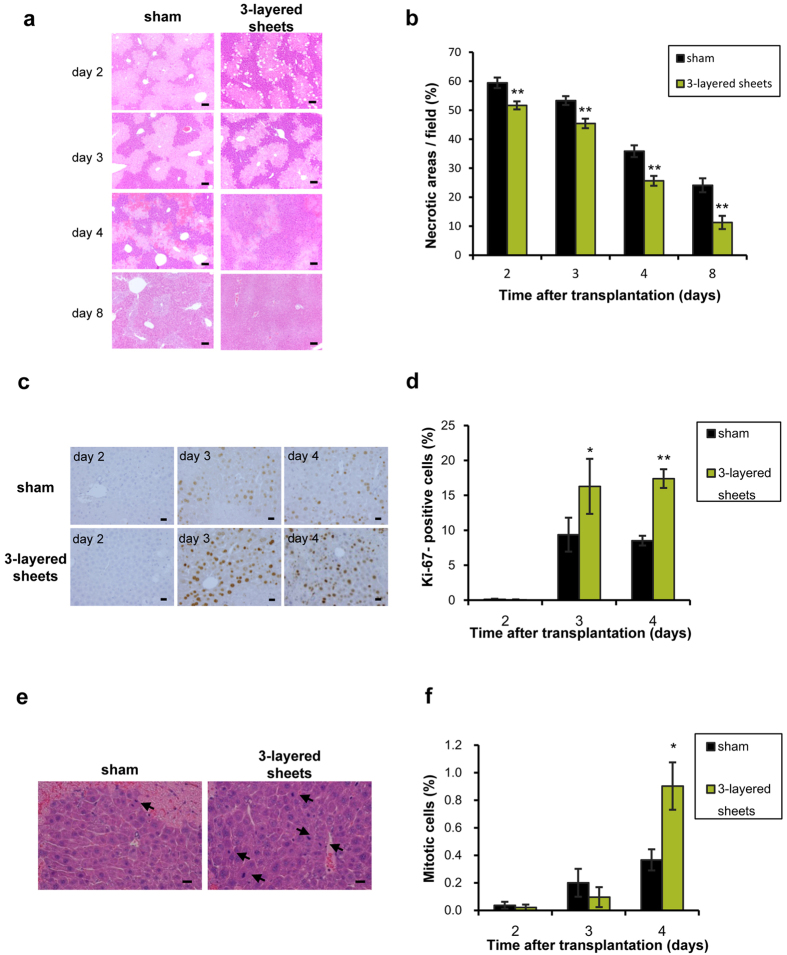
Hepatic cell sheets accelerated liver regeneration. (**a**) Hematoxylin and eosin staining of native livers of sham-operated and 3-layered cell sheets transplanted mice on day 3, 4, and 8. Scale bar = 20 μm. (**b**) Gradual decreases of necrotic areas. Data are expressed as mean ± SE. 5 fields of each individual mice were measured (n = 5–6). *p < 0.05, **p < 0.01, examined by Mann-Whitney U-test. (**c**) The Ki-67-positive hepatocytes of native livers determined by immunohistochemistry. The brown-stained nuclei indicate the positivity for Ki-67. Scale bar = 20 μm. Sham and 3-layered sheets represent liver of sham-operated mice and 3-layered hepatic cell sheets-transplanted liver, respectively. (**d**) The bars show the percentages of Ki-67-positive hepatocytes. Data are expressed as mean ± SE (n = 6, 5 fields of individual mice were measured). *p < 0.05, **p < 0.01, examined by Mann-Whitney U-test. (**e**) Mitotic cells of native livers are shown as arrows on day 4. Scale bar = 20 μm. (**f**) The bars show the mitotic cells which are expressed as mean ± SE (n = 6). 5 fields per individual mice were measured. *P < 0.05, examined by Mann-Whitney U-test.

We examined the details of liver injury in the model where transplantation of hepatic cell sheets was performed and then CCl_4_ was administered on the next day as the follows. Hepatic necrosis diffusely occurred in sham-operated mice on day 2, and was gradually reduced up to day 8 (Fig. 4a). However, necrotic areas in liver of 3-layered cell sheets-transplanted mice showed remarkable recovery on day 2, 3, 4, and 8, compared to sham-operated mice (Fig. 4b). These data indicate that transplantation of hepatic cell sheets potently ameliorates acute liver injury.

### Hepatic cell sheets promoted liver regeneration

To clarify the mechanisms of ameliorating liver injury by hepatic cell sheets, proliferation of hepatocytes was assessed in both 3-layered cell sheets-transplanted and sham-operated livers in the model where transplantation of hepatic cell sheets was performed and then CCl_4_ was administered on the next day. The Ki-67-positive hepatocytes were not observed in the livers of both mice on day 2. However, the Ki-67-positive hepatocytes were dramatically increased in hepatic cell sheets-transplanted mice on day 3 and 4 (Fig. 4c). The positive rates of Ki-67 cells were significantly higher in hepatic cell sheets-transplanted mice than the sham-operated mice (Fig. 4d). The mitosis of hepatocytes actively occurred on day 4. (Fig. 4e). The percentages of mitotic cells in cell sheets-transplanted mice were significantly increased on day 4 in hepatic cell sheets-transplanted mice (Fig. 4f). These data suggest that transplantation of hepatic cell sheets directly promotes liver regeneration via some humoral factors.

**Figure 5:**
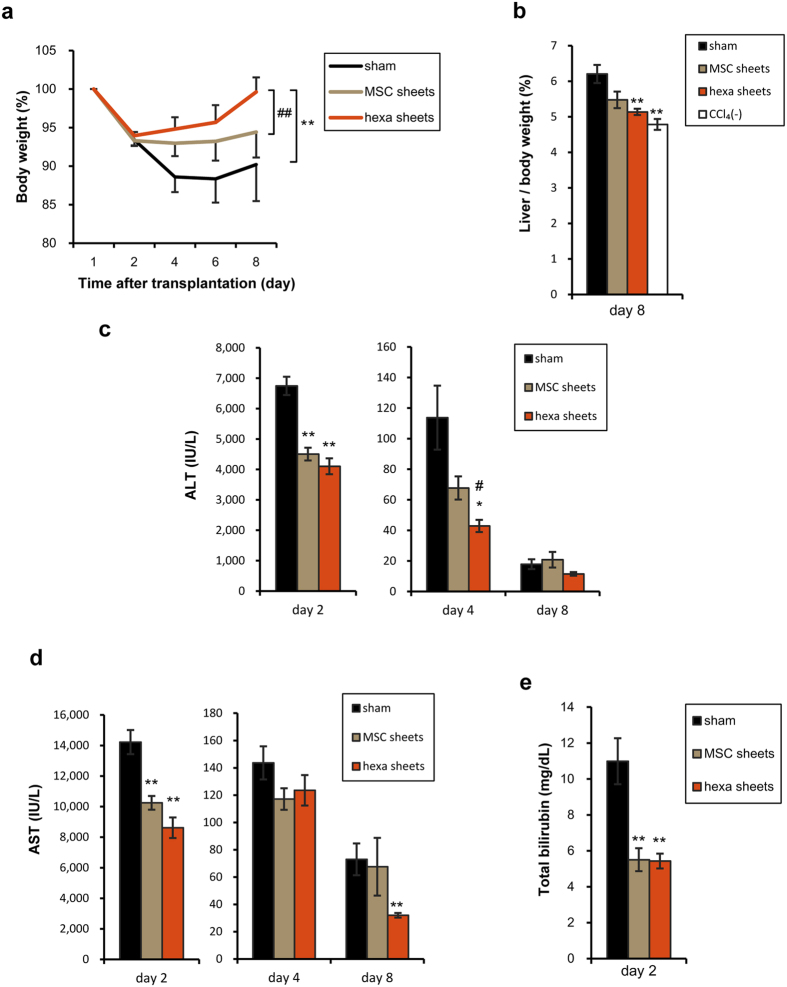
Effects of hepatic differentiated MSCs-derived cell sheets were superior to those of non-differentiated MSCs. (**a**) The changes of body weights of sham-operated, non-induced MSCs transplanted, and hexachlorophene-induced MSCs transplanted mice in acute liver injury model. Data are expressed as mean ± SE (n = 8–10 on each day) of the percentage when that of day 1 is assumed to be 100%. **p < 0.01, compared to sham-operated mice and ^##^*p* < 0.01, compared to non-induced MSC sheets transplanted mice, which were examined by ANOVA for repeated measurements. (**b**) Liver/body weight on day 8 after transplantation are depicted in bars expressed as mean ± SE (n = 7–9 except n = 3 for CCl_4_(−)). **p < 0.01, compared with sham-operated mice (Games-Howell test). (**c**) Serum ALT on day 2, 4, and 8 after transplantation. (**d**) Serum AST on day 2, 4, and 8 after transplantation. Data are expressed as mean ± SE (n = 8–10 on each day). *p < 0.05, **p < 0.01, compared with sham-operated mice and ^#^*p *< 0.05, compared with non-induced MSC sheets transplantation (Games-Howell test). (**e**) Total bilirubin on day 2. Data are expressed as mean ± SE (n = 8–10 on each day). **P < 0.01, compared with sham-operated mice (Games-Howell test).

Since several humoral factors secreted from MSCs improved hepatotoxin-induced hepatic injury^11^,^12^, we clarified whether these therapeutic effects were originated from MSCs *per se* or acquired hepatic functions via hepatic differentiation by hexachlorophene. To clarify this issue, therapeutic effects on liver damages were compared between hexachlorophene-treated cell sheets-transplanted and non-treated BM-MSCs-derived cell sheets-transplanted mice. The recovery of body weight in the hexachlorophene-treated cell sheets-transplanted mice was much faster than in non-treated BM-MSCs-derived cell sheets-transplanted mice (Fig. 5a). Significant reduction in liver/body weight on day 8 was also observed in the hexachlorophene-treated cell sheets-transplanted mice (Fig. 5b). Although serum ALT levels in both groups were lower than sham group on day 2, it was significantly lower in hexachlorophene-treated cell sheets mice than non-treated BM-MSCs sheets mice on day 4 (Fig. 5c). Serum AST was also significantly decreased in both groups, compared to sham-operated group on day 2, but no changes were observed between two groups on day 4. However, on day 8, serum AST was significantly lower in hexachlorophene-treated cell sheets group than sham-operated group (Fig. 5d). Serum bilirubin was lower in both the cell sheets transplanted groups than sham-operated group at day 2, but there was no difference between the groups (Fig. 5e). The survival rate was improved by treatment with hexachlorophene, but not significantly (Supplementary Fig. 7). These data suggest that hepatic differentiation obtained by hexachlorophene provides cell sheets with potent regenerative abilities.

### Complement C3 promotes IL6-STAT3 pathway through NF-κB activation

**Figure 6:**
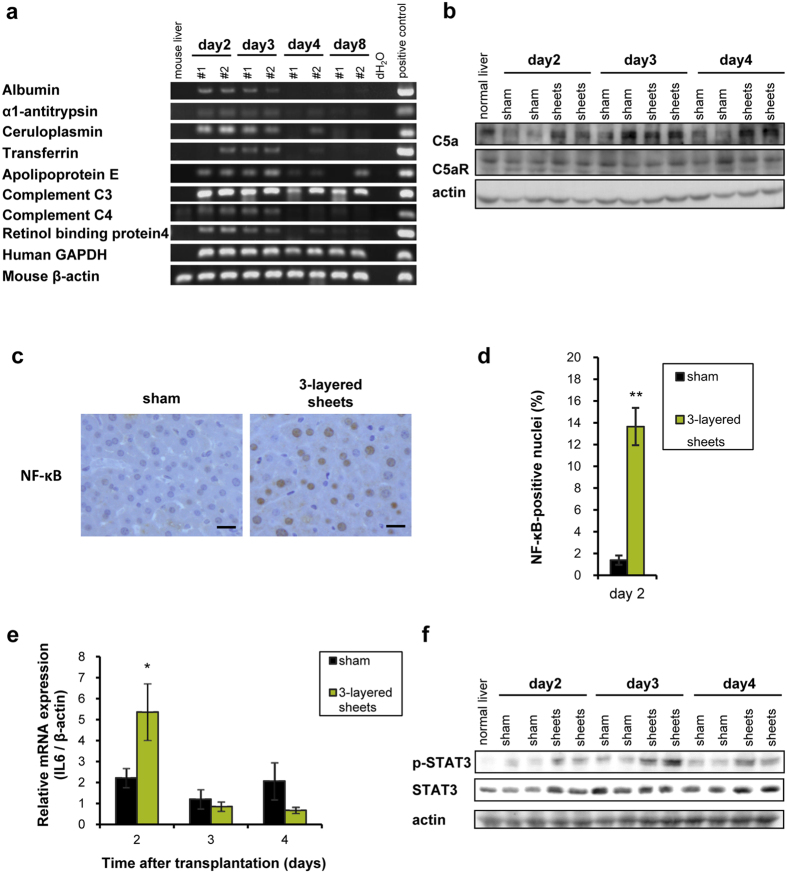
Complement C3 activated IL-6/STAT-3 pathway through C5 followed by NF-κB activation. (**a**) Human-specific gene expression of hepatic origin. Expression of genes in cell sheets was assessed by RT-PCR analysis. All PCR procedures except for mouse β-actin were performed using primers specific for human mRNA transcripts. (**b**) Western blot of native liver tissues excluding grafted cell sheets. C5a was abundant in cell sheets transplanted native liver on day2 and 4. C5aR was not altered between experimental groups. Liver lysates of two mice from each group are used on each day. (**c**) Nuclear localization of NF-κB. NF-κB was detected in hepatocytes of native liver tissues transplanted cell sheets on day 2 by immunohistochemistry. Scale bar = 20 μm. Sham and 3-layered sheets represent sham-operated group without cell sheets transplantation and 3-layered cell sheets derived hexachlorophene-induced MSCs transplanted group, respectively. (**d**) The NF-κB-positive nuclei on day 2 (n = 6, 10 fields per each mice were measured). **p < 0.01 (Mann-Whitney U-test). (**e**) mRNA expression of murine IL-6 in murine liver tissue excluding grafted cell sheets measured by real-time PCR analysis. Bar expressed as mean ± SE (n = 6). *p < 0.05, examined by Mann-Whitney U-test. (**f**) Activation of STAT-3 pathway examined by western blot. Liver lysates without grafted tissues of two mice from each group were used on each day.

To further clarity the cause of liver regeneration, we focused on serum proteins which were secreted by hepatic cell sheets. Expression of various human liver-specific genes including albumin, α1-antitrypsin, apolipoprotein E, and complement C3 and C4 was examined in the grafted tissues (Fig. 6a). Among them, expression of albumin, ceruloplasmin, and apolipoprotein E was decreased with time. The hematoxylin and eosin staining of the grafted cell sheets on day 8 showed that many cells were still stained with eosin, however, the morphologies of many cells were fibroblastic, like MSCs (Supplementary Fig. 8). Taken together, some transdifferentiated cells might dedifferentiate into MSCs. Taking into consideration that hexachlorophene-induced hepatic cell sheets are superior to BM-MSCs sheets, we focused on complement C3, which was intensively expressed from day 2 to 8 (Fig. 6a). Although complement C3 is known to play a role in the immune system, it is also involved in the early priming stages of liver regeneration after partial hepatectomy^26^. Consequently, expression of C5a, a downstream pathway of complement C3, was increased in livers of hepatic cell sheets-transplanted mice on day 2 and 4 (Fig. 6b). Complement C3a and C5a are known to activate NF-κB through binding G-protein coupled receptors C3aR and C5aR on Kupffer cells, respectively^5^. If C5a bound to C5aR on hepatocyte, the priming of hepatocyte in liver regeneration is promoted. On day 2, in accordance with increased expression of C5a, NF-κB was translocated to the nuclei of hepatocytes of 3-layered cell sheets-transplanted livers (Fig. 6c). The positive rates of nuclear NF-κB in hepatocytes of 3-layered cell sheets-transplanted livers were increased by 7-fold (Fig. 6d). IL-6 mRNA, a target gene of NF-κB, was upregulated in cell sheets-transplanted livers on day 2 (Fig. 6e). IL-6 protein was also increased in cell sheets-transplanted livers from day 2 to 4 (Supplementary Fig. 9a). The IL-6-positive hepatocytes were observed in close proximity to grafted cell sheets on day 2 (Supplementary Fig. 9b). IL6 acts as a priming factor of liver regeneration through binding IL-6R/gp130 receptor complex followed by STAT3 activation^27^,^28^. Phosphorylation of gp130 was observed on day 2 and 3 (data not shown), and STAT3 was intensely phosphorylated from day 2 to 4 in transplanted liver (Fig. 6f). Interestingly, the Ki-67-positive hepatocytes were observed beneath the grafted cell sheets on day 2 (Supplementary Fig. 9b), where the IL-6-positive hepatocytes also similarly distributed. These findings are in sharp contrast to those of Fig. 4c, where the Ki-67-positive hepatocytes were not observed in the livers inside that the distance left from the surface on day 2. Taken together, these data suggest that complement C3 secreted from hepatic cell sheets activated IL-6/STAT-3 pathway via C5-mediated nuclear translocation of NF-κB.

### Role of other cytokines secreted from hepatic cell sheets in liver regeneration

**Figure 7:**
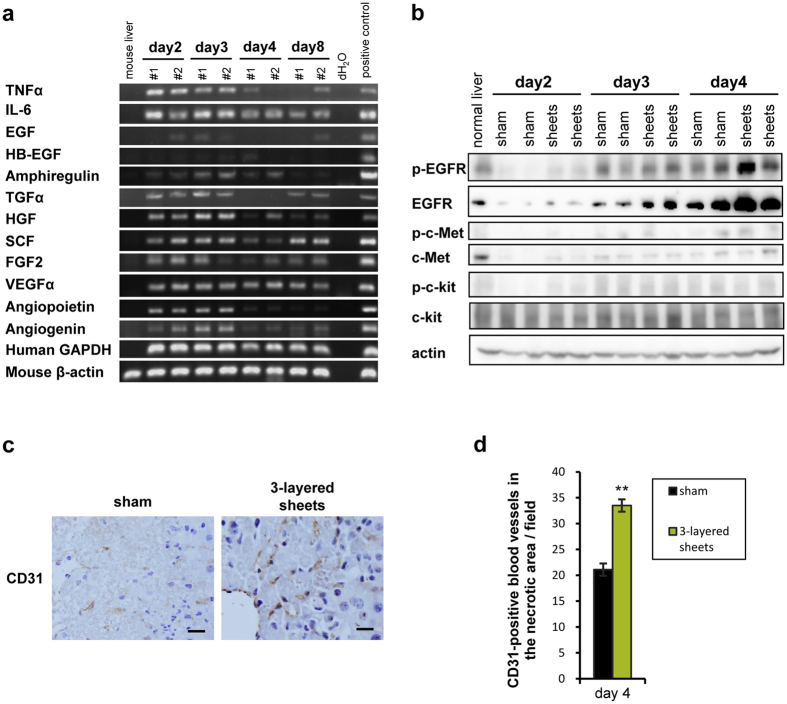
EGFR ligands secreted from grafted cell sheets involved in the phosphorylation of EGFR. (**a**) Human-specific gene expression analysis of cytokines in grafted tissues including cell sheets by RT-PCR analysis. All PCR procedures except for mouse β-actin were performed using primers specific for human mRNA transcripts. (**b**) Downstream signals of cytokines. Western blot was performed by using native liver lysates. (**c**) New vessels in necrotic area of native livers were determined by immunohistochemistry of CD31 on day 4. Sham and 3-layered sheets represent sham-operated group without cell sheets transplantation and 3-layered cell sheets derived hexachlorophene-induced MSCs transplanted group, respectively. (**d**) The number of new vessels in necrotic area was expressed as mean ± SE (n = 6, 10 fields of individual mice were counted). **p < 0.01, examined by Mann-Whitney U-test.

Although hepatic differentiation provides cell sheets with potent regenerative abilities, expression of various cytokines which MSCs originally harbor was also examined in grafted tissues of cell sheets. In the grafted tissues, various human-specific gene expression of MSC-related cytokines such as TNFα, amphiregulin, TGFα, HGF, SCF, FGF2, VEGFα, angiopoietin, and angiogenin was observed (Fig. 7a). To clarify the cytokines involved in this system, downstream signals of these factors in murine liver tissues were examined. Phosphorylation of c-kit as the priming factor of liver regeneration^27^ was slightly detected in hepatic cell sheets-transplanted mice on day 2 (Fig. 7b). HGF and EGF receptor (EGFR) ligands are important growth factors that drive cell cycle progression during liver regeneration^28^. Phospho-c-Met, the receptor of HGF, was not detected, however, the prominent increase in expression and phosphorylation of EGFR in hepatic cell sheets-transplanted liver was observed (Fig. 7b). Of ligands for EGFR, gene expression including TGFα, EGF, HB-EGF, and amphiregulin^29^,^30^, was not altered between experimental groups (Supplementary Fig. 10), suggesting that EGFR signaling is activated by EGFR ligands derived from cell sheets. Human-specific gene expression including TGFα and amphiregulin was observed (Fig. 7a). Although TGFα was expressed equally on day 2 and 3, expression of amphiregulin reached its peak on day 3 (Fig. 7a), suggesting that amphiregulin is a more plausible agonist than TGFα. Since angiogenesis in necrotic area was significantly increased in transplanted tissues on day 4 (Fig. 7c,d), angiogenic factors including EGFα, angiopoietin, and angiogenin may play a role in this process (Fig. 7a). These data suggest that several cytokines play a role in the promotion of liver regeneration.

### Transplantation of hepatic cell sheets reduced oxidative stress

**Figure 8:**
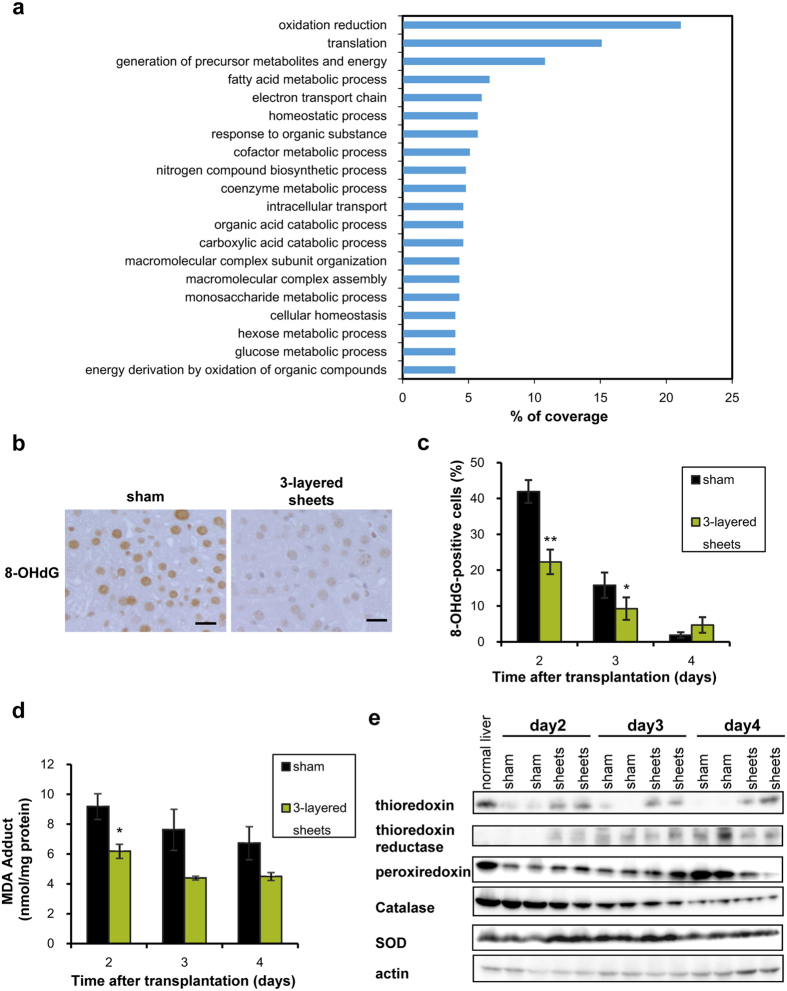
Redox state of cell sheets-transplanted murine liver. (**a**) GO classification. The percentages of coverage representing the percentage of proteins annotated with a specific GO term related to Biological Processes are shown (*p* < 0.001). GO annotation was performed with more than two-fold alterations in protein expression between the experimental groups. (**b**) Immunohistochemical analysis of 8-OHdG in native livers. Scale bar = 20 μm. Sham and 3-layered sheets represent sham-operated group without cell sheets transplantation and 3-layered cell sheets derived hexachlorophene-induced BM-MSCs transplanted group, respectively. (**c**) The percentages for 8-OHdG-positive cells were expressed as mean ± SE (n = 6, 5 fields of individual mice were counted). *p < 0.05, **p < 0.01, examined by Mann-Whitney U-test. (**d**) MDA adducts of murine liver tissues excluding grafted cell sheets. MDA adducts were measured by MDA ELISA analysis. The columns were expressed as mean ± SE (n = 3). *p < 0.05, examined by Mann-Whitney U-test. (**e**) Expression of proteins concerning thioredoxin reduction and oxidation cycle and catalase and SOD in Western blotting. Native liver lysates excluding grafted cell sheets were analyzed.

**Figure 9:**
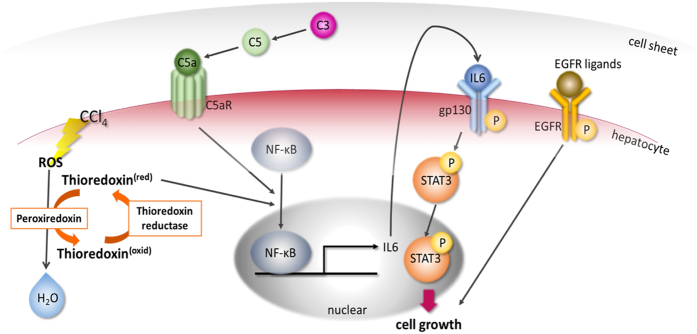
Proposed mechanisms of liver regeneration in the hepatic cell sheets transplantation system. Complement C3 derived from human hepatic cell sheets activates NF-κB via complement C5 resulted in nuclear translocation of NF-κB of hepatocytes. Thioredoxin also activates NF-κB through deoxidizing NF-κB, by which in turn thioredoxin is oxidized. Thioredoxin is involved in redox system accompanied by thioredoxin reductase and peroxiredoxin. Nuclear localized NF-κB upregulates transcriptional activity of IL-6, resulting in activation of IL6/STAT3 pathway and allowing hepatocytes to enter cell cycle. Activation of EGFR pathway is also involved in promoting cell growth.

To comprehensively explore further effects of cell sheets transplantation, proteomics analysis using mass spectrometry was performed. Protein expression was compared between 3-layered cell sheets transplanted and sham-operated livers. Expression of 883 proteins was different between two groups by at least one-pair of comparative analysis (Supplementary Table 1). Ontological analysis revealed that oxidation reduction was significantly picked up by variations of 74 protein expression (Fig. 8a and Supplementary Table 2). Most of them were declined in transplanted livers on day 2, however, redox proteins including thioredoxin, peroxiredoxin, and glutaredoxin^31^, exhibited higher expression in transplanted livers than sham-operated livers (Supplementary Table 2). Hence, dynamically altered protein expression involved in redox state was examined. 8-OHdG was intensely stained in sham-operated mice livers, however, it was significantly reduced in transplanted mice at day 2 and 3 (Fig. 8b,c). MDA adducts were also significantly reduced in transplanted mice compared to sham-operated mice on day 2 (Fig. 8d). The reduction of thioredoxin and peroxiredoxin, which was induced by liver injury, was interrupted by transplantation of cell sheets on day 2 and 3. Thioredoxin reductase was also upregulated in cell sheets-transplanted mice on day 2 (Fig. 8e). These data suggest that activation of thioredoxin oxidation and reduction cycle contributed to redox state in transplanted mice. Although SOD and catalase have been reported to affect the redox state after transplantation of MSCs^2^,^32^,^33^, decreased catalase and increased SOD was observed in transplantated mice on day 2 (Fig. 8e). Although glutathione peroxidase and glutathione reductase were not altered between experimental groups, however, GSSG/GSH was higher in transplanted group on day 2 (Supplementary Fig.11a,b). This may be partly because excess GSSG led to activation glutaredoxin^31^. These data suggest that thioredoxin oxidation and reduction cycle plays a key role in antioxidative effect of cell sheet therapy^34^. The other antioxidative proteins such as metallothionein^35^, fatty acid binding protein^36^, and apoptosis inducing factor 1^37^ were also picked up by proteomics analysis. Taken together, these data suggest that the mechanisms of liver regeneration in the hepatic cell sheets transplantation system is proposed in Fig. 9.

## Discussion

Several studies reported that MSCs were transdifferentiated into hepatocytes^6^,^10^,^15^,^16^, and intrasplenic or intravenous injection of hepatic differentiated MSCs ameliorated liver injury in mice^2^,^8^. MSCs are have beneficial effects in treatment of acute liver failure and cirrhosis^13^. However, transplantation of MSCs through portal vein is far from safe because of the risk of thromboembolism. In the present study, we developed a novel therapy system, in which cell sheets generated from MSCs by temperature-responsive polymer-coated dishes in the presence of a Wnt/β-catenin signal inhibitor were transplanted onto the surface of liver. This procedure dramatically ameliorated liver injury in cell sheet number-dependent manner. Survival rate of the mice receiving this therapy was significantly better than that of sham-operated mice. The present study showed that complement C3 is important for accelerating liver regeneration as well as antioxidative properties which MSCs originally harbor^2^,^30^,^32^.

Since it is well known that the mice which lack CYP2E1 expression are resistant to CCl_4_ since CYP2E1 is responsible for bioactivation of CCl_4_^38^. CYP2E1 expression was examined by using the mouse liver on 1 day after transplantation of cell sheets without CCl_4_ intoxication (designated 0 h in Supplementary Fig. 12a) and at 12 h after administration of CCl_4_ (Supplementary Fig. 12a). CYP2E1 expression was not different between sham-operated and cell sheets transplanted mice at 0 h and 12 h, suggesting that CYP2E1 expression which is responsible for bioactivation of CCl_4_, is not influenced by transplantation of cell sheets. As a result, there were no changes in ALT and AST at 0 h and 12 h after transplantation (Supplementary Fig. 12b,c).

Strategies of hepatic differentiation of MSCs have been limited to treatment with cytokines^6^,^10^,^15^,^16^. Since small molecule compounds are more safe and stable than nucleic acids and protein products, they are easily applicable for clinical settings^39^. This procedure highlighted a single compound instead of various cytokines. Although the reason why suppression of Wnt/β-catenin pathway induces hepatic differentiation of MSCs remains to be clarified, a strong hint comes from the fact that β-catenin expression must be suppressed during the competence and specification stage in order for normal liver development to occur^40^,^41^. Wnt/β-catenin signaling has been demonstrated to maintain stemness^42^. Endodermal differentiation is induced by high level of activin/Nodal signals. An endoderm progenitor including hepatic population was generated by culturing ES cells with activin, BMP-4, and FGF-4^43^. Inhibition of Wnt/β-catenin signaling suppresses expression of BAMBI, BMP and activin membrane-bound inhibitor^44^. From these findings, suppression of Wnt/β-catenin signaling is associated with induction of activin/Nodal signaling caused by inhibition of BAMBI.

In the present study, orthotopic transplantation of hepatic cell sheets accelerated liver regeneration in mice. These effects were superior to transplantation of non-differentiated MSC cell sheets. In the grafted tissues, complement C3/C5a/C5aR pathway was activated followed by nuclear translocation of NF-κB of hepatocytes, resulting in enhanced IL-6 expression in hepatocytes. Interestingly, IL-6 expression was enhanced especially beneath the cell sheets, similarly to observed Ki-67-positive hepatocytes on day 2. C3, C5 and IL-6 were involved in promoting cell cycle from G0 to G1^27^,^45^. Further progression of cell cycle could be partly promoted by EGFR phosphorylation through cell sheets-derived EGFR ligands. Another possible contribution to the liver regeneration could be antioxidant properties of the cell sheets. Transplantation of hepatic cell sheets reduced reactive oxygen species (ROS) of recipient livers. Thioredoxin has another important molecular function, which regulates activity of a series of transcription factors including NF-κB^31^,^46^. Prominent NF-κB nuclear localization on 2 days was probably regulated by thioredoxin. Moreover, in Figs 6b,f, 7b and 8e, the western blot analysis of C5a, C5aR, STAT3, EGFR, and oxidative stress-related proteins was performed by using the same liver lobe of the grafted cell sheets. However, overexpression and/or activation of these proteins was not observed when the different lobe from the grafted lobe of cell sheets was used as the sample of western blotting. These data suggest that the effects of hepatic cell sheets transplantation are associated with the distance from transplanted cell sheets.

We identified hexachlorophene as a Wnt/β-catenin signal inhibitor for human BM-MSC UE7T-13 cells. Hexachlorophene was used as an antiseptic and was reported to be toxic^47^. However, our strategy to use hexachlorophene as an inducer of hepatic differentiation of cell sheets is safe due to the following reasons. First, 3% (73.7 mM) hexachlorophene was used as an antiseptic. We used 0.8 μM hexachlorophene, which is 92,125-fold lower than the concentration used as an antiseptic. Indeed, 0.8 μM hexachlorophene has little effect on cell viability, as shown by WST assay (Supplementary Fig. 2). Second, hexachlorophene was thoroughly removed by washing twice with hexachlorophene-free medium. Therefore, hexachlorophene should be undetectable in cell sheets, and cell sheets should be absolutely safe since they contain little hexachlorophene. However, further efforts to identify more effective and safer compounds are required to open a way to clinical application.

In the present study, we demonstrated that human mesenchymal stem cell-engineered hepatic cell sheets have therapeutic effects on acute liver injury as well as preventive effects (Fig. 3). In addition, we found that human bone marrow cells from a patient with osteoarthritis gained liver functions such as glycogen synthesis and urea production by treatment of hexachlorophene for 8 days (Supplementary Fig. 4). These data suggest that human BM-MSC-engineered hepatic cell sheets can be applicable for acute liver diseases in clinical settings. In addition, a novel approach in cell sheets technology using hepatocytes and fibroblasts has been reported to be useful as therapy of liver injury^48^,^49^. Since these cell sheets technologies have advantages of rapid fabrication and high vascularization, in combination with these excellent technologies, human BM-MSC-engineered hepatic cell sheets hopefully will become promising therapy for liver disease.

In conclusion, we developed a new therapy system for acute liver injury as an alternative therapy to liver transplantation, in which hepatic cell sheets derived from BM-MSCs served as cell therapy.

## Methods

### Chemicals and cells

Hexachlorophene, quercetin, CGP049090 and PKF115-584 were obtained from Sigma-Aldrich (St. Louis, MO). Ionomycin was obtained from Calbiochem (La Jolla, CA). Imatinib was purchased from Novartis International AG (Basel, Switzerland). PNU-74654, ICG-001, PKF118-310, and NSC668036 were synthesized in house. For all experiments, compounds were fleshly prepared by being dissolved in dimethyl sulfoxide (DMSO) or dH_2_O shortly prior to the experiment. The final concentration of DMSO in each experiment was 0.1%. UE7T-13 cells, human bone-marrow derived mesenchymal stem cell line^50^, were used. Maintenance and differentiation medium was used DMEM (NICHIREI BIOSCIENCES INC., Tokyo, Japan) containing 10% fetal bovine serum (Nichirei), 100 U/ml penicillin, and 100 μg/ml streptomycin.

### Cell viability assay

At 24 h after seeding of UE7T-13 cells at density of 9.0 × 10^3^ cells/cm^2^, various concentrations of compounds were added. Cell viability was assessed at 0, 2, 4, and 8 days with the use of cell counting kit (DOJINDO LABORATORIES. Kumamoto, Japan).

### Establishment of TCF-4 motif-responsive luciferase stable cell line in UE7T-13 cells

The construct which contains three copies of the optimal TCF-4 motif CCTTTGATC upstream of the cytomegalovirus (CMV) promoter was inserted into multiple cloning sites of pGL4.20 firefly luciferase reporter plasmid (Promega Corp., Fitchburg, WI), and was designated pTCF4-CMVpro-GL4.20. UE7T-13 cells were stably transfected with the linearized pTCF4-CMVpro-GL4.20 by electroporation and the puromycin-resistant clone was designated E7-TCF4.

### Reporter assay

At 24 h after seeding of E7-TCF4 cells at density of 9.0 × 10^3^ cells/cm^2^, various concentrations of compounds were added. At 1, 4, and 8 days after addition, reporter assay was performed with Luciferase assay system (Promega Corp.) using plate reader (PerkinElmer, Inc., MA).

### *In vitro* hepatic differentiation procedure of MSCs

UE7T-13 cells seeded at a density of 9.0 × 10^3^ cells/cm^2^ were cultured overnight to allow cell attachment. After the addition of each compound, hepatic differentiation was performed for 8 days. Medium was changed on day5.

### RNA extraction and reverse transcription- polymerase chain reaction (RT-PCR)

Total RNA from the cells was extracted with TRIzol reagent (Life Technologies Corp., Carlsbad, CA) and subjected to reverse transcription using Superscript II (Life Technologies Corp.) and oligo(dT)_18_ primers for *in vitro* assay and random hexamer for *in vivo* assay. RT-PCR was performed using gene specific primers and rTaq DNA polymerase (TOYOBO CO., Ltd. Osaka, Japan). Primer lists for RT-PCR analysis are indicated in Supplementary Tables 3–6.

### Immunofluorescence analysis

At 8 days after incubation with compounds, the cells were fixed and performed immunofluorescence analysis as described previously^51^. Antibodies used in this study were as follows: anti-human serum albumin antibody (Sigma-Aldrich Corp.), and anti-human C/EBPα antibody (Santa Cruz Biotechnology, Inc., Santa Cruz, CA), and AlexaFluoro 594 goat anti-rabbit immunoglobulin G or AlexaFluoro 488 goat anti-mouse immunoglobulin G (Molecular Probes, Leiden, Netherlands). Nuclei were stained with 4′,6-diamidino-2-phenylindole (Sigma-Aldrich Corp.).

### Periodic acid-Schiff staining for glycogen

UE7T-13 cells treated with the indicated compound for 8 days were trypsinized and inoculated onto cover glasses. The cells were fixed in phosphate-buffered saline containing 4% paraformaldehyde. Periodic acid-Schiff (PAS) staining was performed as described previously^14^,^15^.

### Urea Assay

At 8 days after cultivation with compounds, UE7T-13 cells were incubated in the media with 5 mM ammonium chloride, and the amount of urea secreted into the medium was measured according to the previous reports^14^,^15^.

### Western blot analysis of cell lysates

Ten micrograms of the cell lysis proteins were subjected to western blot analysis. The rabbit polyclonal antibodies including anti–β-catenin (Cell Signaling Technology Inc.), mouse monoclonal antibodies including anti-cyclin D1 (Santa Cruz Biotechnology, Inc.), and goat polyclonal antibodies including anti-Actin (Santa Cruz Biotechnology, Inc.) were used. Actin was used as an internal control. The bands were detected by ImageQuant LAS4000 (GE Healthcare UK Ltd, Little Chalfont, UK).

### Hepatic cell sheets by treating mesenchymal stem cells with hexachrolophene

Human UE7T-13 BM-MSCs were plated onto φ60 mm-temperature-responsive culture dishes (CellSeed Inc., Tokyo, Japan) at a cell density of 9.0 × 10^3^ cells/cm^2^ and incubated with 0.8 μM hexachlorophene at a temperature of 37 °C. After incubation for 8 days, the cells were detached from the culture dishes as a cell sheet by incubating at a temperature of 20 °C for about 15 min. The cell sheets of non-differentiated MSCs were created by plating UE7T-13 cells at a cell density of 3.6 × 10^4^ cells/cm^2^ and incubating them without hexachlorophene at a temperature of 37 °C for 4 days.

### Animal experiments

All animal experiments were performed with the approval of the Tottori University Subcommittee on Laboratory Animal Care, and carried out in accordance with the approved guidelines. Eight to nine-weeks-old nonobese diabetic severe combined immunodeficient (NOD-SCID) mice were purchased from CLEA Japan (Tokyo, Japan). The mice had free access to food and water, and were housed under pathogen-free conditions in a temperature-controlled room and illuminated 12 h daily. The cell sheets were transplanted onto two sites of liver surface of each mouse. The additional cell sheets were overlaid one by one on the pre-existing cell sheets. The 1-, 2-, and 3-layered cell sheets transplantation group represent the mice which received 1-, 2-, and 3-layered cell sheets, respectively. Control mice were operated sham laparotomy. The cell sheets of UE7T-13 BM-MSCs without treatment with hexachlorophene were also transplanted onto two sites of left lateral lobe of liver in a manner of 3-layered cell sheets. On 1 day after transplantation, all the groups were administrated 0.2 ml/kg of carbon tetrachloride (Wako Pure Chemical Industries Ltd., Osaka, Japan) dissolved in olive oil at 5% concentration by oral gavage. On 2 and 4 days after transplantation, blood samples were collected from retro-orbital venous plexus under isoflurane (AbbVie Inc. Tokyo, Japan) anesthesia. On 8 days after transplantation, mice were sacrificed by exsanguination under anesthetized with pentobarbital sodium and blood samples were collected from inferior vena cava. Collected blood were kept on ice overnight, and then centrifuged at 2,000 g for 20 min at 4 °C. To examine therapeutic mechanisms, 3-layered cell sheets transplanted mice and sham-operated mice were sacrificed on day 1, day 1.5 in addition to day 2, 3, and 4. Liver tissues were dissected and weighed wet volume.

The therapeutic effects of hepatic cell sheets on acute liver injury was assessed as follows. Eight to nine-weeks-old NOD-SCID mice were administered 0.2 ml/kg of carbon tetrachloride (Wako Pure Chemical Industries Ltd., Osaka, Japan) dissolved in olive oil at 5% concentration. At 6 h after oral administration of CCl_4_, 3-layered cell sheets were transplanted on two sites of the mouse liver surface. The sham-operated mice without transplantation of cell sheets were used as controls. On 1, 2, and 3 days after CCl_4_ administration, the mice were sacrificed and blood samples were collected for measurement of ALT, AST and total bilirubin.

### Biochemical tests

Serum aminotransferases were measured by Transaminase CII-test Wako (Wako Pure Chemical Industries Ltd.) according to the manufacture’s protocol. Serum bilirubin was measured by QuantiChrom™ Bilirubin Assay Kit (BioAssay Systems LLC, Hayward, CA). Both absorbance was measured using plate reader (Tecan Japan Co., Ltd., Kanagawa, Japan).

### Histology

Liver tissues containing the cell sheets were fixed in 4% parafolmaldehyde and paraffin-embedded. 3 μm thick slice was used for H&E staining according to standard methodology. The necrotic area was measured by ImageJ software (http://imagej.nih.gov/ij/).

### Immunohistochemistry

3-μm thick sections were examined using immunohistochemistry. The sections were deparaffinized, and antigens were retrieved by autoclave in citrate buffer or protease for 30 min at 37 °C. Endogenous peroxidase activity was blocked by immersing the slides in 3% hydrogen peroxide in methanol for 15 min except for 8-OHdG staining. The sections were immunostained using a Histofine mouse stain kit (NICHIREI BIOSCIENCES INC.) for mouse monoclonal primary antibodies including anti-8-OHdG (Japan Institute for the Control of Aging, Nikken SEIL CO., Lte., Shizuoka, Japan) and anti-NF-κB (Santa Cruz Biotechnology, Inc., Santa Cruz, CA). Hsitofine Simple Stain™ Mouse MAX PO (Rat) was used for rat primary antibody, anti-Ki-67 (DakoCytomation, Glostrup, Denmark). Histofine Simple Stain™ Mouse MAX PO (G) was used for goat primary antibody, anti-CD31 (BD Biosciences, San Jose, CA, USA). Histofine Simple Stain™ Mouse MAX PO (R) (NICHIREI BIOSCIENCES INC.) for rabbit polyclonal IL-6 antibody (Abcam Ltd., Cambridge, UK). Positive cells for 8-OHdG and Ki-67 were counted automatically by inForm, advanced image analysis software (PerkinElmer, Massachusetts, USA).

### Western blot analysis of liver tissue lysates

Ten to thirty micrograms of the tissue lysis proteins not including grafted cell sheets or including cell sheets were subjected to western blot analysis. Antibodies used in this experiment as follow: anti-C5aR, IL-6, Glutatione peroxidase 1, Glutathione reductase, catalase (Abcam Ltd.), anti-C5a, c-kit, SOD1 (Santa Cruz Biotechnology, Inc.), anti-TRXR1, EGFR, phospho-EGFR, phosphor-c-kit, STAT3, phosphor-Stat3 (Cell Signaling Technology Inc.), anti-peroxiredoxin 2 (Sigma-Aldrich Corp.), and goat polyclonal antibodies including anti-Actin (Santa Cruz Biotechnology, Inc.). Actin was used as an internal control. The bands were detected by ImageQuant LAS4000 (GE Healthcare UK Ltd.).

### Oxidative stress analysis

MDA adduct content was measured by OxiSelect™ MDA Adduct ELISA Kit (Cell Biolabs, Inc., San Diego, CA) according to the manufacture’s instructions. GSSG/GSH was measured by GSSG/GSH Quantification Kit (DOJINDO LABORATORIES, Kumamoto, Japan) according to manufacture’s protocols. Absorbance was measured using plate reader (Tecan Japan Co., Ltd.).

### Quantitative RT-PCR analysis

cDNA was synthesized by reverse transcription using Superscript II (Life Technologies Corp.) and oligo (dT)_18_ primers. Real-time RT-PCR was performed using LightCycler® FastStart DNA Master SYBR Green I (Roche Diagnostics GmbH., Mannheim, Germany). PCR run was performed in LightCycler system (Roche Diagnostics GmbH.). Primers for real-time RT-PCR analysis are as follows: murine IL6-Forward, 5′- acctggagtacatgaagaacaactt -3′; murine IL6-Reverse, 5′-ggaaattggggtaggaagga -3′, mβ-actin-Forward, 5′-agagcaagagaggtatcctg -3′; mβ-actin-Reverse, 5′-agagcatagccctcgtagat -3′.

### Proteome analysis

The tissues were frozen in liquid nitrogen and then disrupted with Multi-beads shocker (MB400U, Yasuikikai). The disrupted cells were suspended with 0.1 M Tris-HCl (pH 9.0), containing protease inhibitors cocktail (SIGMA-Aldrich). The homogenate was centrifuged at 1,500 g for 10 min. The supernatant was collected and urea was added to a final concentration of 8 M. The solution was reduced with dithiothreitol, alkylated with iodoacetamide, and then digested with Lys-C (WAKO) followed by dilution and digestion with sequence grade modified trypsin (Promega). After digestion, the samples were desalted with StageTip^52^ using SDB-XC disk membrane (3 M). The desalted peptides were labeled with differential stable isotopes in samples for relative protein quantification by using dimethyl labeling method^53^. The labeled peptides were desalted as mentioned above and analyzed by nanoLC-MS/MS analysis using LTQ-Orbitrap XL (Thermo Fisher Scientific) in accordance with a previous report^54^. Peptides and proteins were identified by means of automated data base searching using Mascot version 2.3 (Matrix Science, London, UK) against UniProt/SwissProt release 2012_02× with a precursor mass tolerance of 3 ppm, a fragment ion mass tolerance of 0.8 Da, and strict trypsin specificity allowing for up to two missed cleavages. Cysteine carbamidomethylation was set as a fixed modification, and methionine oxidation and [^1^H_4_,^12^C_2_] or [^2^H_4_,^13^C_2_]-dimethylation of *N*-terminal and lysine ɛ-amino groups were allowed as variable modifications. Peptides were considered identified if the Mascot score was over the 95% confidence limit based on the “identity” score of each peptide. Proteins showing altered expression with fold change values of ≥2.0 between the groups were exported for functional annotation to The Database for Annotation, Visualization and Integrated Discovery (DAVID).

### Statistical Analysis

All the values in the present study were expressed as mean ± SE. The significant differences between the groups were analyzed by a Student’s t test or a Mann–Whitney U test and a Games Howell test. The significance of survival was analyzed by a Log-rank test. All statistical analysis was performed using predictive analytics software (SPSS Inc., Chicago, IL, USA). A *P*-value less than 0.05 was considered to be significant.

## Additional Information

**How to cite this article**: Itaba, N. *et al.* Human mesenchymal stem cell-engineered hepatic cell sheets accelerate liver regeneration in mice. *Sci. Rep.* **5**, 16169; doi: 10.1038/srep16169 (2015).

## Supplementary Material

Supplementary Information
