# Supplementary material for: Human mesenchymal stem cell-engineered hepatic cell sheets accelerate liver regeneration in mice
Source: Sci Rep. 2015 Nov 10;5:16169. doi: 10.1038/srep16169 (PMC4639852; doi:10.1038/srep16169)
Supplement: Supplementary Information [file srep16169-s1.pdf]

Supplementary information

Human mesenchymal stem cell-engineered hepatic cell sheets accelerate liver regeneration in mice

Noriko Itaba[1#], Yoshiaki Matsumi[1#], Kaori Okinaka[1], An Afida Ashla[1], Yohei Kono[1], Mitsuhiko Osaki[2], Minoru Morimoto[3], Naoyuki Sugiyama[4], Kazuo Ohashi[5], Teruo Okano[6], Goshi Shiota[1*]

Supplementary materials and methods

Cells

The cells used in Supplementary Fig.1, 2, 5, 6, 7, 8, 9, 10, 11, and 12 were UE7T-13 cells, human bone-marrow derived mesenchymal stem cell line. The cells used in Supplementary Fig.3 and 4 were the bone marrow mono nuclear cells obtained from human female at the operation. This study was approved by the ethics committees of Tottori University (#1611). The patient gave written informed consent for sample collection and analyses described in the present study in agreement with the Declaration of Helsinki. Briefly, on replacement arthroplasty, bone marrow was obtained from the femur head of osteoarthritis of a 60 yr old female patient after predated approval and signing of informed consent. Bone marrow mononuclear cells were separated by standard methods using Ficoll-Paque (GE Healthcare UK Ltd., Little Chalfont, UK). According to the previous report,[1] high colony forming unit population, CD90+CD271+ mesenchymal stem cells were sorted by following methods; bone marrow mononuclear cells were stained with APC mouse anti-human CD90 antibody (BD Biosciences, San Jose, CA, USA) and PE mouse anti-human CD271 antibodies (Miltenyi Biotec GmbH, Bergisch Gladbach, Germany) for 30 min on ice and CD90+CD271+ cells were sorted by MoFlo XDP (Beckman Coulter Inc. Fullerton, CA, USA). Cells were cultured and

expanded in DMEM (Life Technologies Corp., Carlsbad, CA) DMEM (Life Technologies Corp., Carlsbad, CA) containing 20% fetal bovine serum (FBS; GE Healthcare UK Ltd, Little Chalfont, UK), 20 ng/ml of basic FGF (bFGF; TRANS GENIC INC., Ltd. Kumamoto, Japan), 100 U/ml penicillin, and 100 μg/ml streptomycin.

Reporter assay

To confirm Wnt/β-catenin signal transcriptional activity in primary BM-MSCs, cells were place at density of 1.8×10^4 cells/cm^2 and treated hexachlorophene at various concentrations one day after. TCF/LEF reporter lentivirus and control renilla lentivirus (SABiosciences, Qiagen N.V., Frederick, MD, USA) were transduced at day 1 and day 5. Reporter assay was performed at day 4 and day8 with Luciferase assay system (Promega) using Luminometer (Berthold Japan K.K., Tokyo, Japan).

Functional tests

Primary BM-MSCs were seeded at a density of 1.8×10^3 cells/cm^2 and treated with each compound at 24 h after seeding. Hepatic differentiation was performed for 8 days. Medium was changed on day4. Urea assay and Periodic acid-Schiff (PAS) staining were

performed as previously described.[2]

Supplementary references

1. Mabuchi Y *et al*. LNGFR+THY-1+VCAM-1hi+ cells reveal functionally distinct subpopulations in mesenchymal stem cells. *Stem Cell Reports* **1**,152-165 (2013)

2. Ishii, K. *et al*. Hepatic differentiation of human bone marrow-derived mesenchymal stem cells by tetracycline-regulated hepatocyte nuclear factor 3beta. *Hepatology* **48**, 597-606 (2008)

Supplementary figures and tables

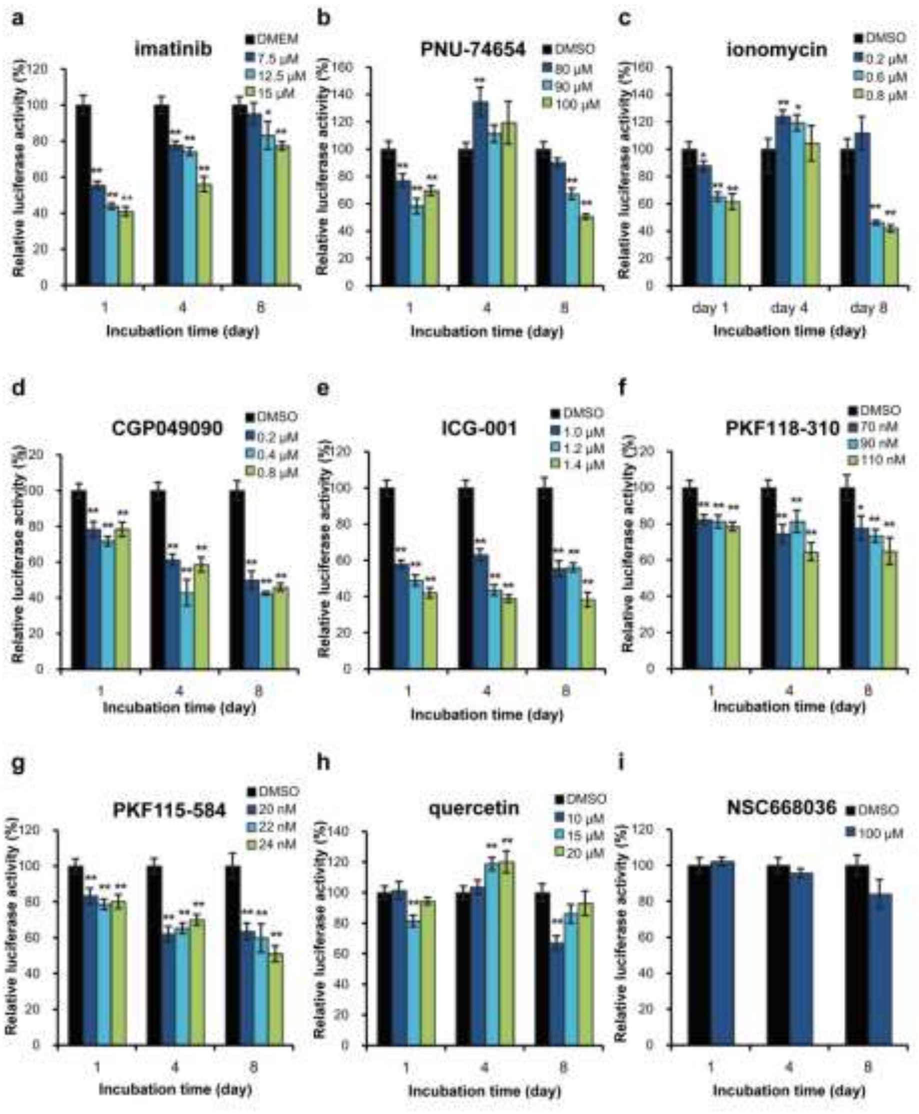

Supplementary Fig.1. The effects of Wnt/β-catenin signal inhibitors on TCF4/β-catenin transcriptional activity.

TCF4/β-catenin transcriptional activity was examined by reporter assay after addition of Wnt//β-catenin signal inhibitors. The effects of (a) imatinib, (b) PNU-74654, (c) ionomycin, (d) CGP049090, (e) ICG-001, (f) PKF118-310, (g) PKF115-584, (h) quercetin, and (i) NSC668036 are analyzed. Data are expressed as the mean ± SE of 8 separate wells. **p* <0.05, ***p* <0.01, compared with 0.1% DMSO (examined by student t-test).

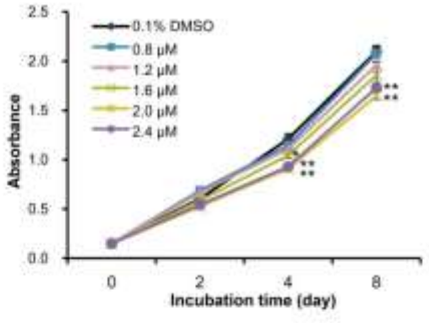

Supplementary Fig.2. Cell viability of hexachlorophene on MSCs.

Cell viability was determined by WST assay following treatment with hexachlorophene for 8 days. Data are expressed as the mean ± SE of 3 separate wells. **p* <0.05, ***p* <0.01, compared with vehicle, 0.1% DMSO (student's t-test).

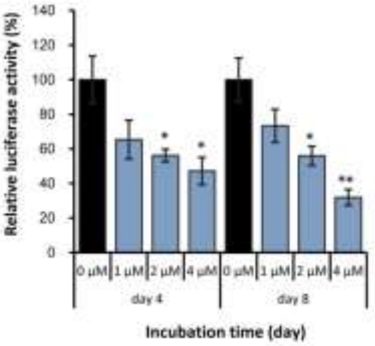

Supplementary Fig.3. Hexachlorophene inhibited Wnt/β-catenin signaling of primary human bone marrow nuclear cells

TCF4/β-catenin transcriptional activities in primary human bone mononuclear cells obtained from the femur head of osteoarthritis of a 60 yr old female patient. **p* <0.05, ***p* <0.01, compared to 0.1% DMSO, at each time point (examined by student's t-test (n=3).

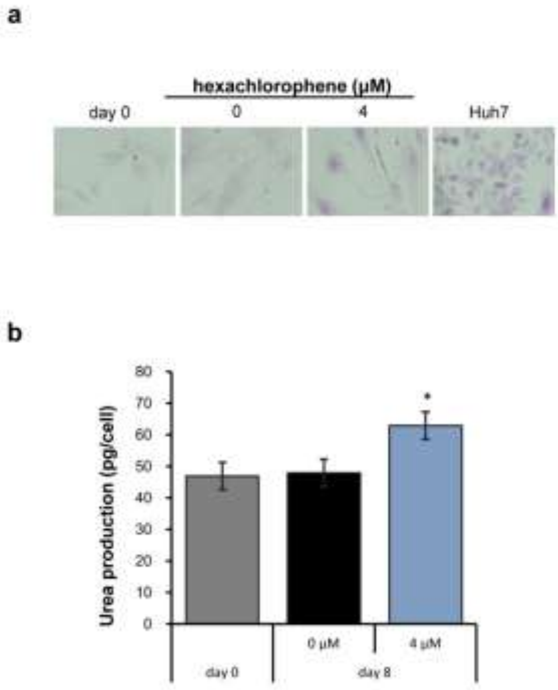

Supplementary Fig.4. Hexachlorophene induced hepatic differentiation of primary human bone marrow nuclear cells

(a) PAS staining of the CD90+CD271+ primary human bone mononuclear cells cultured with hexachlorophene was performed on day 0 and day 8. (b) Urea production of the CD90+CD271+ primary human bone mononuclear cells cultured with hexachlorophene was determined by urea assay. **p* <0.05, compared to 0.1% DMSO on day 8 (n=5).

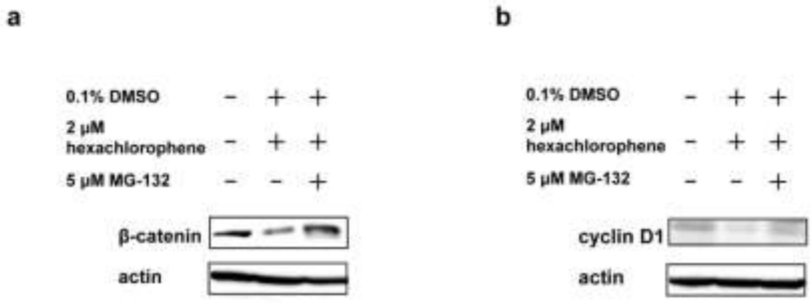

Supplementary Fig.5. β-catenin degradation of hexachlorophene on MSCs.

(a) The protein expression level of β-catenin in UE7T-13 cells. Cells were treated with 2μM hexachlorophene for 48 h. After 24 h incubation, 5 μM MG-132 was added and cultured for 24 h. (b) The protein expression level of cyclin D1.

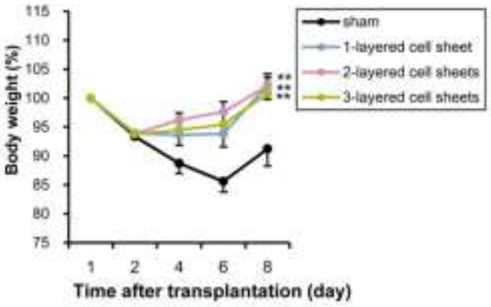

Supplementary Fig.6. Transplantation of hepatic cell sheets accelerated recovery of body weight reduction caused by CCl_4-induced acute liver injury.

Body weight on day1, 2, 4, 6, and 8 in sham-operated and cell sheets transplanted groups. Data are expressed as mean ± SE (n=8-10 on each day) of the percentage when that of day 1 is assumed to be 100%. *p <0.05, **p <0.01, compared to sham-operated mice and examined by ANOVA for repeated measurements.

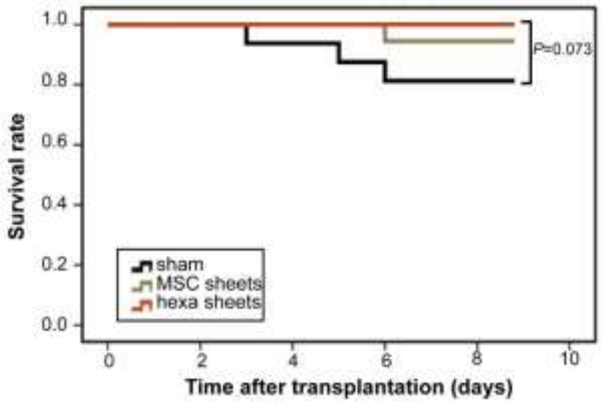

Supplementary Fig.7. Comparison of hexachlorophene-induced hepatic cell sheets transplantation and non-induced MSC sheets transplantation in acute liver failure model in mice.

Survival rate after transplantation (n=16-18). p >0.05, compared among groups (examined by Log-rank test).

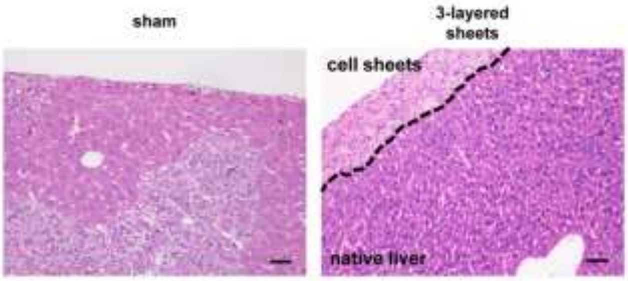

Supplementary Fig.8. Hexachlorophene-induced hepatic cell sheets were still engrafted 8 days after transplantation.

Hematoxylin and eosin staining of sham-operated liver and liver of 3-layered cell sheets-transplanted mice on day 8. Dotted line represents the border between grafted cell sheets and recipient liver tissues. Scale bar =50μm.

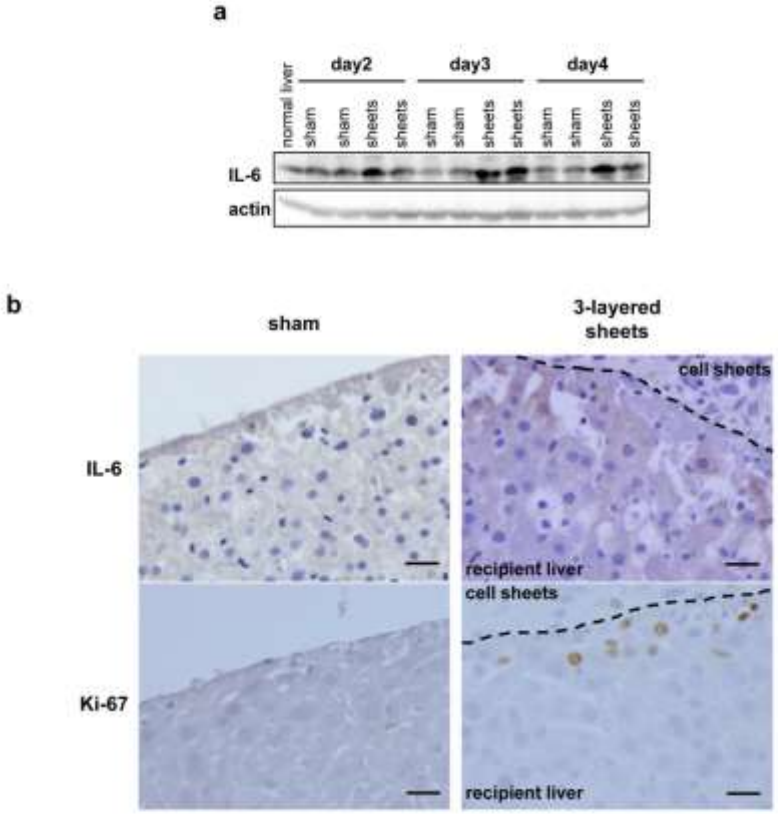

Supplementary Fig.9. Enhanced IL-6 production beneath the grafted cell sheets.

(a) IL-6 protein expression was determined by western blot. Liver lysates including grafted tissues of two mice from each group were used on each day and reacted with mouse specific IL-6 antibody. (b) Immunohistochemistry of IL-6 and Ki-67 beneath the grafted cell sheets. Upper panel showed brown-stained IL-6-positive hepatocytes and lower panel showed Ki-67-positive hepatocytes. Both positive cells were observed beneath the grafted cell sheets on day 2. Dotted line represents the border between grafted cell sheets and recipient tissues. Scale bar=20μm.

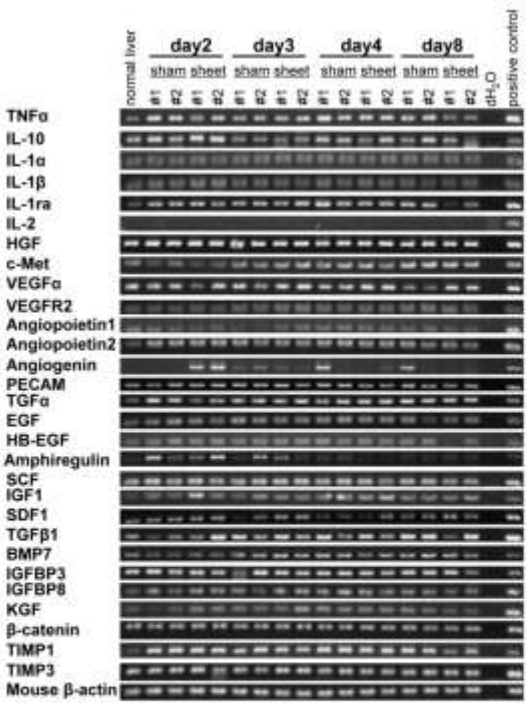

Supplementary Fig. 10. Murine gene expression analysis of cytokines in recipient liver.

RT-PCR was performed using recipient mice native liver not including grafted cell sheets and sham-operated mice liver. Expression of the analyzed genes except angiogenin was not altered between sham-operated mice and cell sheets transplanted recipient liver. Only angiogenin showed higher expression in cell sheets transplanted recipient liver on day 2.

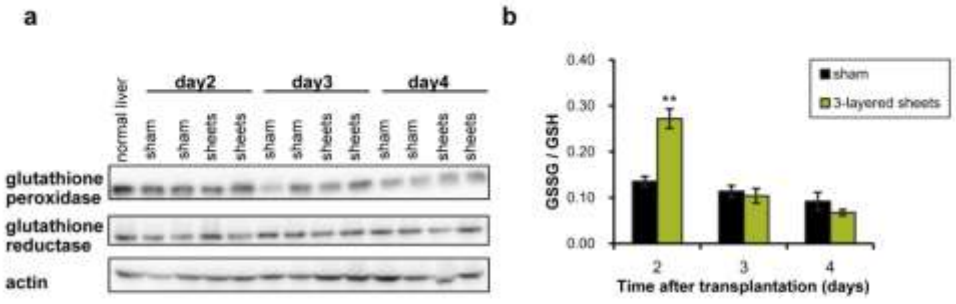

Supplementary Fig. 11. Analysis of GSSG/GSH redox system in recipient liver.

(a) Antioxidant proteins involved in GSSG/GSH system were analyzed by western blot in recipient native liver tissues excluding grafted cell sheets. Both glutathione peroxidase and glutathione reductase were not altered between experimental groups. (b) Oxidative state of recipient liver tissue was analyzed by GSSG/GSH quantification kit. Excess GSSG was recognized in cell sheets transplanted recipient liver. Data are expressed as mean ± SE (n=3). ***p* <0.01, compared with sham-operated mice examined by Mann-Whitney U-test.

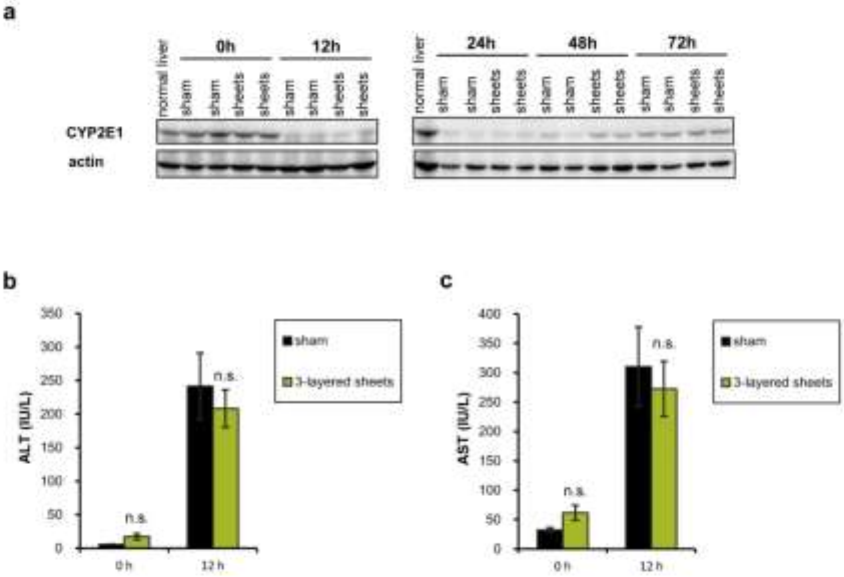

Supplementary Fig.12. CYP2E1 expression in the recipient livers after hepatic cell sheets transplantation and CCl_4 adminstration.

(a) One day before administration of CCl_4, hepatic cell sheets were transplanted onto mouse livers, and then CCl_4 was administered to mice on 0 h. At 12, 24, 48, and 72 h after administration of CCl_4, the sham-operated and cell sheets-transplanted mice were sacrificed, and expression of CYP2E1 was examined by western blotting. (b) Serum ALT of 0 and 12 h after CCl_4 administration. (c) Serum AST of 0 and 12 h after CCl_4 administration. Data are expressed as mean ± SE (n=3 at 0 h and n=5 at 12 h) There were no significant differences between sham and 3-layered sheets-transplanted groups (examined by Mann-Whitney U-test).

Supplementary Table 1. Proteins identified by LC-MSMS

Uniprot ID	Protein description	Log2[Peak area ratio (sheet／sham)]					
		day 2		day 3		day 4	
1433B_MOUSE	14-3-3 protein beta/alpha			0.35	-0.19	0.19	-0.20
1433E_MOUSE	14-3-3 protein epsilon	0.83	2.41	0.02	0.43	1.32	0.34
1433G_MOUSE	14-3-3 protein gamma		4.11				
1433T_MOUSE	14-3-3 protein theta					2.26	
1433Z_MOUSE	14-3-3 protein zeta/delta	1.16	0.03	0.30	0.30	0.44	-1.89
3BHS5_MOUSE	3 beta-hydroxysteroid dehydrogenase type 5	-3.64					
3HAO_MOUSE	3-hydroxyanthranilate 3,4-dioxygenase	0.25	0.62	0.96	0.11	0.18	-0.56
3HIDH_MOUSE	3-hydroxyisobutyrate dehydrogenase, mitochondrial	-2.38	-0.91	-0.58	-0.62	-0.28	-0.22
6PGL_MOUSE	6-phosphogluconolactonase	-0.66			0.71	0.24	0.83
A1AG1_MOUSE	Alpha-1-acid glycoprotein 1	5.03	2.79				
A1AG8_MUSCR	Alpha-1-acid glycoprotein 8			1.50	1.72	3.48	-2.04
A1AT2_MOUSE	Alpha-1-antitrypsin 1-2	0.91	1.33	0.17	-0.03	0.09	-1.74
A1AT3_MOUSE	Alpha-1-antitrypsin 1-3	0.88	1.28	0.21	-0.38	0.56	-1.95
A1AT4_MOUSE	Alpha-1-antitrypsin 1-4		-0.16	-0.07			-0.92
A2M_MOUSE	Alpha-2-macroglobulin	-0.35	-0.98	0.61	0.53	-0.24	-0.68
AADAT_MOUSE	Kynurenine/alpha-aminoadipate aminotransferase, mitochondrial	-1.59	-0.24	0.97			
AASS_MOUSE	Alpha-aminoadipic semialdehyde synthase, mitochondrial	-2.65	-2.47	0.92	-0.11	0.92	-1.60
AATC_MOUSE	Aspartate aminotransferase, cytoplasmic	-0.56	-0.85	0.41	0.19	-0.16	-0.52
AATM_MOUSE	Aspartate aminotransferase, mitochondrial	-0.85	-0.16	0.10	0.38	0.07	-0.69
ABCA4_MOUSE	Retinal-specific ATP-binding cassette transporter		-3.76				
ABCD3_MOUSE	ATP-binding cassette sub-family D member 3	-3.70	-4.47	0.69	0.31	-1.21	0.74
ABHEB_MOUSE	Abhydrolase domain-containing protein 14B	0.30	1.39	0.44	-0.08	1.18	-0.19
ACADL_MOUSE	Long-chain specific acyl-CoA dehydrogenase, mitochondrial	-0.97	-1.16	0.51	0.50	-0.46	-0.37
ACADM_MOUSE	Medium-chain specific acyl-CoA dehydrogenase, mitochondrial	-2.56	-3.04	0.11	0.19	0.03	-0.24
ACADS_MOUSE	Short-chain specific acyl-CoA dehydrogenase, mitochondrial	-2.52	-0.49	0.30	0.55	0.57	-0.99
ACADV_MOUSE	Very long-chain specific acyl-CoA dehydrogenase, mitochondrial	-3.54	-2.77	0.14	0.35	-1.11	-1.38
ACBP_MOUSE	Acyl-CoA-binding protein	2.59	3.56	1.03	1.23	1.87	0.14

ACDSB_MOUSE	Short/branched chain specific acyl-CoA dehydrogenase, mitochondrial	-2.43					
ACLY_MOUSE	ATP-citrate synthase				0.68		
ACOC_MOUSE	Cytoplasmic aconitate hydratase	-1.55	-2.01	0.36	0.19	0.36	0.19
ACON_MOUSE	Aconitate hydratase, mitochondrial	-1.27	-1.05	-0.01	-0.42	0.09	-0.70
ACOT1_MOUSE	Acyl-coenzyme A thioesterase 1	-1.60		-0.47			
ACOT2_MOUSE	Acyl-coenzyme A thioesterase 2, mitochondrial		-0.16		0.37	-1.06	0.02
ACOX1_MOUSE	Peroxisomal acyl-coenzyme A oxidase 1	-1.56	-1.16	0.36	0.31	0.34	-1.52
ACOX2_MOUSE	Peroxisomal acyl-coenzyme A oxidase 2	-2.84	-2.41	0.43	0.23	0.02	-0.28
ACPM_MOUSE	Acyl carrier protein, mitochondrial	5.23	6.45	0.11			
ACSF2_MOUSE	Acyl-CoA synthetase family member 2, mitochondrial	-2.74	-2.55	0.51	-0.32	-0.26	0.24
ACSL1_MOUSE	Long-chain-fatty-acid--CoA ligase 1	-2.30	-3.41	0.61	0.04	-0.99	-0.41
ACSL5_MOUSE	Long-chain-fatty-acid--CoA ligase 5				0.10	-1.10	
ACSM1_MOUSE	Acyl-coenzyme A synthetase ACSM1, mitochondrial	-2.18	-1.20	0.38	0.14	-0.04	-0.77
ACSM5_MOUSE	Acyl-coenzyme A synthetase ACSM5, mitochondrial	-2.47	-1.52				
ACTB_MOUSE	Actin, cytoplasmic 1	-0.90	-0.66			0.04	0.04
ACTC_MOUSE	Actin, alpha cardiac muscle 1		1.43				
ACTG_MOUSE	Actin, cytoplasmic 2			0.17	0.48		
ACTN4_MOUSE	Alpha-actinin-4	0.91	0.87	-0.24	0.50	-0.48	-0.68
ADH1_MOUSE	Alcohol dehydrogenase 1	-0.59	-0.68	0.81	0.98	0.37	-0.07
ADHX_MOUSE	Alcohol dehydrogenase class-3	-0.90	-0.29	0.61	0.19	-0.30	-0.42
ADK_MOUSE	Adenosine kinase	0.90	0.50	0.55	0.39	0.92	-0.61
ADO_MOUSE	Aldehyde oxidase		-1.65				
ADT2_MOUSE	ADP/ATP translocase 2	-3.16	-3.28	0.62	0.23	-0.79	-0.28
ADX_MOUSE	Adrenodoxin, mitochondrial	0.73	1.59	0.30	0.61	0.98	-1.13
AGT2_MOUSE	Alanine--glyoxylate aminotransferase 2, mitochondrial	-2.82	-1.34	0.17	0.10	-0.18	-1.22
AIFM1_MOUSE	Apoptosis-inducing factor 1, mitochondrial	-2.98	-2.50	0.70	-0.50	-0.74	0.36
AK1A1_MOUSE	Alcohol dehydrogenase [NADP+]	-0.33	-0.07	0.53	0.34	0.44	-0.41
AK1CD_MOUSE	Aldo-keto reductase family 1 member C13	0.52	0.40	0.50	0.10	-0.67	-0.92
AK1D1_MOUSE	3-oxo-5-beta-steroid 4-dehydrogenase	1.38	0.53	1.11			
AKAP3_MOUSE	A-kinase anchor protein 3						-0.27
AL1A1_MOUSE	Retinal dehydrogenase 1	-0.84	-1.01	0.51	0.52	0.19	-0.38
AL1A7_MOUSE	Aldehyde dehydrogenase, cytosolic 1	-1.65	-2.04	0.19	-0.41	-0.72	
AL1B1_MOUSE	Aldehyde dehydrogenase X, mitochondrial					-0.62	-1.50
AL1L1_MOUSE	Cytosolic 10-formyltetrahydrofolate dehydrogenase	-1.31	-1.79	0.63	0.21	-0.09	-0.40
AL4A1_MOUSE	Delta-1-pyrroline-5-carboxylate dehydrogenase,	-3.06	-1.95	0.43	0.19	-0.21	-0.24

	mitochondrial						
AL7A1_MOUSE	Alpha-aminoadipic semialdehyde dehydrogenase	-1.14	-0.56	0.21	-0.28	-0.42	-0.79
AL8A1_MOUSE	Aldehyde dehydrogenase family 8 member A1	-0.36	-1.92	0.89	0.26	-0.23	-1.53
AL9A1_MOUSE	4-trimethylaminobutyraldehyde dehydrogenase	-1.54	-0.61	0.43	0.20	0.16	-0.55
ALAT1_MOUSE	Alanine aminotransferase 1	-3.74	-2.43	0.08	-0.19	-1.61	0.31
ALAT2_MOUSE	Alanine aminotransferase 2	-2.40	-2.30		-0.35		-1.88
ALBU_MOUSE	Serum albumin	0.21	0.54	-0.35	-0.24	-0.32	-1.48
ALDH2_MOUSE	Aldehyde dehydrogenase, mitochondrial	-2.01	-1.19	0.34	0.26	-0.09	-1.14
ALDOA_MOUSE	Fructose-bisphosphate aldolase A	-0.95	-1.59	-0.39	-0.14	0.63	-0.42
ALDOB_MOUSE	Fructose-bisphosphate aldolase B	-0.81	-0.33	0.51	0.07	0.01	-0.61
AMACR_MOUSE	Alpha-methylacyl-CoA racemase	-2.31				-1.52	3.15
AMBP_MOUSE	Protein AMBP	2.91					-1.51
AMPL_MOUSE	Cytosol aminopeptidase	-0.69	-0.87	0.17	-0.27	0.18	-0.48
AN32A_MOUSE	Acidic leucine-rich nuclear phosphoprotein 32 family member A	0.81	1.49				
ANT3_MOUSE	Antithrombin-III	-0.08		0.39		0.07	-0.44
ANXA5_MOUSE	Annexin A5	0.79	1.06		0.18	-0.64	
ANXA6_MOUSE	Annexin A6	5.34	5.23		-0.09	-0.42	0.27
AOFB_MOUSE	Amine oxidase [flavin-containing] B				-0.17	-0.71	
AP4E1_MOUSE	AP-4 complex subunit epsilon-1		-2.22				
APOA1_MOUSE	Apolipoprotein A-I	1.10	-0.01	0.80	0.31	0.88	-0.80
APOA2_MOUSE	Apolipoprotein A-II	-0.41	-0.66				
APOA4_MOUSE	Apolipoprotein A-IV	1.39	1.08	0.10	-0.35	-0.11	-0.98
APOA5_MOUSE	Apolipoprotein A-V				1.32		
APOC1_MOUSE	Apolipoprotein C-I	2.29	0.04	0.67		0.38	0.39
APOC3_MOUSE	Apolipoprotein C-III	-1.35	-2.56	-2.44		-1.87	-3.56
APOE_MOUSE	Apolipoprotein E	0.42	-0.86		0.30	-0.30	-0.56
APOH_MOUSE	Beta-2-glycoprotein 1	-0.56	3.02				
APT_MOUSE	Adenine phosphoribosyltransferase					-0.37	
ARF1_MOUSE	ADP-ribosylation factor 1	4.40		0.44			
ARF3_MOUSE	ADP-ribosylation factor 3					-0.05	-1.83
ARF5_MOUSE	ADP-ribosylation factor 5				1.50		
ARGI1_MOUSE	Arginase-1	0.61	1.15	0.33	0.40	0.58	-0.70
ARK72_MOUSE	Aflatoxin B1 aldehyde reductase member 2	-1.08	-1.12	0.45	-0.59		
ARLY_MOUSE	Argininosuccinate lyase	-0.08	-0.09	0.55	-0.08	-0.01	-1.26
ARP3_MOUSE	Actin-related protein 3			0.03			0.11

ARP3B_MOUSE	Actin-related protein 3B		-0.97		1.31		
ARPC4_MOUSE	Actin-related protein 2/3 complex subunit 4		-0.71				
ASGR1_MOUSE	Asialoglycoprotein receptor 1		-0.99			-0.46	
ASSY_MOUSE	Argininosuccinate synthase	-1.42	-1.34	0.49	0.38	0.35	-0.73
AT131_MOUSE	Probable cation-transporting ATPase 13A1					-0.25	
AT1A1_MOUSE	Sodium/potassium-transporting ATPase subunit alpha-1				1.24		
AT5F1_MOUSE	ATP synthase subunit b, mitochondrial	-2.24	-1.89		0.32	0.00	1.05
ATOX1_MOUSE	Copper transport protein ATOX1	1.27	1.26	0.86	0.32	-0.18	-0.32
ATP5E_MOUSE	ATP synthase subunit epsilon, mitochondrial	-1.35	-0.30	-0.42	-0.56	-0.39	-1.21
ATP5H_MOUSE	ATP synthase subunit d, mitochondrial	-0.84	-0.18	0.25	0.02	-0.24	0.58
ATP5I_MOUSE	ATP synthase subunit e, mitochondrial	-0.84	-0.05	-1.09	-0.10	-0.68	-0.20
ATP5J_MOUSE	ATP synthase-coupling factor 6, mitochondrial	3.49	2.38	0.59	0.18		-0.85
ATP5L_MOUSE	ATP synthase subunit g, mitochondrial	-2.83	-2.39		-0.17	-0.47	0.68
ATP8_MOUSE	ATP synthase protein 8	-5.21	-3.94		-0.48		
ATPA_MOUSE	ATP synthase subunit alpha, mitochondrial	-1.05	-0.50	0.23	0.19	-0.12	0.08
ATPB_MOUSE	ATP synthase subunit beta, mitochondrial	0.79	1.83	0.03	0.22	0.16	-1.01
ATPD_MOUSE	ATP synthase subunit delta, mitochondrial	2.39	3.46	0.35	1.14	1.16	-1.37
ATPG_MOUSE	ATP synthase subunit gamma, mitochondrial	-2.72	-2.48	0.29	-0.30	-1.07	0.05
ATPK_MOUSE	ATP synthase subunit f, mitochondrial	-3.92	-2.72		-0.03		0.13
ATPO_MOUSE	ATP synthase subunit O, mitochondrial	-3.22	-3.08	0.02	-0.25	-0.56	0.66
ATR_MOUSE	Serine/threonine-protein kinase ATR	0.52					
ATTY_MOUSE	Tyrosine aminotransferase			0.33			
AUHM_MOUSE	Methylglutaconyl-CoA hydratase, mitochondrial	-1.35					
B2MG_MOUSE	Beta-2-microglobulin	0.67		0.04			
B3AT_MOUSE	Band 3 anion transport protein				-0.94		0.92
BDH_MOUSE	D-beta-hydroxybutyrate dehydrogenase, mitochondrial	-3.16	-2.98	1.11	0.58	-0.10	0.13
BHMT1_MOUSE	Betaine--homocysteine S-methyltransferase 1	-1.13	-0.66	0.20	0.04	0.48	-0.69
BLVRB_MOUSE	Flavin reductase (NADPH)	0.46	0.98	-0.08	-0.37	0.79	0.10
BPHL_MOUSE	Valacyclovir hydrolase	-1.22	-0.18	0.01	-0.45	0.00	-0.72
BRAF_MOUSE	Serine/threonine-protein kinase B-raf	1.52					
BUP1_MOUSE	Beta-ureidopropionase	-1.54	-0.97	0.36		0.16	0.38
C1QBP_MOUSE	Complement component 1 Q subcomponent-binding protein, mitochondrial	0.00	0.46	0.04	0.60	-1.29	-0.72
C1TC_MOUSE	C-1-tetrahydrofolate synthase, cytoplasmic	-1.37	-1.71	-0.08	0.33	-0.56	0.42
CAH1_MOUSE	Carbonic anhydrase 1	-9.97				2.54	0.90
CAH2_MOUSE	Carbonic anhydrase 2	1.57	1.77	-0.40	-0.50	1.59	0.37

CAH3_MOUSE	Carbonic anhydrase 3	1.39	1.98	0.97	0.54	1.54	-0.93
CAH5A_MOUSE	Carbonic anhydrase 5A, mitochondrial		1.25	0.47			
CALM_MOUSE	Calmodulin	3.66	4.26	0.08	0.34	2.94	0.12
CALR_MOUSE	Calreticulin	1.41	1.26	0.80	0.68	0.92	-0.13
CALU_MOUSE	Calumenin	1.97	1.64	0.79	0.32	0.13	0.21
CALX_MOUSE	Calnexin	0.79	0.51	0.99	1.58	-0.97	0.81
CAN10_MOUSE	Calpain-10	-5.38					
CAP1_MOUSE	Adenylyl cyclase-associated protein 1				-0.23	-0.41	0.86
CASR_MOUSE	Extracellular calcium-sensing receptor			-1.45	-1.99	-1.56	-2.97
CATA_MOUSE	Catalase	-1.28	-0.71	-0.24	-0.35	0.33	-0.82
CATB_MOUSE	Cathepsin B	1.24	1.92	-0.69		1.75	
CATD_MOUSE	Cathepsin D	-0.20	-0.21	-0.41	-0.08	0.13	0.20
CATZ_MOUSE	Cathepsin Z			-0.30			
CAZA2_MOUSE	F-actin-capping protein subunit alpha-2			0.29	0.09		
CBR1_MOUSE	Carbonyl reductase [NADPH] 1	-0.82	-0.36	-0.06	0.14	0.05	0.14
CBR3_MOUSE	Carbonyl reductase [NADPH] 3			-0.73		1.23	
CBR4_MOUSE	Carbonyl reductase family member 4	-5.51	-9.97				
CBS_MOUSE	Cystathionine beta-synthase					-0.03	
CCD58_MOUSE	Coiled-coil domain-containing protein 58	1.74	2.75				
CD027_MOUSE	UPF0609 protein C4orf27 homolog						-0.21
CD47_MOUSE	Leukocyte surface antigen CD47	2.21	1.55				
CDK11_MOUSE	Cyclin-dependent kinase 11			-0.26			
CERU_MOUSE	Ceruloplasmin	-0.85		0.04	0.44	-0.69	-1.39
CES3_MOUSE	Carboxylesterase 3	-0.30	-0.89	0.85	0.76	0.29	-0.60
CF115_MOUSE	Costars family protein C6orf115 homolog					1.22	
CFAH_MOUSE	Complement factor H			-0.08			
CGL_MOUSE	Cystathionine gamma-lyase	-0.79	-0.67	0.71	0.33	0.06	-0.76
CH10_MOUSE	10 kDa heat shock protein, mitochondrial	1.77	1.85	-0.04	-0.36	0.65	-0.51
CH60_MOUSE	60 kDa heat shock protein, mitochondrial	0.14	0.32	0.10	-0.22	-0.07	-0.41
CHDH_MOUSE	Choline dehydrogenase, mitochondrial	-2.85	-2.02				
CHSP1_MOUSE	Calcium-regulated heat stable protein 1						0.47
CISD1_MOUSE	CDGSH iron-sulfur domain-containing protein 1					0.61	
CISY_MOUSE	Citrate synthase, mitochondrial	-2.97	-1.61	0.20	-0.42	-0.53	-1.05
CK054_MOUSE	Ester hydrolase C11orf54 homolog	0.92	0.76			-1.99	-0.92
CLAP2_MOUSE	CLIP-associating protein 2						-1.60
CLCA_MOUSE	Clathrin light chain A	0.80	1.10				

CLH_MOUSE	Clathrin heavy chain 1	-1.11	-2.32	1.48	0.27	-0.18	-0.22
CLUS_MOUSE	Clusterin		0.27	0.26	0.44	0.86	-0.57
CLYBL_MOUSE	Citrate lyase subunit beta-like protein, mitochondrial	0.69			0.26		
CMBL_MOUSE	Carboxymethylenebutenolidase homolog		0.81	0.59	0.42		-1.47
CMC2_MOUSE	Calcium-binding mitochondrial carrier protein Aralar2	-2.27	-3.15		-0.18	-0.93	
CNBP_MOUSE	Cellular nucleic acid-binding protein		1.18	0.62	0.31	0.34	-0.06
CNPY2_MOUSE	Protein canopy homolog 2		0.85				
CO3_MOUSE	Complement C3	-1.16	-0.73	1.40	0.78	0.79	-1.03
COF1_MOUSE	Cofilin-1	2.12	0.70	0.21	0.55	1.88	0.07
COG4_MOUSE	Conserved oligomeric Golgi complex subunit 4			0.65			
COMT_MOUSE	Catechol O-methyltransferase		2.19	-0.45	0.76	-1.19	-1.08
COPG2_MOUSE	Coatomer subunit gamma-2		-3.27	0.67	0.57	-0.66	
COTL1_MOUSE	Coactosin-like protein			-0.90			
COX2_MOUSE	Cytochrome c oxidase subunit 2	-1.92	-3.00	1.50	0.34	0.59	0.12
COX41_MOUSE	Cytochrome c oxidase subunit 4 isoform 1, mitochondrial	0.40	0.77		0.67	1.11	
COX5A_MOUSE	Cytochrome c oxidase subunit 5A, mitochondrial	0.94	1.31		-0.06	-0.06	
COX5B_MOUSE	Cytochrome c oxidase subunit 5B, mitochondrial	2.64	4.46	0.69	0.65	1.11	
COX6C_MOUSE	Cytochrome c oxidase subunit 6C	-2.48	-3.35		-0.45	-0.61	0.44
CP013_MOUSE	UPF0585 protein C16orf13 homolog	-0.51		-0.41			
CP1A2_MOUSE	Cytochrome P450 1A2					0.44	
CP240_MOUSE	Cytochrome P450 2C40					-0.91	
CP254_MOUSE	Cytochrome P450 2C54			2.12		1.27	
CP27A_MOUSE	Sterol 26-hydroxylase, mitochondrial	-9.97					
CP2CT_MOUSE	Cytochrome P450 2C29	-2.55			0.50	-0.09	-0.87
CP2D9_MOUSE	Cytochrome P450 2D9	-1.10	-2.47	1.65	-0.14	-1.13	0.56
CP2DA_MOUSE	Cytochrome P450 2D10	-2.93	-2.82	0.46	0.08	-1.23	0.42
CP2DQ_MOUSE	Cytochrome P450 2D26	-0.77	-2.79	1.08	0.34	-0.54	0.09
CP2E1_MOUSE	Cytochrome P450 2E1	-2.16			1.85	-0.30	0.24
CP2F2_MOUSE	Cytochrome P450 2F2	-3.78	-5.51		-1.65	-2.52	
CP341_MOUSE	Cytochrome P450 3A41	-4.68					
CP3AB_MOUSE	Cytochrome P450 3A11		-9.97		1.64	-1.78	
CP4AE_MOUSE	Cytochrome P450 4A14				-0.27		
CP4CA_MOUSE	Cytochrome P450 4A12A		-4.80		-0.49	-0.77	
CPNE9_MOUSE	Copine-9					-0.44	
CPSM_MOUSE	Carbamoyl-phosphate synthase [ammonia],	-1.26	-1.05	0.30	0.13	0.28	-0.23

	mitochondrial						
CPT1A_MOUSE	Carnitine O-palmitoyltransferase 1, liver isoform				-0.08	-0.79	
CPT2_MOUSE	Carnitine O-palmitoyltransferase 2, mitochondrial	-2.20	-0.28		0.23		
CREG1_MOUSE	Protein CREG1	1.64	1.76	0.10	0.83	0.97	-1.07
CS010_MOUSE	UPF0556 protein C19orf10 homolog	2.36	1.31			0.45	
CX6B1_MOUSE	Cytochrome c oxidase subunit 6B1	-0.35	-0.13	0.19	0.03	-0.31	0.19
CX7A2_MOUSE	Cytochrome c oxidase subunit 7A2, mitochondrial	-0.36	1.95		0.22		
CY1_MOUSE	Cytochrome c1, heme protein, mitochondrial	-2.78	-2.88		-0.25	-1.17	-0.27
CYB5_MOUSE	Cytochrome b5	-0.16	0.06	0.41	0.29	-0.66	0.82
CYB5B_MOUSE	Cytochrome b5 type B					0.84	
CYC_MOUSE	Cytochrome c, somatic	0.95	2.34	0.65	-0.86	0.71	-0.07
CYTB_MOUSE	Cystatin-B			0.06			-0.10
DAG1_MOUSE	Dystroglycan			0.18		-9.97	-0.74
DBPA_MOUSE	DNA-binding protein A		4.40				
DCXR_MOUSE	L-xylulose reductase		-0.34	0.69	0.71	1.66	
DDX1_MOUSE	ATP-dependent RNA helicase DDX1				1.42		
DDX25_MOUSE	ATP-dependent RNA helicase DDX25		-0.02		0.65		
DDX3L_MOUSE	Putative ATP-dependent RNA helicase Pl10						-0.17
DDX3X_MOUSE	ATP-dependent RNA helicase DDX3X					-0.01	
DDX3Y_MOUSE	ATP-dependent RNA helicase DDX3Y			0.47	0.78		
DDX5_MOUSE	Probable ATP-dependent RNA helicase DDX5					-0.01	
DECR_MOUSE	2,4-dienoyl-CoA reductase, mitochondrial	-2.12	-1.47	0.66	-0.34	-0.29	-0.45
DEST_MOUSE	Destrin			0.15	1.25	0.28	
DHAK_MOUSE	Bifunctional ATP-dependent dihydroxyacetone kinase/FAD-AMP lyase (cyclizing)	-1.23	-1.97	0.27	-0.08	-0.32	-0.16
DHB13_MOUSE	17-beta-hydroxysteroid dehydrogenase 13	-4.11	-3.92	1.02	0.03	-1.16	1.01
DHB4_MOUSE	Peroxisomal multifunctional enzyme type 2	-1.18	-1.21	0.11	0.12	-0.57	-0.01
DHB5_MOUSE	Estradiol 17 beta-dehydrogenase 5	0.56	0.50	0.67	0.50	0.37	0.22
DHB8_MOUSE	Estradiol 17-beta-dehydrogenase 8	-1.05	-1.41				
DHDH_MOUSE	Trans-1,2-dihydrobenzene-1,2-diol dehydrogenase					0.28	
DHE3_MOUSE	Glutamate dehydrogenase 1, mitochondrial	-1.84	-1.60	0.05	-0.26	-0.19	-0.50
DHI1_MOUSE	Corticosteroid 11-beta-dehydrogenase isozyme 1	-2.90	-2.10		0.17		
DHPR_MOUSE	Dihydropteridine reductase	-0.58	1.62	0.19	0.63	0.28	-0.32
DHRS4_MOUSE	Dehydrogenase/reductase SDR family member 4	-1.44	-1.14	0.50	-0.04	-0.34	-0.24
DHSA_MOUSE	Succinate dehydrogenase [ubiquinone] flavoprotein subunit, mitochondrial	-1.47	-0.43	0.11	-0.05	0.00	-0.53

DHSB_MOUSE	Succinate dehydrogenase [ubiquinone] iron-sulfur subunit, mitochondrial	-0.53	-1.61	0.03	0.02	-0.65	-0.64
DHSO_MOUSE	Sorbitol dehydrogenase	-1.19	-0.79	0.62	0.37	0.22	0.08
DHX40_MOUSE	Probable ATP-dependent RNA helicase DHX40					-1.17	
DLDH_MOUSE	Dihydrolipoyl dehydrogenase, mitochondrial	-1.37	-0.50	0.16	-0.13	-0.08	-0.33
DOPD_MOUSE	D-dopachrome decarboxylase	1.27	1.33	0.50	0.09	0.68	-0.55
DPP2_MOUSE	Dipeptidyl peptidase 2			0.35			-0.06
DPYD_MOUSE	Dihydropyrimidine dehydrogenase [NADP+]	-3.35	-1.60				
DPYS_MOUSE	Dihydropyrimidinase	0.10	0.30	0.09	0.22		
DX39A_MOUSE	ATP-dependent RNA helicase DDX39A			0.19			
DX39B_MOUSE	Spliceosome RNA helicase Ddx39b				1.02	0.43	-0.02
DYH17_MOUSE	Dynein heavy chain 17, axonemal		-0.78				
DYL1_MOUSE	Dynein light chain 1, cytoplasmic					-1.54	
DYR1A_MOUSE	Dual specificity tyrosine-phosphorylation-regulated kinase 1A					8.01	
ECH1_MOUSE	Delta(3,5)-Delta(2,4)-dienoyl-CoA isomerase, mitochondrial	-0.31	-0.30	-0.18	0.70	-0.19	-0.95
ECHA_MOUSE	Trifunctional enzyme subunit alpha, mitochondrial	-2.10	-2.11	0.26	0.32	-0.31	-0.20
ECHB_MOUSE	Trifunctional enzyme subunit beta, mitochondrial	-2.40	-1.89	-0.04	0.03	-0.63	-0.06
ECHD2_MOUSE	Enoyl-CoA hydratase domain-containing protein 2, mitochondrial	-1.35	-1.10				
ECHD3_MOUSE	Enoyl-CoA hydratase domain-containing protein 3, mitochondrial		-1.06				
ECHM_MOUSE	Enoyl-CoA hydratase, mitochondrial	-1.28	-0.03	0.42	0.41	0.88	-0.31
ECHP_MOUSE	Peroxisomal bifunctional enzyme	-2.71	-2.19	-0.27	-0.66	-1.43	-0.40
ECI1_MOUSE	Enoyl-CoA delta isomerase 1, mitochondrial	-0.67	-1.46	-0.05	-0.22	-0.93	-0.64
ECI2_MOUSE	Enoyl-CoA delta isomerase 2, mitochondrial	-0.99	0.04	-0.53			
EF1A1_MOUSE	Elongation factor 1-alpha 1	-0.65	-0.21	0.38	0.67	0.33	-0.18
EF1A2_MOUSE	Elongation factor 1-alpha 2	-9.97	-9.97	-8.38	-6.51	-9.97	-7.38
EF1B_MOUSE	Elongation factor 1-beta	0.96	1.35	-0.22	0.27	0.04	-0.17
EF1D_MOUSE	Elongation factor 1-delta	0.98	2.04	0.44	0.85	0.97	0.37
EF1G_MOUSE	Elongation factor 1-gamma	-1.56	-2.17	0.27	0.54	-0.17	0.12
EF2_MOUSE	Elongation factor 2	-1.38	-1.98	0.46	0.43	-0.38	-0.13
EFHD2_MOUSE	EF-hand domain-containing protein D2	3.56	3.23	-0.01	2.39	2.37	
EFTU_MOUSE	Elongation factor Tu, mitochondrial	-1.84	-1.11		0.03	-0.79	1.00
EGFR_MOUSE	Epidermal growth factor receptor	-0.64	1.04	0.15	1.78		

EHD3_MOUSE	EH domain-containing protein 3		3.02				
EIF1_MOUSE	Eukaryotic translation initiation factor 1	5.57	4.44	1.03	1.03	2.46	
EIF3G_MOUSE	Eukaryotic translation initiation factor 3 subunit G		3.08	-0.11		1.38	
ENOA_MOUSE	Alpha-enolase	-0.05	0.21	0.05	0.42	-0.06	-0.35
ENPL_MOUSE	Endoplasmin	1.34	0.68	0.99	0.88	0.23	0.32
ENTP5_MOUSE	Ectonucleoside triphosphate diphosphohydrolase 5					0.60	
ERC6L_MOUSE	DNA excision repair protein ERCC-6-like						-3.02
ERP29_MOUSE	Endoplasmic reticulum resident protein 29	1.42	1.93	0.27	0.91	0.88	0.65
ERP44_MOUSE	Endoplasmic reticulum resident protein 44					1.88	
ES1_MOUSE	ES1 protein homolog, mitochondrial	-0.60	0.01	0.34	0.70	1.03	-0.41
EST1_MOUSE	Liver carboxylesterase 1	-3.40	-0.51				
EST1C_MOUSE	Carboxylesterase 1C	-1.08		-0.46		1.34	
EST1E_MOUSE	Carboxylesterase 1E		0.50				
EST3A_MOUSE	Carboxylesterase 3A	-0.60	-0.42	0.77	0.13	0.84	-0.71
EST3B_MOUSE	Carboxylesterase 3B	-0.40	-1.05	0.45	-0.27		-1.37
ESTD_MOUSE	S-formylglutathione hydrolase	0.18	0.00	-0.09	0.63	0.38	-0.72
ETAA1_MOUSE	Ewing's tumor-associated antigen 1 homolog			-0.67			
ETFA_MOUSE	Electron transfer flavoprotein subunit alpha, mitochondrial	-1.05	-0.39	0.51	0.16	0.28	-0.20
ETFB_MOUSE	Electron transfer flavoprotein subunit beta	-0.68	0.67	0.65	-0.01	0.42	-0.18
ETFD_MOUSE	Electron transfer flavoprotein-ubiquinone oxidoreductase, mitochondrial	-2.22	-1.66	-0.59	0.09	-1.33	0.11
ETHE1_MOUSE	Protein ETHE1, mitochondrial	-0.41	-1.47	0.06			
ETUD1_MOUSE	Elongation factor Tu GTP-binding domain-containing protein 1			-0.20		-0.28	
EWS_MOUSE	RNA-binding protein EWS	3.45	8.59				
EZRI_MOUSE	Ezrin			0.72			-0.17
F10A1_MOUSE	Hsc70-interacting protein	1.16	0.78	0.57	0.49	0.35	0.67
F136A_MOUSE	Protein FAM136A	3.29	3.18	-0.15			
F16P1_MOUSE	Fructose-1,6-bisphosphatase 1	-0.17	0.77	0.45	0.58	0.45	-0.66
FAAA_MOUSE	Fumarylacetoacetase	-0.74	-0.29	0.20	0.07	0.27	-0.55
FABP4_MOUSE	Fatty acid-binding protein, adipocyte		5.60				
FABP5_MOUSE	Fatty acid-binding protein, epidermal	2.97	3.37				
FABPI_MOUSE	Fatty acid-binding protein, intestinal	3.44	2.75				
FABPL_MOUSE	Fatty acid-binding protein, liver	1.57	1.76	0.54	0.36	0.23	-0.36
FAHD1_MOUSE	Acylpyruvase FAHD1, mitochondrial	-0.18	-0.31	0.30			-0.26

FAM25_MOUSE	Protein FAM25					1.02	
FAS_MOUSE	Fatty acid synthase	-4.08	-2.41	1.22	1.20	-0.52	0.85
FETUA_MOUSE	Alpha-2-HS-glycoprotein	0.39	0.89	0.72	0.57	0.59	-0.74
FETUB_MOUSE	Fetuin-B		0.96				
FHAD1_MOUSE	Forkhead-associated domain-containing protein 1	-5.11					
FIBB_MOUSE	Fibrinogen beta chain	2.22	4.01	0.31	0.64		-0.55
FIBG_MOUSE	Fibrinogen gamma chain	2.67	2.74	0.39	1.46		
FKBP4_MOUSE	Peptidyl-prolyl cis-trans isomerase FKBP4					1.02	
FLNA_MOUSE	Filamin-A			-0.22			
FLNB_MOUSE	Filamin-B				0.64	-0.02	-4.97
FMO1_MOUSE	Dimethylaniline monooxygenase [N-oxide-forming] 1					-0.22	
FMO5_MOUSE	Dimethylaniline monooxygenase [N-oxide-forming] 5	-2.82	-4.92		-0.35	-1.21	-0.14
FPPS_MOUSE	Farnesyl pyrophosphate synthase		-1.98	1.06	-0.02	1.45	0.09
FRIH_MOUSE	Ferritin heavy chain	0.65	-0.05	-0.73	0.06	-0.49	-1.25
FRIL1_MOUSE	Ferritin light chain 1	0.67	0.36	-0.11	0.22	-0.27	-0.58
FTCD_MOUSE	Formimidoyltransferase-cyclodeaminase	-1.83	-2.16	-0.20	0.17	-0.34	-0.09
FUMH_MOUSE	Fumarate hydratase, mitochondrial	-0.18	0.38	0.27	-0.47	0.87	-0.44
FUS_MOUSE	RNA-binding protein FUS		4.70			2.79	
G3P_MOUSE	Glyceraldehyde-3-phosphate dehydrogenase	0.03	-0.36	0.36	0.19	0.02	0.38
G6PI_MOUSE	Glucose-6-phosphate isomerase	0.31	-0.71	-0.07	0.91	-0.13	0.04
GABT_MOUSE	4-aminobutyrate aminotransferase, mitochondrial	-1.63	-0.16	0.33	0.41	-0.23	-1.25
GALK1_MOUSE	Galactokinase						-1.50
GALM_MOUSE	Aldose 1-epimerase				0.68	-0.22	-0.94
GANAB_MOUSE	Neutral alpha-glucosidase AB			0.86			-1.21
GBLP_MOUSE	Guanine nucleotide-binding protein subunit beta-2-like 1			2.01	0.79	1.15	2.92
GCDH_MOUSE	Glutaryl-CoA dehydrogenase, mitochondrial	-3.16	-2.08	0.45	-0.13	-0.46	-0.30
GCKR_MOUSE	Glucokinase regulatory protein				0.26		
GCSH_MOUSE	Glycine cleavage system H protein, mitochondrial	0.55	1.28			-9.97	
GDIB_MOUSE	Rab GDP dissociation inhibitor beta	-1.01	-1.22	0.22	0.59	-0.04	-0.28
GDIR1_MOUSE	Rho GDP-dissociation inhibitor 1	-1.61	0.72	-0.43	0.46	0.07	-0.57
GELS_MOUSE	Gelsolin			-0.94			
GFRP_MOUSE	GTP cyclohydrolase 1 feedback regulatory protein	-9.97	-0.01				
GGLO_MOUSE	L-gulonolactone oxidase	-0.28				-0.77	1.08
GLNA_MOUSE	Glutamine synthetase	-3.84	-2.40				
GLO2_MOUSE	Hydroxyacylglutathione hydrolase, mitochondrial	-0.08	0.44	0.38	0.31	0.50	-0.30

GLOD4_MOUSE	Glyoxalase domain-containing protein 4		-0.47		0.03	0.27	
GLRX1_MOUSE	Glutaredoxin-1	1.79	2.09				
GLRX5_MOUSE	Glutaredoxin-related protein 5, mitochondrial	-0.36		0.22			
GLSL_MOUSE	Glutaminase liver isoform, mitochondrial		-1.56				
GLU2B_MOUSE	Glucosidase 2 subunit beta	1.37				0.28	
GLYAL_MOUSE	Glycine N-acyltransferase-like protein	-2.08	0.66	-0.41	-0.44		-0.60
GLYAT_MOUSE	Glycine N-acyltransferase	0.15	1.21	0.13	0.45	0.67	-1.84
GLYC_MOUSE	Serine hydroxymethyltransferase, cytosolic	-0.89	-0.63	0.59	0.36	0.19	0.74
GNAO_MOUSE	Guanine nucleotide-binding protein G(o) subunit alpha					-1.45	
GNMT_MOUSE	Glycine N-methyltransferase	-1.23	-0.59	0.31	0.33	0.42	-1.24
GPDA_MOUSE	Glycerol-3-phosphate dehydrogenase [NAD+], cytoplasmic	-2.47	-1.99	0.41	0.20	-0.17	-0.64
GPDM_MOUSE	Glycerol-3-phosphate dehydrogenase, mitochondrial				-0.31		-1.02
GPX1_MOUSE	Glutathione peroxidase 1	-0.48	-0.37	0.22	-0.53	0.35	-0.38
GRHPR_MOUSE	Glyoxylate reductase/hydroxypyruvate reductase	0.07	0.58	0.79	0.92	0.48	-0.03
GRN_MOUSE	Granulins	-0.28	-0.39	0.16	0.64	-0.05	-0.09
GRP75_MOUSE	Stress-70 protein, mitochondrial	-1.52	-0.91	0.29	0.00	0.05	0.28
GRP78_MOUSE	78 kDa glucose-regulated protein	0.92	0.65	0.98	0.43	0.54	0.19
GRPE1_MOUSE	GrpE protein homolog 1, mitochondrial	1.13	-0.54	0.22	0.09	-0.10	-0.37
GSH0_MOUSE	Glutamate--cysteine ligase regulatory subunit		-0.62				
GSH1_MOUSE	Glutamate--cysteine ligase catalytic subunit	-0.85	-0.32	0.53	0.54	-0.15	-0.83
GSTA2_MOUSE	Glutathione S-transferase A2	0.60					
GSTA3_MOUSE	Glutathione S-transferase A3	0.21	0.49	0.70	0.79	0.40	-0.26
GSTK1_MOUSE	Glutathione S-transferase kappa 1	-2.46	0.47	-0.02	0.42	-0.09	-0.58
GSTM1_MOUSE	Glutathione S-transferase Mu 1	-0.51	0.36	0.38	0.31	0.33	-0.81
GSTM2_MOUSE	Glutathione S-transferase Mu 2					0.82	
GSTM4_MOUSE	Glutathione S-transferase Mu 3					-1.27	
GSTM7_MOUSE	Glutathione S-transferase Mu 7			0.73			
GSTP1_MOUSE	Glutathione S-transferase P 1	-0.47	-0.14	0.44	-0.04	0.46	-0.92
GSTT1_MOUSE	Glutathione S-transferase theta-1	-1.73	-1.35		-1.11	-0.24	0.59
GSTT2_MOUSE	Glutathione S-transferase theta-2	-1.94	-2.09				
GTR2_MOUSE	Solute carrier family 2, facilitated glucose transporter member 2	-2.45	-1.49				
GYS2_MOUSE	Glycogen [starch] synthase, liver	-3.94					
H10_MOUSE	Histone H1.0		-3.28				
H12_MOUSE	Histone H1.2			-0.35	0.69	1.04	0.62

H13_MOUSE	Histone H1.3		0.73				
H14_MOUSE	Histone H1.4	-1.55					
H2A1_MOUSE	Histone H2A type 1					1.44	
H2A1F_MOUSE	Histone H2A type 1-F	-2.39			0.85		
H2A1H_MOUSE	Histone H2A type 1-H						0.71
H2A2A_MOUSE	Histone H2A type 2-A	-1.23	-0.17	-0.66		1.98	
H2A3_MOUSE	Histone H2A type 3			-0.45			
H2AY_MOUSE	Core histone macro-H2A.1			0.89			
H2B1F_MOUSE	Histone H2B type 1-F/J/L	0.62					
H2B1P_MOUSE	Histone H2B type 1-P		1.42	-0.31	1.13	2.47	0.87
H31_MOUSE	Histone H3.1	-9.97		-9.97		1.83	0.75
H33_MOUSE	Histone H3.3	-1.69	0.24	0.09	0.89		
H4_MOUSE	Histone H4	-2.51	-1.06	0.04	1.10	0.73	0.54
HACL1_MOUSE	2-hydroxyacyl-CoA lyase 1	-3.32	-2.63	0.34	-0.02		-1.46
HAOX1_MOUSE	Hydroxyacid oxidase 1	-3.28		-0.11	0.29	-2.49	-0.53
HBA_MOUSE	Hemoglobin subunit alpha	0.93	1.03	-0.56	-0.11	2.75	1.01
HBB1_MOUSE	Hemoglobin subunit beta-1	0.09	0.63	-0.39	-0.39	1.83	0.42
HCD2_MOUSE	3-hydroxyacyl-CoA dehydrogenase type-2	-1.03	-0.01	0.26	0.92	1.07	0.26
HCDH_MOUSE	Hydroxyacyl-coenzyme A dehydrogenase, mitochondrial	-0.66	-0.41	0.12	-0.06	0.04	-0.48
HDHD3_MOUSE	Haloacid dehalogenase-like hydrolase domain-containing protein 3	-0.37					
HEBP1_MOUSE	Heme-binding protein 1	-0.76		0.45		-0.46	-0.04
HEM2_MOUSE	Delta-aminolevulinic acid dehydratase	-1.13	-1.01	-0.08	0.09	-0.32	0.15
HEM6_MOUSE	Coproporphyrinogen-III oxidase, mitochondrial	-1.20	-1.63	-0.94	0.11		
HEMO_MOUSE	Hemopexin	1.96	2.82	1.90	1.26	1.72	-1.06
HGD_MOUSE	Homogentisate 1,2-dioxygenase	-1.48	-0.58	0.38	-0.28	-0.43	-0.39
HIBCH_MOUSE	3-hydroxyisobutyryl-CoA hydrolase, mitochondrial	-2.61	1.58	0.09			
HINT1_MOUSE	Histidine triad nucleotide-binding protein 1	1.65	0.87	0.14	0.31	0.52	0.34
HINT2_MOUSE	Histidine triad nucleotide-binding protein 2, mitochondrial	-1.84	-0.70	-0.28	-0.22		-0.74
HIUH_MOUSE	5-hydroxyisourate hydrolase	-0.47	-0.24	0.88	1.47	0.29	-0.25
HMCS1_MOUSE	Hydroxymethylglutaryl-CoA synthase, cytoplasmic			0.67			
HMCS2_MOUSE	Hydroxymethylglutaryl-CoA synthase, mitochondrial	-0.82	-0.34	0.37	0.42	0.38	-0.92
HMGB1_MOUSE	High mobility group protein B1						4.84
HMGCL_MOUSE	Hydroxymethylglutaryl-CoA lyase, mitochondrial	-0.64	-0.11	0.18	0.76	1.54	-0.79

HNRDL_MOUSE	Heterogeneous nuclear ribonucleoprotein D-like			0.13	0.44		
HNRL2_MOUSE	Heterogeneous nuclear ribonucleoprotein U-like protein 2		-1.62	-0.75			
HNRPC_MOUSE	Heterogeneous nuclear ribonucleoproteins C1/C2	3.65	3.26	0.44			
HNRPD_MOUSE	Heterogeneous nuclear ribonucleoprotein D0	1.16	1.55			0.80	-0.04
HNRPF_MOUSE	Heterogeneous nuclear ribonucleoprotein F	0.36	1.57	-0.04	1.35	-0.09	0.34
HNRPK_MOUSE	Heterogeneous nuclear ribonucleoprotein K	1.62	0.95	0.27	0.28	0.04	0.34
HNRPL_MOUSE	Heterogeneous nuclear ribonucleoprotein L	0.17				-0.16	
HNRPM_MOUSE	Heterogeneous nuclear ribonucleoprotein M		2.78				
HNRPQ_MOUSE	Heterogeneous nuclear ribonucleoprotein Q	0.27	0.47	0.72		0.75	0.31
HOGA1_MOUSE	Probable 4-hydroxy-2-oxoglutarate aldolase, mitochondrial	-1.68	-1.10	-0.15	0.47	-0.15	-0.42
HPPD_MOUSE	4-hydroxyphenylpyruvate dioxygenase	-0.89	-0.58	0.45	0.49	0.43	-1.04
HPRT_MOUSE	Hypoxanthine-guanine phosphoribosyltransferase			0.51			-4.88
HPT_MOUSE	Haptoglobin	2.63	2.47	2.13	2.13	1.69	-0.82
HS90A_MOUSE	Heat shock protein HSP 90-alpha	-0.30	-0.99	0.18	0.50	-0.27	
HS90B_MOUSE	Heat shock protein HSP 90-beta	-0.81	-1.31	0.19	0.54	0.20	0.50
HSP7C_MOUSE	Heat shock cognate 71 kDa protein	0.14	0.40	0.02	0.56	0.22	0.25
HSPB1_MOUSE	Heat shock protein beta-1	0.92					
HUTH_MOUSE	Histidine ammonia-lyase	-1.01	-2.59	0.35	0.38	-0.01	
HUTI_MOUSE	Probable imidazolonepropionase			0.44	0.38		
HUTU_MOUSE	Urocanate hydratase	-0.77	-1.19	0.45	0.32	-0.19	-0.91
HYEP_MOUSE	Epoxide hydrolase 1	-4.80			0.20	-1.18	
HYES_MOUSE	Epoxide hydrolase 2	-1.99	-1.74	0.04	0.10	0.36	-0.11
HYOU1_MOUSE	Hypoxia up-regulated protein 1	0.76	0.78	1.48	0.66	0.07	1.05
IAH1_MOUSE	Isoamyl acetate-hydrolyzing esterase 1 homolog		-0.93	0.54	0.58	1.42	-1.22
ICA_MOUSE	Inhibitor of carbonic anhydrase	4.18					
IDHC_MOUSE	Isocitrate dehydrogenase [NADP] cytoplasmic	-1.19	-0.54	0.40	0.52	0.30	-0.11
IDHP_MOUSE	Isocitrate dehydrogenase [NADP], mitochondrial	-2.11	-1.41	0.21	0.22	-0.19	-0.42
IDI1_MOUSE	Isopentenyl-diphosphate Delta-isomerase 1				1.75		-1.38
IF_MOUSE	Gastric intrinsic factor			0.12			
IF2A_MOUSE	Eukaryotic translation initiation factor 2 subunit 1					-1.64	
IF4A1_MOUSE	Eukaryotic initiation factor 4A-I	0.29		0.47	0.58	-0.08	-0.81
IF4G1_MOUSE	Eukaryotic translation initiation factor 4 gamma 1				0.11	0.77	
IF5A1_MOUSE	Eukaryotic translation initiation factor 5A-1	1.65	1.58	-0.09	0.03	0.78	-0.15
IIGP1_MOUSE	Interferon-inducible GTPase 1	0.49			-0.12		

IMB1_MOUSE	Importin subunit beta-1					-1.24	1.09
IMMT_MOUSE	Mitochondrial inner membrane protein	1.41	0.45				
INMT_MOUSE	Indolethylamine N-methyltransferase	1.50	0.75	0.86	1.26	1.64	-1.80
IPYR_MOUSE	Inorganic pyrophosphatase	1.74	2.02	0.36	0.55	1.03	-0.27
IPYR2_MOUSE	Inorganic pyrophosphatase 2, mitochondrial			0.69			
ISC2A_MOUSE	Isochorismatase domain-containing protein 2A, mitochondrial	-1.52	-0.77	-0.43	0.22	0.38	-0.06
ITIH3_MOUSE	Inter-alpha-trypsin inhibitor heavy chain H3		3.33		1.94		-5.06
IVD_MOUSE	Isovaleryl-CoA dehydrogenase, mitochondrial	-1.46	-0.71	0.02	-0.42	0.49	-0.89
K1C18_MOUSE	Keratin, type I cytoskeletal 18	2.53	2.12	-0.90	0.32	0.39	0.07
K2C8_MOUSE	Keratin, type II cytoskeletal 8	2.08	1.84	-0.59	0.27	0.55	0.01
KAD2_MOUSE	Adenylate kinase 2, mitochondrial	1.06	1.88	0.12	-0.09	0.50	-0.35
KAD3_MOUSE	GTP:AMP phosphotransferase, mitochondrial	-2.23	-1.31	0.13	0.35		-0.30
KBTB3_MOUSE	Kelch repeat and BTB domain-containing protein 3	2.34	2.81				
KCNQ4_MOUSE	Potassium voltage-gated channel subfamily KQT member 4				-0.16		
KCY_MOUSE	UMP-CMP kinase			-0.72	1.15		-1.58
KEG1_MOUSE	Glycine N-acyltransferase-like protein Keg1	-2.52	-1.76	0.63	-0.46		
KHK_MOUSE	Ketohexokinase	0.39	-0.22	0.42	1.02	-0.31	-0.98
KLC2_MOUSE	Kinesin light chain 2		0.47				
KMO_MOUSE	Kynurenine 3-monooxygenase	-9.97	-2.65		0.20	-0.32	
KNG1_MOUSE	Kininogen-1	0.27	0.67	0.46	0.23	0.19	-1.63
KPYM_MOUSE	Pyruvate kinase isozymes M1/M2		-0.32	-0.21	0.74		
LA_MOUSE	Lupus La protein homolog					-9.97	-0.49
LACTB_MOUSE	Serine beta-lactamase-like protein LACTB, mitochondrial	-1.95		-0.61	0.07	-0.23	
LASP1_MOUSE	LIM and SH3 domain protein 1		2.77				
LDB1_MOUSE	LIM domain-binding protein 1						-0.25
LDHA_MOUSE	L-lactate dehydrogenase A chain	-1.14	-0.50	0.88	0.99	0.12	-0.75
LDHD_MOUSE	Probable D-lactate dehydrogenase, mitochondrial	-1.47	-1.05	-0.63			-0.42
LEG9_MOUSE	Galectin-9				0.11		
LETM1_MOUSE	LETM1 and EF-hand domain-containing protein 1, mitochondrial		0.57				
LETM2_MOUSE	LETM1 domain-containing protein LETM2, mitochondrial				-0.18		
LGMN_MOUSE	Legumain			0.36			-1.54

LGUL_MOUSE	Lactoylglutathione lyase	0.55	0.87	0.48	-0.11	0.67	-0.54
LIMA1_MOUSE	LIM domain and actin-binding protein 1						-4.80
LKHA4_MOUSE	Leukotriene A-4 hydrolase	-0.74					
LMAN1_MOUSE	Protein ERGIC-53	1.87					0.75
LMNA_MOUSE	Prelamin-A/C	1.79	2.21				
LONM_MOUSE	Lon protease homolog, mitochondrial		-0.66	-2.88	0.96		
LPPRC_MOUSE	Leucine-rich PPR motif-containing protein, mitochondrial					3.36	
LRC59_MOUSE	Leucine-rich repeat-containing protein 59	-1.18	-1.22	1.18	-0.08	-0.87	1.33
LRIG1_MOUSE	Leucine-rich repeats and immunoglobulin-like domains protein 1				-0.07		
LRRK2_MOUSE	Leucine-rich repeat serine/threonine-protein kinase 2					-1.81	
LYPA1_MOUSE	Acyl-protein thioesterase 1	-1.16	-0.08	0.39	0.49	0.49	-0.33
M2GD_MOUSE	Dimethylglycine dehydrogenase, mitochondrial	-1.83	-1.33	0.00	-0.19	-0.69	-0.93
MAAI_MOUSE	Maleylacetoacetate isomerase	1.35	0.31	0.45	0.17	-0.28	-0.60
MANF_MOUSE	Mesencephalic astrocyte-derived neurotrophic factor	3.44	2.95				
MAOX_MOUSE	NADP-dependent malic enzyme		-9.97		0.50		
MCCA_MOUSE	Methylcrotonoyl-CoA carboxylase subunit alpha, mitochondrial		-2.77				
MCCB_MOUSE	Methylcrotonoyl-CoA carboxylase beta chain, mitochondrial	-2.24					
MCE1_MOUSE	mRNA-capping enzyme					-1.04	1.57
MDHC_MOUSE	Malate dehydrogenase, cytoplasmic	-0.61	-0.35	0.57	0.22	0.48	-0.53
MDHM_MOUSE	Malate dehydrogenase, mitochondrial	-1.40	-0.90	0.03	-0.18	-0.45	-0.44
MET7B_MOUSE	Methyltransferase-like protein 7B	-2.53			0.34	-0.91	0.10
METK1_MOUSE	S-adenosylmethionine synthase isoform type-1	-0.81	-0.35	0.56	0.32	0.39	-1.31
MGLL_MOUSE	Monoglyceride lipase				-0.11		
MGST1_MOUSE	Microsomal glutathione S-transferase 1	-1.34	-2.72	0.27	-0.17	-0.65	-0.93
MICA3_MOUSE	Protein MICAL-3					1.22	-9.97
MIF_MOUSE	Macrophage migration inhibitory factor		-0.37	-0.35	0.19	-0.44	-0.32
ML12B_MOUSE	Myosin regulatory light chain 12B	3.59					
MMSA_MOUSE	Methylmalonate-semialdehyde dehydrogenase [acylating], mitochondrial	-1.90	-1.27	0.54	0.26	0.17	-0.76
MOSC1_MOUSE	MOSC domain-containing protein 1, mitochondrial				0.68		
MOSC2_MOUSE	MOSC domain-containing protein 2, mitochondrial	-2.56	-3.16		-0.11	0.27	0.20
MPCP_MOUSE	Phosphate carrier protein, mitochondrial	-3.11	-5.32		0.08	-0.57	0.19

MPPA_MOUSE	Mitochondrial-processing peptidase subunit alpha			0.18			
MT1_MOUSE	Metallothionein-1			0.27			-0.78
MT2_MOUSE	Metallothionein-2	3.91	3.00	0.56	-0.13		-0.45
MTAP_MOUSE	S-methyl-5'-thioadenosine phosphorylase				-1.00		
MTCH2_MOUSE	Mitochondrial carrier homolog 2	-4.80	-2.52		-0.66	-0.52	
MTP_MOUSE	Microsomal triglyceride transfer protein large subunit	-2.36	-1.88	0.58	0.53	-0.22	0.27
MTPN_MOUSE	Myotrophin		0.72			1.92	
MTSS1_MOUSE	Metastasis suppressor protein 1			-5.32			
MUG1_MOUSE	Murinoglobulin-1	-1.61	-1.28	-0.49	-0.30	5.17	
MUP1_MOUSE	Major urinary protein 1	1.10	1.57	0.77	-0.79	2.01	-1.88
MUP2_MOUSE	Major urinary protein 2	1.56	1.97	0.77	-0.27	1.72	-1.60
MYH9_MOUSE	Myosin-9	3.17	2.04	0.83	1.26	1.62	0.04
MYL6_MOUSE	Myosin light polypeptide 6	1.96	2.67	0.08	1.04	1.60	-0.28
MYPC2_MOUSE	Myosin-binding protein C, fast-type			-0.83			
NAA16_MOUSE	N-alpha-acetyltransferase 16, NatA auxiliary subunit				0.32		
NACAM_MOUSE	Nascent polypeptide-associated complex subunit alpha, muscle-specific form		-3.57				
NADC_MOUSE	Nicotinate-nucleotide pyrophosphorylase [carboxylating]		-1.13	0.16	-0.04	0.43	1.02
NAKD1_MOUSE	NAD kinase domain-containing protein 1			0.57	0.09	-0.44	
NB5R3_MOUSE	NADH-cytochrome b5 reductase 3		-3.07	-0.50	-0.29	-1.14	
NBEA_MOUSE	Neurobeachin				0.09		
NCPR_MOUSE	NADPH--cytochrome P450 reductase				0.22	-0.58	-0.36
NDKA_MOUSE	Nucleoside diphosphate kinase A	-0.45	0.67	0.27	0.91	1.59	-0.33
NDKB_MOUSE	Nucleoside diphosphate kinase B	0.52	0.68	0.71	0.50	0.84	0.06
NDRG2_MOUSE	Protein NDRG2		-0.48		-0.41	0.35	-0.12
NDUA2_MOUSE	NADH dehydrogenase [ubiquinone] 1 alpha subcomplex subunit 2	-2.37	-1.96				
NDUA4_MOUSE	NADH dehydrogenase [ubiquinone] 1 alpha subcomplex subunit 4		-1.98		0.74	0.03	-0.17
NDUA5_MOUSE	NADH dehydrogenase [ubiquinone] 1 alpha subcomplex subunit 5	0.65	1.46	0.48			
NDUA6_MOUSE	NADH dehydrogenase [ubiquinone] 1 alpha subcomplex subunit 6			-0.02			
NDUAA_MOUSE	NADH dehydrogenase [ubiquinone] 1 alpha subcomplex subunit 10, mitochondrial	-1.99	-1.38	0.62	1.07	1.00	-1.23

NDUBA_MOUSE	NADH dehydrogenase [ubiquinone] 1 beta subcomplex subunit 10	0.25	0.41		0.15		
NDUS1_MOUSE	NADH-ubiquinone oxidoreductase 75 kDa subunit, mitochondrial				-0.13	-0.78	
NDUS4_MOUSE	NADH dehydrogenase [ubiquinone] iron-sulfur protein 4, mitochondrial	2.44	4.09				
NDUS5_MOUSE	NADH dehydrogenase [ubiquinone] iron-sulfur protein 5	-1.65	-1.09		-0.15		
NDUS6_MOUSE	NADH dehydrogenase [ubiquinone] iron-sulfur protein 6, mitochondrial	1.87	2.40	0.17		0.29	-0.37
NDUS8_MOUSE	NADH dehydrogenase [ubiquinone] iron-sulfur protein 8, mitochondrial		-0.16		-0.01		
NDUV1_MOUSE	NADH dehydrogenase [ubiquinone] flavoprotein 1, mitochondrial	-4.11	-2.72	-0.32	0.01	-1.98	-1.47
NDUV2_MOUSE	NADH dehydrogenase [ubiquinone] flavoprotein 2, mitochondrial	0.62	1.78	-0.53	-2.76	0.79	-1.70
NEDD8_MOUSE	NEDD8	2.58	1.02	0.64		1.86	0.10
NF1_MOUSE	Neurofibromin	-1.98	-2.10		-0.03	-0.24	
NGAL_MOUSE	Neutrophil gelatinase-associated lipocalin		4.35				
NHRF1_MOUSE	Na(+)/H(+) exchange regulatory cofactor NHE-RF1	-0.41					
NIBL1_MOUSE	Niban-like protein 1	-0.88					
NIN_MOUSE	Ninein	-9.97		0.95			
NIPS1_MOUSE	Protein NipSnap homolog 1	-1.60	-1.45	0.05	0.01	-0.54	-1.60
NIT1_MOUSE	Nitrilase homolog 1	-0.32	-0.55	0.07	0.70		
NIT2_MOUSE	Omega-amidase NIT2	0.51	0.44	0.25	0.33	0.01	-0.89
NLTP_MOUSE	Non-specific lipid-transfer protein	0.68	0.60	0.36	-0.12	0.55	-0.29
NNTM_MOUSE	NAD(P) transhydrogenase, mitochondrial	-1.10				-1.06	
NP1L1_MOUSE	Nucleosome assembly protein 1-like 1		0.21	0.41	0.94	0.24	-0.28
NPHP3_MOUSE	Nephrocystin-3			0.02		0.19	
NPM_MOUSE	Nucleophosmin	2.99	2.01				2.14
NPS3A_MOUSE	Protein NipSnap homolog 3A	2.12	6.01	0.09		4.78	
NQO2_MOUSE	Ribosyldihydronicotinamide dehydrogenase [quinone]			0.73			-1.12
NUCB1_MOUSE	Nucleobindin-1	1.15	1.43	1.14	-0.12	0.02	-0.09
NUCL_MOUSE	Nucleolin	0.85	1.24	0.41	1.08	-0.31	4.64
NUDT7_MOUSE	Peroxisomal coenzyme A diphosphatase NUDT7	-1.19	-0.61	1.27	-0.34	0.38	-0.80
NXP20_MOUSE	Protein Noxp20					-1.37	

OAT_MOUSE	Ornithine aminotransferase, mitochondrial	-0.64	0.01	-0.33	0.65	-1.08	-2.92
OBSCN_MOUSE	Obscurin	1.65	2.54				
OCTC_MOUSE	Peroxisomal carnitine O-octanoyltransferase				1.30		
ODB2_MOUSE	Lipoamide acyltransferase component of branched-chain alpha-keto acid dehydrogenase complex, mitochondrial	-4.44	6.51	0.76	-0.10	1.04	0.20
ODBA_MOUSE	2-oxoisovalerate dehydrogenase subunit alpha, mitochondrial	-2.53	-1.47	0.53	-0.79		
ODBB_MOUSE	2-oxoisovalerate dehydrogenase subunit beta, mitochondrial	-3.92		-0.16	-0.83		
ODO1_MOUSE	2-oxoglutarate dehydrogenase, mitochondrial	-4.13	-2.98	0.14	0.39	-0.07	0.33
ODO2_MOUSE	Dihydrolipoyllysine-residue succinyltransferase component of 2-oxoglutarate dehydrogenase complex, mitochondrial	-1.09	0.09	0.09	0.06	0.27	-0.48
ODP2_MOUSE	Dihydrolipoyllysine-residue acetyltransferase component of pyruvate dehydrogenase complex, mitochondrial	-1.92	-1.51				
ODPA_MOUSE	Pyruvate dehydrogenase E1 component subunit alpha, somatic form, mitochondrial	-2.97	-0.90				
ODPB_MOUSE	Pyruvate dehydrogenase E1 component subunit beta, mitochondrial	-1.87	0.83	1.47			-0.77
OLA1_MOUSE	Obg-like ATPase 1				-0.37	-0.74	-0.50
OPLA_MOUSE	5-oxoprolinase	-1.85			0.93	-0.14	-0.12
OST48_MOUSE	Dolichyl-diphosphooligosaccharide--protein glycosyltransferase 48 kDa subunit	-0.19	-0.43		0.64		0.48
OSTC_MOUSE	Oligosaccharyltransferase complex subunit OSTC				-0.10		
OTC_MOUSE	Ornithine carbamoyltransferase, mitochondrial	-0.95	-0.22	0.04	-0.18	-0.20	-0.83
P85A_MOUSE	Phosphatidylinositol 3-kinase regulatory subunit alpha						0.63
PA1B2_MOUSE	Platelet-activating factor acetylhydrolase IB subunit beta						-0.35
PA2G4_MOUSE	Proliferation-associated protein 2G4	-1.85					
PABP1_MOUSE	Polyadenylate-binding protein 1	1.12	0.69	0.02	0.46	-0.44	0.47
PAHX_MOUSE	Phytanoyl-CoA dioxygenase, peroxisomal	-1.81		-0.53			-0.43
PAIRB_MOUSE	Plasminogen activator inhibitor 1 RNA-binding protein	3.49	3.05	0.28		1.25	0.34
PARK7_MOUSE	Protein DJ-1	0.64	0.66	0.23	0.38	0.76	-0.71
PBLD1_MOUSE	Phenazine biosynthesis-like domain-containing protein	-0.13	-0.86		0.26	0.49	0.60

	1						
PBLD2_MOUSE	Phenazine biosynthesis-like domain-containing protein 2			0.36			
PCBP1_MOUSE	Poly(rC)-binding protein 1	-0.63	-0.47	0.52	0.59	0.04	0.10
PCBP2_MOUSE	Poly(rC)-binding protein 2			0.18			0.26
PCCA_MOUSE	Propionyl-CoA carboxylase alpha chain, mitochondrial		-1.19	0.17	-0.43	-0.94	
PCCB_MOUSE	Propionyl-CoA carboxylase beta chain, mitochondrial	-1.95	-1.34	-0.07	-0.23		-1.61
PCKGC_MOUSE	Phosphoenolpyruvate carboxykinase, cytosolic [GTP]	-0.12	-0.09	0.62	-0.55	0.52	-1.56
PCTL_MOUSE	PCTP-like protein			1.24			0.14
PDIA1_MOUSE	Protein disulfide-isomerase	1.28	0.99	0.93	0.83	0.64	-0.16
PDIA3_MOUSE	Protein disulfide-isomerase A3	0.86	0.38	0.81	0.47	0.10	0.14
PDIA4_MOUSE	Protein disulfide-isomerase A4	0.44	0.22	1.14	0.77	0.50	0.70
PDIA6_MOUSE	Protein disulfide-isomerase A6	-0.75	-0.90	1.03	0.44	-0.15	0.06
PDS5B_MOUSE	Sister chromatid cohesion protein PDS5 homolog B				-0.35		
PEBP1_MOUSE	Phosphatidylethanolamine-binding protein 1	1.15	0.70	0.70	0.62	-0.17	-0.07
PECR_MOUSE	Peroxisomal trans-2-enoyl-CoA reductase	-1.14	-0.49	0.26	0.04		-0.58
PGAM1_MOUSE	Phosphoglycerate mutase 1	-0.43	-0.55	0.28	0.29	0.19	-0.24
PGK1_MOUSE	Phosphoglycerate kinase 1	0.41	-0.18	0.26	0.15	0.40	0.09
PGM1_MOUSE	Phosphoglucomutase-1	-2.34	-0.69	0.71	0.70	0.02	-1.22
PGRC1_MOUSE	Membrane-associated progesterone receptor component 1	0.23	-0.90	0.94	0.32	-0.41	-0.14
PH4H_MOUSE	Phenylalanine-4-hydroxylase	-2.44	-2.91	0.78	0.16	-0.48	0.27
PHAR1_MOUSE	Phosphatase and actin regulator 1				4.22		
PHB_MOUSE	Prohibitin	1.09	1.05		0.73		
PHB2_MOUSE	Prohibitin-2		-1.87		0.46		
PHS_MOUSE	Pterin-4-alpha-carbinolamine dehydratase	0.29	-0.17	0.88	-0.34	0.40	0.15
PLCB1_MOUSE	1-phosphatidylinositol-4,5-bisphosphate phosphodiesterase beta-1		1.92				
PLF4_MOUSE	Platelet factor 4			-0.10			
PLIN2_MOUSE	Perilipin-2			-3.22			
PLMN_MOUSE	Plasminogen	2.01	1.75	0.44	0.49	0.00	-1.22
PLPL7_MOUSE	Patatin-like phospholipase domain-containing protein 7	2.40	2.46			1.05	
PLSL_MOUSE	Plastin-2			0.37		1.76	
PLST_MOUSE	Plastin-3			0.18	1.26		
PMGE_MOUSE	Bisphosphoglycerate mutase						0.65
PNPH_MOUSE	Purine nucleoside phosphorylase	1.20	3.99	0.29	2.26	1.64	

PNPO_MOUSE	Pyridoxine-5'-phosphate oxidase			0.48			-0.15
PON1_MOUSE	Serum paraoxonase/arylesterase 1				-0.95	-0.30	
PPHLN_MOUSE	Periphilin-1				-0.22		
PPIA_MOUSE	Peptidyl-prolyl cis-trans isomerase A	1.33	1.32	0.27	0.83	1.09	0.10
PPIB_MOUSE	Peptidyl-prolyl cis-trans isomerase B	1.29	0.99	0.94	0.90	0.79	0.57
PPIF_MOUSE	Peptidyl-prolyl cis-trans isomerase F, mitochondrial	0.20		-0.03			
PPT1_MOUSE	Palmitoyl-protein thioesterase 1			0.04			
PRDX1_MOUSE	Peroxiredoxin-1	0.85	0.89	0.32	0.99	0.99	-0.46
PRDX2_MOUSE	Peroxiredoxin-2	1.92	2.20	-0.03	0.35	1.91	-0.40
PRDX3_MOUSE	Thioredoxin-dependent peroxide reductase, mitochondrial	0.82	1.90	-0.10	0.55	-0.29	-1.88
PRDX4_MOUSE	Peroxiredoxin-4				1.73		
PRDX5_MOUSE	Peroxiredoxin-5, mitochondrial	0.32	0.65	0.25	0.09	0.28	-0.36
PRDX6_MOUSE	Peroxiredoxin-6	0.40	0.17	0.58	0.46	0.45	-0.02
PROF1_MOUSE	Profilin-1	0.20	0.39	0.16	0.69	0.62	0.01
PROSC_MOUSE	Proline synthase co-transcribed bacterial homolog protein			0.14			
PRS10_MOUSE	26S protease regulatory subunit 10B					1.41	
PRS7_MOUSE	26S protease regulatory subunit 7	0.07	0.33				
PRS8_MOUSE	26S protease regulatory subunit 8				-0.23		
PSA1_MOUSE	Proteasome subunit alpha type-1	-0.44					
PSA2_MOUSE	Proteasome subunit alpha type-2			-0.08			0.86
PSA3_MOUSE	Proteasome subunit alpha type-3						0.05
PSA6_MOUSE	Proteasome subunit alpha type-6	0.96	-0.82	0.55	0.75	0.03	-0.84
PSB1_MOUSE	Proteasome subunit beta type-1					2.13	
PSB2_MOUSE	Proteasome subunit beta type-2				0.86	-0.92	
PSD13_MOUSE	26S proteasome non-ATPase regulatory subunit 13				0.26	1.15	
PSD7_MOUSE	26S proteasome non-ATPase regulatory subunit 7			0.10	0.19	-1.71	-1.38
PSMD4_MOUSE	26S proteasome non-ATPase regulatory subunit 4			0.33			
PSMD6_MOUSE	26S proteasome non-ATPase regulatory subunit 6		-1.21				
PSME1_MOUSE	Proteasome activator complex subunit 1	-0.55	-1.15	0.39		0.23	
PSME2_MOUSE	Proteasome activator complex subunit 2			0.25		0.93	
PSME3_MOUSE	Proteasome activator complex subunit 3	-9.97	3.74				
PTMA_MOUSE	Prothymosin alpha	2.27	2.71	0.16		-1.04	1.56
PTMS_MOUSE	Parathymosin	1.14	1.70	0.87		0.33	0.68
PUR6_MOUSE	Multifunctional protein ADE2				0.22		

PYC_MOUSE	Pyruvate carboxylase, mitochondrial	-2.11	-1.75	0.54	0.12	0.02	-0.18
PYGL_MOUSE	Glycogen phosphorylase, liver form	3.00	-1.47	0.51	-0.08	-0.74	-0.84
QCR1_MOUSE	Cytochrome b-c1 complex subunit 1, mitochondrial	-1.30	-0.16	-0.08	-0.04	0.09	-0.42
QCR2_MOUSE	Cytochrome b-c1 complex subunit 2, mitochondrial	-1.16	-0.54	0.24	0.43	0.85	-0.49
QCR6_MOUSE	Cytochrome b-c1 complex subunit 6, mitochondrial	2.42	3.20	0.79	0.49	0.65	-0.13
QCR7_MOUSE	Cytochrome b-c1 complex subunit 7	0.38	2.39	-0.48			
QCR8_MOUSE	Cytochrome b-c1 complex subunit 8	-4.04	-4.44		0.07		
RAB10_MOUSE	Ras-related protein Rab-10	-2.25		0.49	0.10		0.25
RAB1B_MOUSE	Ras-related protein Rab-1B			0.44		-1.89	
RAB26_MOUSE	Ras-related protein Rab-26		1.34				
RAB7A_MOUSE	Ras-related protein Rab-7a		-0.67				-0.60
RADI_MOUSE	Radixin	0.85	0.63				
RAI2_MOUSE	Retinoic acid-induced protein 2	-1.35					
RAN_MOUSE	GTP-binding nuclear protein Ran			0.19	0.68	-0.59	0.16
RAP1A_MOUSE	Ras-related protein Rap-1A			0.27	0.41		-1.22
RAP1B_MOUSE	Ras-related protein Rap-1b					0.23	
RASL2_MOUSE	Ras GTPase-activating protein 4	-3.02					
RBM3_MOUSE	Putative RNA-binding protein 3	5.61	5.75	0.00		1.55	0.26
RBMX_MOUSE	RNA-binding motif protein, X chromosome		2.67				
RD23B_MOUSE	UV excision repair protein RAD23 homolog B	3.29					
RDH7_MOUSE	Retinol dehydrogenase 7		-3.59		0.81	-0.28	
REEP6_MOUSE	Receptor expression-enhancing protein 6		-2.63		0.33	-1.19	
RET4_MOUSE	Retinol-binding protein 4						-2.72
RFA1_MOUSE	Replication protein A 70 kDa DNA-binding subunit				-9.97	8.30	
RGN_MOUSE	Regucalcin	0.54	1.05	1.10	1.21	0.63	-0.18
RHG21_MOUSE	Rho GTPase-activating protein 21			-0.08			
RHOA_MOUSE	Transforming protein RhoA			-1.04			0.26
RHOC_MOUSE	Rho-related GTP-binding protein RhoC					-0.39	
RL10A_MOUSE	60S ribosomal protein L10a	-3.01	-1.63	1.14	0.21	-0.49	0.30
RL10L_MOUSE	60S ribosomal protein L10-like					-0.87	
RL11_MOUSE	60S ribosomal protein L11	-2.80	-1.92	0.62	0.42	0.09	-0.06
RL12_MOUSE	60S ribosomal protein L12	-0.92	-0.83	0.23	0.36	0.15	0.28
RL13_MOUSE	60S ribosomal protein L13	-3.17	-2.33	0.29	0.37	-0.33	0.41
RL13A_MOUSE	60S ribosomal protein L13a	-4.08			-0.03	-0.54	0.07
RL14_MOUSE	60S ribosomal protein L14	-2.83	-1.78	0.55	0.21	-0.36	0.49
RL15_MOUSE	60S ribosomal protein L15	-3.92	-2.44	0.94	0.20	-0.48	0.07

RL17_MOUSE	60S ribosomal protein L17		-3.61	0.00	0.72	0.13	-0.18
RL18_MOUSE	60S ribosomal protein L18	-2.67	-2.56	0.76	0.33	-0.91	0.51
RL18A_MOUSE	60S ribosomal protein L18a	-2.15		0.55	0.63	-0.20	0.36
RL19_MOUSE	60S ribosomal protein L19	-2.74	-1.13	0.51	0.13	-2.11	0.09
RL21_MOUSE	60S ribosomal protein L21	-1.25			0.30	-0.02	0.49
RL22_MOUSE	60S ribosomal protein L22	-0.40	0.08	0.77		0.64	-0.40
RL23_MOUSE	60S ribosomal protein L23	-3.54	-3.10	0.70	0.18	-0.44	0.55
RL23A_MOUSE	60S ribosomal protein L23a	-0.72	-0.71	0.04	0.04	-0.17	-0.17
RL24_MOUSE	60S ribosomal protein L24	-4.35	-5.06	0.32	0.22	-0.83	0.87
RL26_MOUSE	60S ribosomal protein L26	-2.27	-3.05	0.62	0.06	-0.30	0.03
RL27_MOUSE	60S ribosomal protein L27	-2.02	-1.68	0.42	0.53	-0.35	0.46
RL27A_MOUSE	60S ribosomal protein L27a	-1.54	-1.11	0.84	0.34	-0.67	0.15
RL28_MOUSE	60S ribosomal protein L28	-3.04	-1.46		-0.19	-0.39	0.36
RL29_MOUSE	60S ribosomal protein L29	-4.92	-3.72	0.41	0.25	-1.30	0.59
RL3_MOUSE	60S ribosomal protein L3	-3.38		1.46	0.65	-0.45	0.51
RL30_MOUSE	60S ribosomal protein L30	-1.79	-0.42	0.37	0.49	-0.01	-0.48
RL31_MOUSE	60S ribosomal protein L31	-0.08	0.12	0.33	0.34	1.05	-0.90
RL32_MOUSE	60S ribosomal protein L32	-3.59	-1.97	0.97	-0.12	-0.62	0.52
RL34_MOUSE	60S ribosomal protein L34	-1.63	-1.87	0.93	0.17	0.97	0.29
RL35_MOUSE	60S ribosomal protein L35	-0.61	-2.16	0.56	-0.18	-1.65	0.11
RL35A_MOUSE	60S ribosomal protein L35a					-0.22	-0.05
RL36_MOUSE	60S ribosomal protein L36	-2.88	-1.98			-1.47	0.50
RL36A_MOUSE	60S ribosomal protein L36a	-3.64	-2.79		0.56	-6.06	0.58
RL37A_MOUSE	60S ribosomal protein L37a	-3.99	-2.77				
RL38_MOUSE	60S ribosomal protein L38	-9.97			0.31	2.00	1.12
RL4_MOUSE	60S ribosomal protein L4	-2.59	-2.15	0.36	0.10	-0.78	0.31
RL5_MOUSE	60S ribosomal protein L5	-1.47	-1.74	0.39	0.19	-0.73	-0.22
RL6_MOUSE	60S ribosomal protein L6	-2.05	-1.60	0.53	0.91	-0.70	0.40
RL7_MOUSE	60S ribosomal protein L7	-3.99	-3.01	0.62	0.28	-1.38	0.26
RL7A_MOUSE	60S ribosomal protein L7a	-4.01	-3.35		0.03	-0.33	0.61
RL8_MOUSE	60S ribosomal protein L8	-2.98	-2.52	0.15	0.23	-0.56	0.80
RL9_MOUSE	60S ribosomal protein L9	-0.85	-0.81	0.61	0.37	0.46	-0.37
RLA0_MOUSE	60S acidic ribosomal protein P0	-2.63	-1.39	0.40	0.22	-0.04	0.25
RLA1_MOUSE	60S acidic ribosomal protein P1	1.37	0.73	0.18	0.40	0.26	0.25
RLA2_MOUSE	60S acidic ribosomal protein P2	1.42	1.86	0.35	-0.01	0.52	0.19
RM12_MOUSE	39S ribosomal protein L12, mitochondrial	2.32	2.54	1.48			

RMD1_MOUSE	Regulator of microtubule dynamics protein 1			-0.06			
RNAS4_MOUSE	Ribonuclease 4						-1.61
ROA1_MOUSE	Heterogeneous nuclear ribonucleoprotein A1	2.84	0.97	0.78	0.56	-0.27	-0.02
ROA2_MOUSE	Heterogeneous nuclear ribonucleoproteins A2/B1	1.48	1.76	0.32	0.19	0.37	0.07
ROA3_MOUSE	Heterogeneous nuclear ribonucleoprotein A3	1.39	2.51	-0.09	0.14	0.79	0.32
ROAA_MOUSE	Heterogeneous nuclear ribonucleoprotein A/B	1.89	2.86	0.31	0.70	0.66	0.47
RPN1_MOUSE	Dolichyl-diphosphooligosaccharide--protein glycosyltransferase subunit 1	0.35	-0.50		0.29	-0.22	1.92
RPN2_MOUSE	Dolichyl-diphosphooligosaccharide--protein glycosyltransferase subunit 2		4.25				
RRBP1_MOUSE	Ribosome-binding protein 1	1.56	1.57	0.50	-0.04	0.72	-0.13
RS10_MOUSE	40S ribosomal protein S10	-1.93	-1.88	0.63	0.39	-0.64	0.40
RS11_MOUSE	40S ribosomal protein S11	-3.34			0.30	-2.08	1.28
RS12_MOUSE	40S ribosomal protein S12	-2.00	-1.45	0.59	0.09	0.23	0.13
RS13_MOUSE	40S ribosomal protein S13	-2.77	-1.01	-0.18	0.31	-0.56	0.17
RS14_MOUSE	40S ribosomal protein S14	-1.56	-1.91	0.48	0.42	0.49	0.93
RS15_MOUSE	40S ribosomal protein S15	-1.67	-0.69	0.22	-0.63	-0.52	1.08
RS15A_MOUSE	40S ribosomal protein S15a		-1.85	1.04	0.31	-0.65	-1.30
RS16_MOUSE	40S ribosomal protein S16	-3.07	-2.13		0.54	-0.12	0.41
RS17_MOUSE	40S ribosomal protein S17	-0.40		0.45	0.66	1.06	0.03
RS18_MOUSE	40S ribosomal protein S18	-3.43	-2.77	1.09	0.40	-0.33	0.88
RS19_MOUSE	40S ribosomal protein S19	0.73	0.89	0.46	0.15	1.21	0.17
RS2_MOUSE	40S ribosomal protein S2	-3.11	-4.13	0.67	0.14	-0.71	0.58
RS20_MOUSE	40S ribosomal protein S20	-1.97	-2.08	0.64	0.39	0.23	0.30
RS21_MOUSE	40S ribosomal protein S21					0.21	
RS23_MOUSE	40S ribosomal protein S23	-9.97	-7.16		0.39	-0.89	0.75
RS24_MOUSE	40S ribosomal protein S24	-2.12	-1.49		0.44	-0.88	-0.12
RS25_MOUSE	40S ribosomal protein S25	-2.43		0.60	0.12	-0.36	0.33
RS26_MOUSE	40S ribosomal protein S26	-1.67	-2.04	0.36	-0.01	-0.92	0.25
RS27A_MOUSE	Ubiquitin-40S ribosomal protein S27a	3.38	4.28	0.73	0.82	2.96	-0.16
RS28_MOUSE	40S ribosomal protein S28	0.51	0.77	0.17	-0.23	-0.18	0.30
RS29_MOUSE	40S ribosomal protein S29	-9.97	-9.97	1.26	0.24	-2.37	0.61
RS3_MOUSE	40S ribosomal protein S3	-1.42	-0.55	0.60	0.48	-0.10	0.17
RS30_MOUSE	40S ribosomal protein S30		0.22	0.54		-0.46	0.23
RS3A_MOUSE	40S ribosomal protein S3a	-1.37	-0.55	0.50	0.56	0.11	1.99
RS4X_MOUSE	40S ribosomal protein S4, X isoform	-3.63	-3.97		0.39	-0.94	0.44

RS5_MOUSE	40S ribosomal protein S5	0.69				-0.83	0.08
RS6_MOUSE	40S ribosomal protein S6	-4.32	-9.97		0.05	-2.40	0.54
RS7_MOUSE	40S ribosomal protein S7	-1.09	-0.52	0.70	0.28	0.36	-0.30
RS8_MOUSE	40S ribosomal protein S8	-2.27	-2.56	0.31	-0.05	-0.86	-0.36
RS9_MOUSE	40S ribosomal protein S9	-2.94	-2.62		0.04	-0.53	0.22
RSSA_MOUSE	40S ribosomal protein SA	-0.61	-0.09	0.59	0.41	-0.11	-0.33
RTTN_MOUSE	Rotatin				0.34		
RYR1_MOUSE	Ryanodine receptor 1			0.05	-0.13		
S10A8_MOUSE	Protein S100-A8	3.33		1.13		-9.97	1.86
S10A9_MOUSE	Protein S100-A9	-0.83	-0.29	0.80	0.77	1.70	1.25
S10AB_MOUSE	Protein S100-A11	4.29	5.01	-0.09		-1.16	
S14L2_MOUSE	SEC14-like protein 2	-0.08	-0.21	0.85	-0.06	0.16	-0.44
S14L4_MOUSE	SEC14-like protein 4		-5.88				
S27A2_MOUSE	Very long-chain acyl-CoA synthetase	0.80	-2.89		-0.54	-0.58	
S27A5_MOUSE	Bile acyl-CoA synthetase	-5.06			-0.38	-0.27	-0.04
SAA1_MOUSE	Serum amyloid A-1 protein	0.58	1.99	3.53			
SAA2_MOUSE	Serum amyloid A-2 protein	1.80	2.40	5.06			
SAHH_MOUSE	Adenosylhomocysteinase	-0.78	-0.73	0.29	0.11	0.11	-0.54
SAMP_MOUSE	Serum amyloid P-component	1.69	4.21	-0.16	-1.03	1.47	-1.61
SAP_MOUSE	Sulfated glycoprotein 1	2.07	1.62	-0.13	0.89	0.94	0.06
SAR1A_MOUSE	GTP-binding protein SAR1a			0.82			
SAR1B_MOUSE	GTP-binding protein SAR1b	-0.35			0.55	0.18	
SARDH_MOUSE	Sarcosine dehydrogenase, mitochondrial	-1.97	-1.40	0.09	-0.32	-0.45	-0.96
SBP2_MOUSE	Selenium-binding protein 2	-0.14	0.69	0.45	-0.36	0.48	-0.77
SC23A_MOUSE	Protein transport protein Sec23A	-9.97	-1.37				
SC31A_MOUSE	Protein transport protein Sec31A		1.03				
SDHL_MOUSE	L-serine dehydratase/L-threonine deaminase	-0.64	-0.54	0.58	-0.94	-1.48	-2.08
SELB_MOUSE	Selenocysteine-specific elongation factor					0.59	
SET_MOUSE	Protein SET	4.04	3.34				
SFPQ_MOUSE	Splicing factor, proline- and glutamine-rich	2.99		-0.19			0.25
SFXN1_MOUSE	Sideroflexin-1		-2.90		0.07	-1.74	
SH3R1_MOUSE	E3 ubiquitin-protein ligase SH3RF1					-0.84	
SHE_MOUSE	SH2 domain-containing adapter protein E				5.66		
SMC4_MOUSE	Structural maintenance of chromosomes protein 4					0.02	
SMD3_MOUSE	Small nuclear ribonucleoprotein Sm D3		1.22	-0.38	2.57		
SND1_MOUSE	Staphylococcal nuclease domain-containing protein 1		-3.66				

SNP29_MOUSE	Synaptosomal-associated protein 29	0.44	0.45				
SNX25_MOUSE	Sorting nexin-25	-3.00					
SODC_MOUSE	Superoxide dismutase [Cu-Zn]	1.78	1.53	0.31	0.24	0.76	-0.32
SODM_MOUSE	Superoxide dismutase [Mn], mitochondrial	-1.06	-0.57	0.03	-0.02	-0.30	-1.12
SOX_MOUSE	Peroxisomal sarcosine oxidase	-1.47	-0.62	0.09	0.40	0.01	-1.00
SPA3K_MOUSE	Serine protease inhibitor A3K	0.73	0.92	0.19	-0.15	0.29	-2.42
SPA3M_MOUSE	Serine protease inhibitor A3M	-0.83	0.41				
SPA3N_MOUSE	Serine protease inhibitor A3N	-4.32					
SPEB_MOUSE	Agmatinase, mitochondrial	-1.28	-0.95				
SPEE_MOUSE	Spermidine synthase						0.70
SPRE_MOUSE	Sepiapterin reductase	-1.02	0.91	1.17	0.76		
SPS2_MOUSE	Selenide, water dikinase 2	-1.59	-1.43			1.23	
SPT5H_MOUSE	Transcription elongation factor SPT5					-5.21	
SPTA2_MOUSE	Spectrin alpha chain, brain	3.95			-0.24		
SPYA_MOUSE	Serine--pyruvate aminotransferase, mitochondrial	-1.74	-1.80	0.60	-0.03	0.14	-0.70
SSDH_MOUSE	Succinate-semialdehyde dehydrogenase, mitochondrial	-2.20	-1.80				
SSRA_MOUSE	Translocon-associated protein subunit alpha		1.00				
ST1A1_MOUSE	Sulfotransferase 1A1			0.34	-0.23	0.62	-0.39
STAG1_MOUSE	Cohesin subunit SA-1	0.26	0.51				
STIP1_MOUSE	Stress-induced-phosphoprotein 1	1.52	0.30				
STMN2_MOUSE	Stathmin-2						3.14
SUCA_MOUSE	Succinyl-CoA ligase [ADP/GDP-forming] subunit alpha, mitochondrial	-2.16	-1.62	0.88	-0.25	0.50	-0.26
SUCB1_MOUSE	Succinyl-CoA ligase [ADP-forming] subunit beta, mitochondrial	-2.43	-1.60	1.35	0.15		
SUCB2_MOUSE	Succinyl-CoA ligase [GDP-forming] subunit beta, mitochondrial	-0.44	-0.54	0.08	-0.29	0.72	-0.19
SUMO2_MOUSE	Small ubiquitin-related modifier 2		1.37				
SUOX_MOUSE	Sulfite oxidase, mitochondrial	-0.32	0.33	0.19	-0.35	-0.24	-1.39
SYFB_MOUSE	Phenylalanine--tRNA ligase beta subunit				0.19		
SYIM_MOUSE	Isoleucine--tRNA ligase, mitochondrial					0.47	
SYSC_MOUSE	Serine--tRNA ligase, cytoplasmic			0.07			
TADBP_MOUSE	TAR DNA-binding protein 43	1.82		0.01		0.40	
TAGL2_MOUSE	Transgelin-2	2.83	1.85	-0.14	1.99	1.27	0.45
TALDO_MOUSE	Transaldolase	-0.08	1.51	-1.36	4.77	0.92	-2.43
TBA1A_MOUSE	Tubulin alpha-1A chain		-1.83				

TBA1B_MOUSE	Tubulin alpha-1B chain	-1.24	-2.37	0.16	0.65	-0.66	0.99
TBA1C_MOUSE	Tubulin alpha-1C chain			0.34	0.30	-0.58	
TBA4A_MOUSE	Tubulin alpha-4A chain	-1.05	-1.47		0.55	-0.70	
TBB2A_MOUSE	Tubulin beta-2A chain			0.86	1.10		
TBB4B_MOUSE	Tubulin beta-4B chain	-0.92	-1.37	0.31	0.51	0.33	0.87
TBB5_MOUSE	Tubulin beta-5 chain					0.43	
TCPZ_MOUSE	T-complex protein 1 subunit zeta	-2.24	-1.52		0.33	-0.68	
TCTP_MOUSE	Translationally-controlled tumor protein	-9.97	0.92	0.16	1.29	0.75	0.09
TERA_MOUSE	Transitional endoplasmic reticulum ATPase	-0.47	-0.25	0.38	0.37	0.08	0.04
THIC_MOUSE	Acetyl-CoA acetyltransferase, cytosolic	-0.25	-0.13	0.66	0.01	0.20	-0.35
THIKA_MOUSE	3-ketoacyl-CoA thiolase A, peroxisomal	-1.81	-0.50	-0.28		-0.15	
THIKB_MOUSE	3-ketoacyl-CoA thiolase B, peroxisomal		-0.14	0.10	-0.87	0.11	-0.40
THIL_MOUSE	Acetyl-CoA acetyltransferase, mitochondrial	-0.17	0.74	0.26	-0.01	-0.03	-0.57
THIM_MOUSE	3-ketoacyl-CoA thiolase, mitochondrial	-1.56	-1.04	-0.05	-0.03	-0.49	-0.65
THIO_MOUSE	Thioredoxin	2.09	2.67	0.47	0.12	1.11	-0.70
THIOM_MOUSE	Thioredoxin, mitochondrial	2.85	3.44			-0.33	
THOC2_MOUSE	THO complex subunit 2	6.17					
THTM_MOUSE	3-mercaptopyruvate sulfurtransferase	-1.74	-0.97	-0.19	0.16	1.79	0.37
THTR_MOUSE	Thiosulfate sulfurtransferase	-1.02	-0.61	0.34	0.26	-0.14	-0.64
TIM13_MOUSE	Mitochondrial import inner membrane translocase subunit Tim13	0.83	0.58	-0.24			-0.04
TIM8A_MOUSE	Mitochondrial import inner membrane translocase subunit Tim8 A	1.12	1.29	0.29	0.47		
TIM8B_MOUSE	Mitochondrial import inner membrane translocase subunit Tim8 B	1.07	0.61				
TIM9_MOUSE	Mitochondrial import inner membrane translocase subunit Tim9		2.28				
TITIN_MOUSE	Titin	-3.37	-2.90	0.13		-0.61	0.75
TKT_MOUSE	Transketolase	0.05	-0.73	0.60	0.61	0.41	-0.19
TLN1_MOUSE	Talin-1	0.21		-1.17		0.56	
TM205_MOUSE	Transmembrane protein 205				-0.14		
TMEDA_MOUSE	Transmembrane emp24 domain-containing protein 10				0.77	-0.22	0.70
TPD54_MOUSE	Tumor protein D54	0.32					
TPIS_MOUSE	Triosephosphate isomerase	-0.19	-0.30	0.27	0.30	0.19	-0.07
TPM3_MOUSE	Tropomyosin alpha-3 chain	1.75	1.87	0.10		1.09	-0.36
TPM4_MOUSE	Tropomyosin alpha-4 chain	0.56	-0.64				

TPMT_MOUSE	Thiopurine S-methyltransferase	-0.34	0.15	0.79	2.33	0.90	
TRAP1_MOUSE	Heat shock protein 75 kDa, mitochondrial	-3.56					
TRFE_MOUSE	Serotransferrin	0.41	0.92	0.32	0.06	0.12	-1.14
TRXR1_MOUSE	Thioredoxin reductase 1, cytoplasmic		-0.91	0.62	-0.04		-0.58
TTC36_MOUSE	Tetratricopeptide repeat protein 36	-0.31	1.02				
TTC38_MOUSE	Tetratricopeptide repeat protein 38		-2.23		0.77		-0.21
TTHY_MOUSE	Transthyretin	-0.18	-0.69	0.04	-0.57	-0.20	-0.91
TTPA_MOUSE	Alpha-tocopherol transfer protein						-0.27
TXND5_MOUSE	Thioredoxin domain-containing protein 5	2.22	0.37	0.57	0.43	1.06	0.57
UBA1_MOUSE	Ubiquitin-like modifier-activating enzyme 1	-2.63			0.21		
UBR5_MOUSE	E3 ubiquitin-protein ligase UBR5					2.80	
UCRI_MOUSE	Cytochrome b-c1 complex subunit Rieske, mitochondrial		1.20				
UD11_MOUSE	UDP-glucuronosyltransferase 1-1						-0.75
UD17C_MOUSE	UDP-glucuronosyltransferase 1-7C		0.08				
UD19_MOUSE	UDP-glucuronosyltransferase 1-9	-3.97		1.16	0.53	0.14	
UD2A3_MOUSE	UDP-glucuronosyltransferase 2A3		-3.29		0.34	-1.59	-2.43
UDB17_MOUSE	UDP-glucuronosyltransferase 2B17	-2.73	-2.61		0.22	-0.75	0.25
UFM1_MOUSE	Ubiquitin-fold modifier 1		1.01				
UGDH_MOUSE	UDP-glucose 6-dehydrogenase	0.21	0.84	0.37	-0.30	1.47	-0.94
UGPA_MOUSE	UTP--glucose-1-phosphate uridylyltransferase			0.04	-0.72	-0.55	
UK114_MOUSE	Ribonuclease UK114	0.97	1.02	0.23	-0.10	0.54	-0.45
URIC_MOUSE	Uricase	-1.37	-0.47	0.22	0.48	0.58	-0.97
USMG5_MOUSE	Up-regulated during skeletal muscle growth protein 5					0.26	
VDAC1_MOUSE	Voltage-dependent anion-selective channel protein 1	-3.04	-3.22	0.37	0.16	-0.42	0.38
VDAC2_MOUSE	Voltage-dependent anion-selective channel protein 2				0.11	-0.68	-0.73
VIGLN_MOUSE	Vigilin	1.77	-0.85	1.50	0.03	-0.10	0.00
VIME_MOUSE	Vimentin	2.83	1.21	0.09	0.80	-2.78	1.30
VINC_MOUSE	Vinculin			-1.00	-0.82		
VKOR1_MOUSE	Vitamin K epoxide reductase complex subunit 1				0.40	-0.05	
VTDB_MOUSE	Vitamin D-binding protein	2.82	2.93	0.48	0.28	1.19	-1.13
VTNC_MOUSE	Vitronectin	1.35		1.71	1.18	-0.17	-0.03
YBOX1_MOUSE	Nuclease-sensitive element-binding protein 1	4.61		0.26		0.76	0.18

Each values are Log2 ratio of a pair of cell sheets transplanted mice and sham-operated mice liver tissue. Blank means either of a pair was not detected.

Supplementary Table 2. Proteins included in gene ontology term, oxidative reduction

Uniprot ID	Protein description	Log2[Peak area ratio (sheets／sham）]					
		day 2		day 3		day 4	
ACPM_MOUSE	Acyl carrier protein, mitochondrial	5.23	6.45	0.11			
THIOM_MOUSE	Thioredoxin, mitochondrial	2.85	3.44			-0.33	
NDUS4_MOUSE	NADH dehydrogenase [ubiquinone] iron-sulfur protein 4, mitochondrial	2.44	4.09				
QCR6_MOUSE	Cytochrome b-c1 complex subunit 6, mitochondrial	2.42	3.20	0.79	0.49	0.65	-0.13
THIO_MOUSE	Thioredoxin	2.09	2.67	0.47	0.12	1.11	-0.70
PRDX2_MOUSE	Peroxiredoxin-2	1.92	2.20	-0.03	0.35	1.91	-0.40
NDUS6_MOUSE	NADH dehydrogenase [ubiquinone] iron-sulfur protein 6, mitochondrial	1.87	2.40	0.17		0.29	-0.37
GLRX1_MOUSE	Glutaredoxin-1	1.79	2.09				
AK1D1_MOUSE	3-oxo-5-beta-steroid 4-dehydrogenase	1.38	0.53	1.11			
CYC_MOUSE	Cytochrome c, somatic	0.95	2.34	0.65	-0.86	0.71	-0.07
NDUV2_MOUSE	NADH dehydrogenase [ubiquinone] flavoprotein 2, mitochondrial	0.62	1.78	-0.53	-2.76	0.79	-1.70
QCR7_MOUSE	Cytochrome b-c1 complex subunit 7	0.38	2.39	-0.48			
NDUBA_MOUSE	NADH dehydrogenase [ubiquinone] 1 beta subcomplex subunit 10	0.25	0.41		0.15		
CYB5_MOUSE	Cytochrome b5	-0.16	0.06	0.41	0.29	-0.66	0.82
CP2DQ_MOUSE	Cytochrome P450 2D26	-0.77	-2.79	1.08	0.34	-0.54	0.09
CP2D9_MOUSE	Cytochrome P450 2D9	-1.10	-2.47	1.65	-0.14	-1.13	0.56
AL1A7_MOUSE	Aldehyde dehydrogenase, cytosolic 1	-1.65	-2.04	0.19	-0.41	-0.72	
COX2_MOUSE	Cytochrome c oxidase subunit 2	-1.92	-3.00	1.50	0.34	0.59	0.12
ALDH2_MOUSE	Aldehyde dehydrogenase, mitochondrial	-2.01	-1.19	0.34	0.26	-0.09	-1.14
ECHA_MOUSE	Trifunctional enzyme subunit alpha, mitochondrial	-2.10	-2.11	0.26	0.32	-0.31	-0.20
IDHP_MOUSE	Isocitrate dehydrogenase [NADP], mitochondrial	-2.11	-1.41	0.21	0.22	-0.19	-0.42
DECR_MOUSE	2,4-dienoyl-CoA reductase, mitochondrial	-2.12	-1.47	0.66	-0.34	-0.29	-0.45
CP2E1_MOUSE	Cytochrome P450 2E1	-2.16			1.85	-0.30	0.24
SSDH_MOUSE	Succinate-semialdehyde dehydrogenase, mitochondrial	-2.20	-1.80				
ETFD_MOUSE	Electron transfer flavoprotein-ubiquinone oxidoreductase, mitochondrial	-2.22	-1.66	-0.59	0.09	-1.33	0.11
CMC2_MOUSE	Calcium-binding mitochondrial carrier protein	-2.27	-3.15		-0.18	-0.93	

	Aralar2						
NDUA2_MOUSE	NADH dehydrogenase [ubiquinone] 1 alpha subcomplex subunit 2	-2.37	-1.96				
3HIDH_MOUSE	3-hydroxyisobutyrate dehydrogenase, mitochondrial	-2.38	-0.91	-0.58	-0.62	-0.28	-0.22
ACDSB_MOUSE	Short/branched chain specific acyl-CoA dehydrogenase, mitochondrial	-2.43					
PH4H_MOUSE	Phenylalanine-4-hydroxylase	-2.44	-2.91	0.78	0.16	-0.48	0.27
GPDA_MOUSE	Glycerol-3-phosphate dehydrogenase [NAD+], cytoplasmic	-2.47	-1.99	0.41	0.20	-0.17	-0.64
ACADS_MOUSE	Short-chain specific acyl-CoA dehydrogenase, mitochondrial	-2.52	-0.49	0.30	0.55	0.57	-0.99
ODBA_MOUSE	2-oxoisovalerate dehydrogenase subunit alpha, mitochondrial	-2.53	-1.47	0.53	-0.79		
CP2CT_MOUSE	Cytochrome P450 2C29	-2.55			0.50	-0.09	-0.87
MOSC2_MOUSE	MOSC domain-containing protein 2, mitochondrial	-2.56	-3.16		-0.11	0.27	0.20
ACADM_MOUSE	Medium-chain specific acyl-CoA dehydrogenase, mitochondrial	-2.56	-3.04	0.11	0.19	0.03	-0.24
AASS_MOUSE	Alpha-aminoadipic semialdehyde synthase, mitochondrial	-2.65	-2.47	0.92	-0.11	0.92	-1.60
ECHP_MOUSE	Peroxisomal bifunctional enzyme	-2.71	-2.19	-0.27	-0.66	-1.43	-0.40
CY1_MOUSE	Cytochrome c1, heme protein, mitochondrial	-2.78	-2.88		-0.25	-1.17	-0.27
FMO5_MOUSE	Dimethylaniline monooxygenase [N-oxide-forming] 5	-2.82	-4.92		-0.35	-1.21	-0.14
ACOX2_MOUSE	Peroxisomal acyl-coenzyme A oxidase 2	-2.84	-2.41	0.43	0.23	0.02	-0.28
CHDH_MOUSE	Choline dehydrogenase, mitochondrial	-2.85	-2.02				
DHI1_MOUSE	Corticosteroid 11-beta-dehydrogenase isozyme 1	-2.90	-2.10		0.17		
CP2DA_MOUSE	Cytochrome P450 2D10	-2.93	-2.82	0.46	0.08	-1.23	0.42
ODPA_MOUSE	Pyruvate dehydrogenase E1 component subunit alpha, somatic form, mitochondrial	-2.97	-0.90				
AIFM1_MOUSE	Apoptosis-inducing factor 1, mitochondrial	-2.98	-2.50	0.70	-0.50	-0.74	0.36
AL4A1_MOUSE	Delta-1-pyrroline-5-carboxylate dehydrogenase, mitochondrial	-3.06	-1.95	0.43	0.19	-0.21	-0.24
BDH_MOUSE	D-beta-hydroxybutyrate dehydrogenase, mitochondrial	-3.16	-2.98	1.11	0.58	-0.10	0.13
GCDH_MOUSE	Glutaryl-CoA dehydrogenase, mitochondrial	-3.16	-2.08	0.45	-0.13	-0.46	-0.30
HAOX1_MOUSE	Hydroxyacid oxidase 1	-3.28		-0.11	0.29	-2.49	-0.53
DPYD_MOUSE	Dihydropyrimidine dehydrogenase [NADP+]	-3.35	-1.60				

ACADV_MOUSE	Very long-chain specific acyl-CoA dehydrogenase, mitochondrial	-3.54	-2.77	0.14	0.35	-1.11	-1.38
3BHS5_MOUSE	3 beta-hydroxysteroid dehydrogenase type 5	-3.64					
CP2F2_MOUSE	Cytochrome P450 2F2	-3.78	-5.51		-1.65	-2.52	
ODBB_MOUSE	2-oxoisovalerate dehydrogenase subunit beta, mitochondrial	-3.92		-0.16	-0.83		
QCR8_MOUSE	Cytochrome b-c1 complex subunit 8	-4.04	-4.44		0.07		
FAS_MOUSE	Fatty acid synthase	-4.08	-2.41	1.22	1.20	-0.52	0.85
NDUV1_MOUSE	NADH dehydrogenase [ubiquinone] flavoprotein 1, mitochondrial	-4.11	-2.72	-0.32	0.01	-1.98	-1.47
DHB13_MOUSE	17-beta-hydroxysteroid dehydrogenase 13	-4.11	-3.92	1.02	0.03	-1.16	1.01
ODO1_MOUSE	2-oxoglutarate dehydrogenase, mitochondrial	-4.13	-2.98	0.14	0.39	-0.07	0.33
ODB2_MOUSE	Lipoamide acyltransferase component of branched-chain alpha-keto acid dehydrogenase complex, mitochondrial	-4.44	6.51	0.76	-0.10	1.04	0.20
CP341_MOUSE	Cytochrome P450 3A41	-4.68					
ATP8_MOUSE	ATP synthase protein 8	-5.21	-3.94		-0.48		
CBR4_MOUSE	Carbonyl reductase family member 4	-5.51	-9.97				
CP27A_MOUSE	Sterol 26-hydroxylase, mitochondrial	-9.97					
KMO_MOUSE	Kynurenine 3-monooxygenase	-9.97	-2.65		0.20	-0.32	
CP3AB_MOUSE	Cytochrome P450 3A11		-9.97		1.64	-1.78	
CP4CA_MOUSE	Cytochrome P450 4A12A		-4.80		-0.49	-0.77	
NB5R3_MOUSE	NADH-cytochrome b5 reductase 3		-3.07	-0.50	-0.29	-1.14	
GSH0_MOUSE	Glutamate--cysteine ligase regulatory subunit		-0.62				
MICA3_MOUSE	Protein MICAL-3					1.22	-9.97
MAOX_MOUSE	NADP-dependent malic enzyme		-9.97		0.50		
RDH7_MOUSE	Retinol dehydrogenase 7		-3.59		0.81	-0.28	
CP254_MOUSE	Cytochrome P450 2C54			2.12		1.27	

Extracted proteins of oxidative reduction, biological process of GO term, are listed. Each values are Log2 ratio of a pair of cell sheets transplanted mice and sham-operated mice liver tissue. Blank means either of a pair was not detected.

Supplementary Table 3. Sequences of primer for conformation of hepatic differentiation

Primers		sequence (5'-3')	positive control
Albumin	sense	TTGGAAAAATCCCACTGCAT	Huh7 cells
	antisense	CTCCAAGCTGCTCAAAAAGC	
C3	sense	CAGCACCATGGGACCCACCTCAG	Huh7 cells
	antisense	CTCTCCAGCCGCAAGATGTTGGG	
C4	sense	ACTTTGAGACCGAGGGGCCC	Huh7 cells
	antisense	GGCACTTCCTGCACAGCCTC	
Apolipoprotein E	sense	GTCCTTCCCCAGGAGCCGAC	Huh7 cells
	antisense	GTCTCCACCGCTTGCTCCAC	
α1-antitrypsin	sense	CAAGGAGCTTGACAGAGACACAGTTTTT	Huh7 cells
	antisense	GTGTCCTTGACTTCAAAGGGTCTCT	
Vimentin	sense	AATGCGTCTCTGGCACGTC	UE7T-13 cells
	antisense	CTTCTGCCTCCTGCAGGTTC	
N-cadherin	sense	CCGACGAATGGATGAAAGACC	UE7T-13 cells
	antisense	TTGCAGCCTATGCCAAAGC	
Nanog	sense	CTGTGATTTGTGGGCCTGAA	201B7
	antisense	TGTTTGCCTTTGGGACTGGT	
GAPDH	sense	GTCTTCTCCACCATGGAGAAGGCT	Huh7 cells
	antisense	CATGCCAGTGAGCTTCCCGTTCA	

Supplementary Table 4. A List of primers for analysis of human-specific gene expression of serum proteins in grafted tissues

**Primers**		**sequence (5'-3')**	**positive control**
Albumin	sense	TTGGAAAAATCCCACTGCAT	Huh7 cells
	antisense	CTCCAAGCTGCTCAAAAAGC	
Ceruloplasmin	sense	CGACTTGGGATTATGCCTCTGACC	Huh7 cells
	antisense	CCCAATTCTATCTGGGCCATTTTGA	
A1AT	sense	CAAGGAGCTTGACAGAGACACAGTTTTT	Huh7 cells
	antisense	GTGTCCTTGACTTCAAAGGGTCTCT	
Transferrin	sense	GGGCTATGAGTATGTCACTGC	Huh7 cells
	antisense	ATCACACTTGAGCCTCTCG	
APOE	sense	GTCCTTCCCCAGGAGCCGAC	Huh7 cells
	antisense	GTCTCCACCGCTTGCTCCAC	
C3	sense	CAGCACCATGGGACCCACCTCAG	Huh7 cells
	antisense	CTCTCCAGCCGCAAGATGTTGGG	
C4	sense	ACTTTGAGACCGAGGGGCCC	human Liver
	antisense	GGCACTTCCTGCACAGCCTC	
RBP4	sense	CCCAGAAGCGCAGAAGATT	Huh7 cells
	antisense	AAGGTTTCTTTCTGATCTGCCAT	
human GAPDH	sense	AGCCACATCGCTCAGACAC	UE7T-13 cells
	antisense	GCCCAATACGACCAAATCC	
mouse β-actin	sense	GCGAGCACAGCTTCTTTG	mouse Liver
	antisense	CGCAGCGATATCGTCATCC	

A1AT, alpha 1-antitrypsin; APOE, apolipoprotein E; RBP4, retinol binding protein 4

Supplementary Table 5. A List of primers for analysis of human-specific gene expression of cytokines in grafted tissues

**Primers**		**sequence (5'-3')**	**positive control**
TNFα	sense	CCCAGGGACCTCTCTCTAATC	Leukocyte
	antisense	GGCCAGGAGGGCATTG	
IL-6	sense	CCAGAGCTGTGCAGATGAG	UE7T-13 cells
	antisense	GTCAGCAGGCTGGCATTT	
EGF	Sense	TGGGTCAAGGCAAGAGAGAGTA	Huh-7 cells
	antisense	GATTCCTTCCTGTTGATTTGACCA	
HB-EGF	sense	GGACCGGAAAGTCCGT	Huh-7 cells
	antisense	GCTCCTCCTTGTTTGGTGT	
AREG	sense	AACGAAAGAAACTTCGACAAGAGA	Huh-7 cells
	antisense	ATGATCCACTGGAAAGAGGACC	
TGFα	sense	CCTCTCTCTAATCAGCCCTCTG	UE7T-13 cells
	antisense	GAGGACCTGGGAGTAGATGAG	
HGF	sense	CCCTTCAATAGCATGTCAAGTGG	UE7T-13 cells
	antisense	GTTCCCTTGTAGCTGCGT	
SCF	sense	AGGGACAGTGGAGAGGG	UE7T-13 cells
	antisense	GCAAGTGAGAATCCAAGTTTGTGT	
bFGF	sense	GGGTCCGGGAGAAGAGC	UE7T-13 cells
	antisense	GCCAGGTAACGGTTAGCAC	
VEGF	sense	TTGCCTTGCTGCTCTACCT	UE7T-13 cells
	antisense	TCCATGAACTTCACCACTTCGT	
Angiopoietin	sense	ATGTGCAAATGTGCCCTCA	UE7T-13 cells
	antisense	TCGCTTCTGACATTGCGCT	
Angiogenin	sense	TTCCATTGTCCTGCCCG	UE7T-13 cells
	antisense	AAGTGTGTGTACCTGGAGTTATC	
human GAPDH	sense	AGCCACATCGCTCAGACAC	UE7T-13 cells
	antisense	GCCCAATACGACCAAATCC	
mouse β-actin	sense	GCGAGCACAGCTTCTTTG	mouse Liver
	antisense	CGCAGCGATATCGTCATCC	

TNFα, Tumor Necrosis Factor alpha; IL-6, intrleukin-6; EGF, epidermal growth factor; HB-EGF, heparin binding-epidermal growth factor-like growth factor; AREG, amphiregulin; TGFα, Transforming growth factor alpha; HGF, hepatocyte growth factor; SCF, stem cell factor; bFGF, basic fibroblast growth factor; VEGF, vascular

endothelial growth factor

Supplementary Table 6. A List of primers for analysis of mouse gene expression of cytokines in recipient and sham-operated mice liver

Primers		sequence (5'-3')	positive control
TNFα	sense	AAGCCTGTAGCCCACGTCGTA	mouse bone marrow
	antisense	GGCACCACTAGTTGGTTGTCTTTG	
IL-10	sense	AGTGAGAAGCTGAAGACCCTCAGG	mouse bone marrow
	antisense	TTCATGGCCTTGTAGACACCTTGGT	
IL-1α	sense	TCTCAGATTCACAACTGTTCGTG	mouse bone marrow
	antisense	AGAAAATGAGGTCGGTCTCACTA	
IL-1β	sense	GAAATGCCACCTTTTGACAGTG	mouse bone marrow
	antisense	TGGATGCTCTCATCAGGACAG	
IL-1ra	sense	TAGACATGGTGCCTATTGACCT	mouse bone marrow
	antisense	TCGTGACTATAAGGGGCTCTTC	
IL-2	sense	CCGCAGAGGTCCAAGTTCAT	mouse bone marrow
	antisense	CTCCTGAGCAGGATGGAGAAT	
HGF	sense	AGAAATGCAGTCAGCACCATCAAG	mouse bone marrow
	antisense	GATGGCACATCCACGACCAG	
c-met	sense	GTGCCAAGCTACCAGT	mouse Liver
	antisense	CTTCGTACAAGGCGTCT	
VEGFα	sense	TTACTGCTGTACCTCCACC	mouse bone marrow
	antisense	ACAGGACGGCTTGAAGATG	
VEGFR2	sense	CCCCAAATTCCATTATGACAA	mouse Liver
	antisense	CGGCTCTTTCGCTTACTGTT	
Angiopoietin1	sense	TATTTTGTGATTCTGGTGATT	mouse bone marrow
	antisense	GTTTCGCTTTATTTTTGTAATG	
Angiopoietin2	sense	TCCAAGAGCTCGGTTGCTAT	mouse bone marrow
	antisense	AGTTGGGGAAGGTCAGTGT	
Angiogenin	sense	TCCTGAAGTTGGCCTGAACG	mouse bone marrow
	antisense	CAACAGAGATTCCAAAGCTGGC	
PECAM	sense	CTGAGGAAAACTCCTTCACCATC	mouse Liver
	antisense	ACCTTCACCTCGTACTCAATCGT	
TGFα	sense	CTGGCTGCAGCACCCTGCGCT	mouse Liver
	antisense	GATCAGCACACAGGTGATAATGAGGAC	
EGF	sense	AGAGGGCTTTTACCTGAATCACA	mouse bone marrow
	antisense	CTTGGCATTTGTCACACATGG	

HB-EGF	sense	CGGGGAGTGCAGATACCTG	mouse bone marrow
	antisense	TTCTCCACTGGTAGAGTCAGC	
Amphiregulin	sense	TTGAGCTTTCTGTGGGAAGAGAGG	mouse Liver
	antisense	TTGTCATCCTCGCTGTGAGTCTTC	
SCF	sense	TCTGCGGGAATCCTGTGACT	mouse bone marrow
	antisense	CGGCGACATAGTTGAGGGTTAT	
IGF1	sense	ACTGACATGCCCAAGACTCAGAAGTC	mouse bone marrow
	antisense	TGCCTCCGTTACCTCCTCCTGTTC	
SDF1	sense	GCATCAGTGACGGTAAACCAG	mouse bone marrow
	antisense	GGGTCAATGCACACTTGTCTG	
TGFβ1	sense	GTGGAAATCAACGGGATCAG	mouse bone marrow
	antisense	ACTTCCAACCCAGGTCCTTC	
BMP7	sense	AGAAAACAGCAGCAGTGAC	mouse bone marrow
	antisense	TTAGATGGTGAGACATCGAAG	
IGFBP3	sense	TCCAGGAAACATCAGTGAGTCCGA	mouse bone marrow
	antisense	CATACTTGTCCACACACAGCAGA	
IGFBP8	sense	TGACCTGGAGGAAAACATTAAGA	mouse bone marrow
	antisense	AGCCCTGTATGTCTTCACACTG	
KGF	sense	ACACGGATCCTGCCAACTCT	mouse bone marrow
	antisense	GTTTGCTCCGGACTCATGTCA	
β-catenin	sense	ATGGAGCCGGACAGAAAAGC	mouse bone marrow
	antisense	CTTGCCACTCAGGGAAGGA	
TIMP1	sense	AGGTGGTCTCGTTGATTTCT	mouse Liver
	antisense	GTAAGGCCTGTAGCTGTGCC	
TIMP3	sense	GCGGAAGCGTGCACATG	mouse bone marrow
	antisense	GCTTCTTTCCCACCACTTTGG	
mouse β-actin	sense	GCGAGCACAGCTTCTTTG	mouse Liver
	antisense	CGCAGCGATATCGTCATCC	

TNFα, Tumor Necrosis Factor alpha; IL-10, interleukin-10; IL-1α, interleukin-1 alpha; IL-1β, interleukin-1 beta, IL-1ra, interleukin-1 receptor antagonist; IL-2, intrleukin-2; HGF, hepatocyte growth factor; VEGFα, vascular endothelial growth factor alpha; VEGFR2, vascular endothelial growth factor receptor 2; PECAM, Platelet endothelial cell adhesion molecule-1; TGFα, Transforming growth factor alpha; EGF, epidermal growth factor; HB-EGF, heparin binding-epidermal growth factor-like growth factor; SCF, stem cell factor; IGF1, insulin-like growth factor 1; SDF1, stromal cell-derived factor 1; TGFβ1, transforming growth factor beta 1; BMP7, bone morphogenetic protein 7; IGFBP3, insulin-like growth factor binding protein 3;

IGFBP8, insulin-like growth factor binding protein 8; KGF, keratin growth factor; TIMP1, tissue inhibitor of metalloproteinase 1; TIMP3, tissue inhibitor of metalloproteinase 3
